# Bioinformatics and survival analysis of glia maturation factor-γ in pan-cancers

**DOI:** 10.1186/s12885-021-08163-2

**Published:** 2021-04-17

**Authors:** Aihua Lan, Chunxia Ren, Xiaoling Wang, Guoqing Tong, Gong Yang

**Affiliations:** 1 Central Laboratory, the Fifth People’s Hospital of Shanghai, Fudan University, Shanghai, 200240 China; 2 Center for Reproductive Medicine, Shuguang Hospital Affiliated to Shanghai University of Traditional Chinese Medicine, Shanghai, 200120 China; 3 Cancer Institute, Fudan University Shanghai Cancer Center, Department of Oncology, Fudan University Shanghai Medical College, Shanghai, 200032 China

**Keywords:** Glia maturation factor-γ, Cancer, Survival, Bioinformatics, Immune response, Tumor microenvironment

## Abstract

**Background:**

Glia maturation factor-γ (GMFG) is reported to inhibit the actin nucleation through binding to the actin-related protein-2/3 complex (Arp2/3). Considering the main function of GMFG in actin remodeling, which is vital for immune response, angiogenesis, cell division and motility, GMFG is supposed to have important roles in tumor development, while up to now, only two studies described the role of GMFG in cancers. By investigating the clinical values of GMFG using The Cancer Genome Atlas (TCGA) data and the functional mechanisms of GMFG through analyses of Gene Ontology (GO) and Kyoto Encyclopedia of Genes and Genomes (KEGG) pathway enrichments, this study was aimed to better understand the impact of GMFG in pan-cancers and to draw more attentions for the future research of GMFG.

**Methods:**

RNA-seq and clinical data of cancer patients were collected from TCGA and analyzed by the Kaplan-Meier methods. GO and KEGG analyses were conducted using the online tools from the Database for Annotation, Visualization and Integrated Discovery (DAVID).

**Results:**

Compared to the corresponding normal samples, GMFG was significantly upregulated in glioblastoma (GBM), kidney clear cell carcinoma (KIRC), lower grade glioma (LGG), acute myeloid leukemia (LAML), and pancreatic cancer (PAAD), testicular cancer (TGCT), but was downregulated in kidney chromophobe (KICH), lung adenocarcinoma (LUAD) and lung squamous cell carcinoma (LUSC) (*P* < 0.05 for all). High expression of GMFG predicted worse OS in GBM (HR = 1.5, *P* = 0.017), LGG (HR = 2.2, *P* < 0.001), LUSC (HR = 1.4, *P* = 0.022) and ocular melanomas (UVM) (HR = 7, P < 0.001), as well as worse DFS in LGG (HR = 1.8, P < 0.001) and prostate cancer (PRAD) (HR = 1.9, *P* = 0.004). In contrast, high expression of GMFG was associated with better OS in skin cutaneous melanoma (SKCM) (HR = 0.59, *P* < 0.001) and thymoma (THYM) (HR = 0.098, *P* = 0.031), as well as better DFS in bile duct cancer (CHOL) (HR = 0.2, *P* = 0.003). GMFG was mainly involved in the immune response, protein binding and cytokine-cytokine receptor interaction pathways, and was positively associated with multiple immunomodulators in most cancers.

**Conclusion:**

Our study preliminarily identified that GMFG may cause different survivals for different cancers through modulating tumor progression, immune response status and tissue-specific tumor microenvironment (TME).

**Supplementary Information:**

The online version contains supplementary material available at 10.1186/s12885-021-08163-2.

## Introduction

Glia maturation factor-γ (GMFG) is a 17 kDa small protein, and its gene sequence is conserved from yeast to mammalian. GMFG was initially identified as a glia maturation factor that can induce brain cell differentiation [1, 2]. Later reports found that GMFG can regulate the actin cytoskeleton because it actually belongs to the actin-depolymerizing factor homology (ADF-H) family [3, 4]. All ADF-H proteins can remodel actin cytoskeleton through binding to either actin and/or actin-related proteins (Arps) [5], and the reorganization of actin cytoskeleton is crucial for immune system function, angiogenesis and cell motility [6]. As a novel regulator of the Arp2/3 complex, GMFG is reported to enhance the angiogenic sprouting of zebrafish [7] and to regulate the migration of airway smooth muscle cell [8–10], as well as to promote the chemotaxis of neutrophils and T lymphocytes [11, 12].

Considering the main function of GMFG in actin remodeling vital for cancer immunity, angiogenesis, cell division and motility [13, 14], GMFG is supposed to have important roles in tumor development, while up to now, only two studies described the role of GMFG in cancers [15, 16]. These two studies demonstrated that GMFG could promote the migration and proliferation of ovarian and colorectal cancer cells, and the high expression of GMFG was related to poor survival outcome for ovarian cancer patients. To better understand the potential function of GMFG in pan-cancers and to draw more attention to GMFG for the future cancer research, in this study, we investigated the clinical values of GMFG using The Cancer Genome Atlas (TCGA) data, and analyzed the underlying functional mechanisms of GMFG through conducting Gene Ontology (GO) and Kyoto Encyclopedia of Genes and Genomes (KEGG) pathway enrichments. Furthermore, we also evaluated the impact of GMFG on cancer immunity by analysis of the association between GMFG and immunomodulators.

## Materials and methods

### Expression and survival analysis

We used Gene Expression Profiling Interactive Analysis (GEPIA) (http://gepia.cancer-pku.cn/) to compare the GMFG expression between tumor and normal samples [17]. Tumor samples were matched TCGA and Genotype-Tissue Expression (GTEx) normal samples [18], and log2 (TPM + 1) was used for log-scale. Moreover, the relationship between GMFG expression and pathological stage was investigated in the GEPIA database. Survival analyses that include overall survival (OS) and disease-free survival (DFS) were also conducted in the GEPIA database. Patients were divided into two groups (high and low expression groups) according to the median expression level of GMFG in cancer samples, and the survival curves were generated by the Kaplan-Meier methods. The hazard ratio (HR) and *P*-value were also calculated. The *P* value< 0.05 indicated statistical significance.

### GMFG-associated co-expression genes

To evaluate the potential functional mechanism of GMFG in cancers, we obtained GMFG-associated co-expression genes from the Multi-Experiment Matrix (MEM) (http://biit.cs.ut.ee/mem) [19] and cBioPortal (http://www.cbioportal.org) databases. In MEM database, the associations between GMFG and other genes were evaluated by the score/*P*-value. Genes with *P*-value smaller than 0.0001 were selected. Meanwhile, the correlations between GMFG and co-expression genes were calculated by the Pearson’s correlation analysis in the cBioPortal database, and genes with correlation coefficient (absolute value) more than 0.5 were selected. Finally, the intersection analysis (Venny 2.1, https://bioinfogp.cnb.csic.es/tools/venny/) was conducted using the selected co-expression genes from both MEM and cBioPortal to acquire the overlapping genes for further analyses.

### Functional and pathway enrichment

To investigate the functional mechanism of GMFG in pan-cancers, the overlapping co-expression genes from MEM and cBioPortal were used for GO and KEGG pathway [20] enrichment analyses. Firstly, the GO and KEGG analyses for each cancer were conducted in Database for Annotation Visualization and Integrated Discovery (DAVID) v6.8 (https://david.ncifcrf.gov), and GO terms and KEGG pathways with *P*-value< 0.05 were considered as significant enrichments. Thus, significant GO/KEGG pathways for 32 different cancers were obtained. Then, Venny 2.1 was used for intersection analysis to acquire the top 20 overlapping GO/KEGG pathways among 32 cancers. Finally, the number of co-expression genes for each pathway in each cancer was shown in heatmap.

### GMFG and immunomodulators

Corrections between GMFG with its immunomodulators co-expression genes among 32 cancers were analyzed. The detailed categories of immunomodulators were obtained from the *Immune Landscape of Cancer* [21] which divided immunomodulators into seven types: co-stimulator, co-inhibitor, ligand, receptor, cell adhesion, antigen presentation and other. Heatmap was applied to show the correlation between GMFG and immunomodulators. Heatmaps of this study were generated by R language (https://www.r-project.org), other two packages including ggplot2 (version 3.3.3) and ComplexHeatmap (version 2.6.2) were also used to build heatmaps.

## Results

### Expression of GMFG in 33 different cancers

**Fig. 1:**
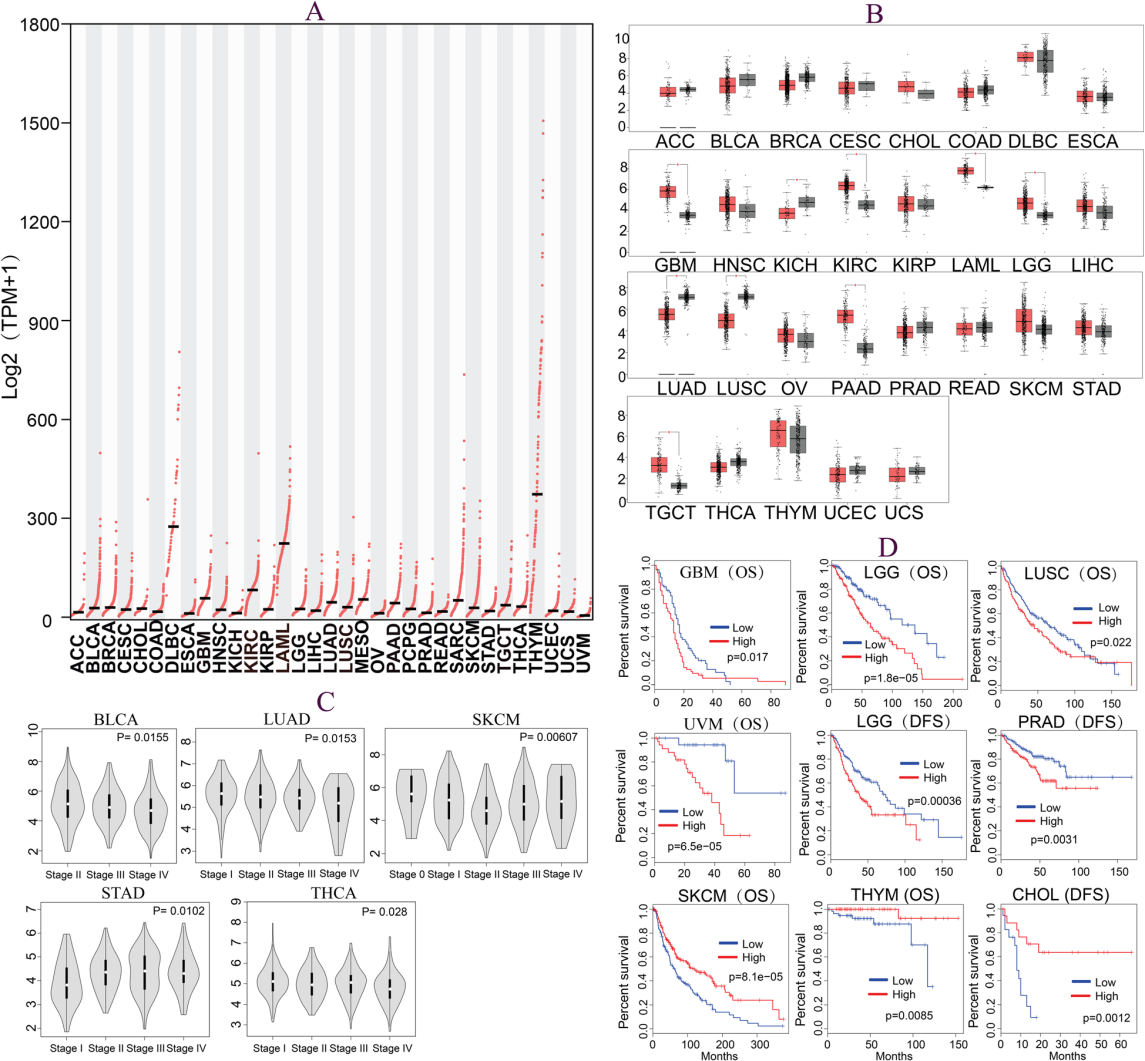
GMFG expressions of 33 types of cancer in TCGA datasets (**a**). GMFG expressions between cancer samples and corresponding normal samples in 29 different cancers (**b**). The associations between GMFG expression and pathological stages in five cancers (**c**). The Kaplan-Meier survival curves of GMFG high vs. GMFG low in nine cancer types (**d**)

Among 33 different cancers samples, GMFG expressions in lymphoid neoplasm diffuse large B-cell lymphoma (DLBC), acute myeloid leukemia (LAML) and thymoma (THYM) were obviously higher than that in other cancer types (Fig. 1a). The expression values of GMFG in 29 different cancers and corresponding normal samples were shown in Fig. 1b, four cancers (mesothelioma (MESO), pheochromocytoma and paraganglioma (PCPG), sarcoma (SARC), and uveal melanoma (UVM)) were excluded from the expression profile analysis due to small or a lack of corresponding normal samples. We found that GMFG was significantly upregulated in glioblastoma (GBM), kidney clear cell carcinoma (KIRC), lower grade glioma (LGG), LAML, and pancreatic cancer (PAAD), testicular cancer (TGCT), but was downregulated in kidney chromophobe (KICH), lung adenocarcinoma (LUAD) and lung squamous cell carcinoma (LUSC) (*P* < 0.05 for all).

### Pathological stage and survival analysis

As shown in Fig. 1c, high expression of GMFG was correlated with early pathological stage in bladder cancer (BLCA) (*P* = 0.016), LUAD (*P* = 0.015), skin cutaneous melanoma (SKCM) (*P* = 0.006) and thyroid cancer (THCA) (P = 0.01), but was linked with advanced pathological stage in stomach cancer (STAD) (*P* = 0.028). For survival outcome, high expression of GMFG predicted worse OS in GBM (HR = 1.5, *P* = 0.017), LGG (HR = 2.2, *P* < 0.001), LUSC (HR = 1.4, *P* = 0.022) and UVM (HR = 7, *P* < 0.001), as well as worse DFS in LGG (HR = 1.8, *P* < 0.001) and prostate cancer (PRAD) (HR = 1.9, *P* = 0.004). In contrast, high expression of GMFG was associated with better OS in SKCM (HR = 0.59, *P* < 0.001) and THYM (HR = 0.098, *P* = 0.031), as well as better DFS in bile duct cancer (CHOL) (HR = 0.2, *P* = 0.003) (Fig. 1d).

### GO and KEGG pathway enrichment analyses

**Fig. 2:**
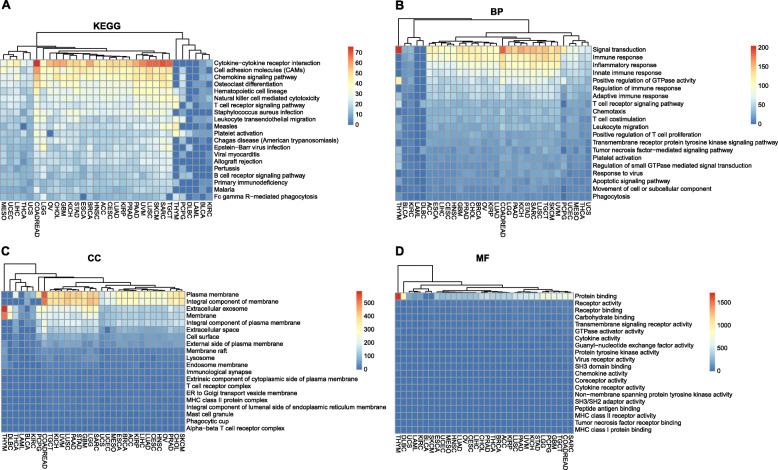
The intersection of GO and KEGG pathways among 32 cancer types, KEGG (**a**), biological process (BP) (**b**), cellular component (CC) (**c**) and molecular function (MF) (**d**)

As demonstrated in Fig. 2a, GMFG was mainly involved in cytokine-cytokine receptor interaction, cell adhesion molecules and chemokine signaling pathways in most cancers except for THYM, PCPG, DLBC, LAML, BLCA, and KIRC. The GO analysis indicated that GMFG was mainly correlated with the biological processes of signal transduction, immune response and inflammatory response in all cancer types with the exception of BLCA, KIRC, LAML and DLBC (Fig. 2b). For cellular component (Fig. 2c), GMFG was mainly enriched in the plasma membrane and integral component of membrane in the majority of 32 cancer types. For molecular function (Fig. 2d), GMFG was strongly related to protein binding in all cancers. Instead of enriching in immune response and plasma membrane, GMFG was linked with the biological processes of translation or insulin receptor signaling pathway, and was mainly concentrated on nucleoplasm or extracellular exosome in THYM, LAML and DLBC (Supplementary file 1–4: Table S1-S4).

### Correlation of GMFG and immunomodulators

**Fig. 3:**
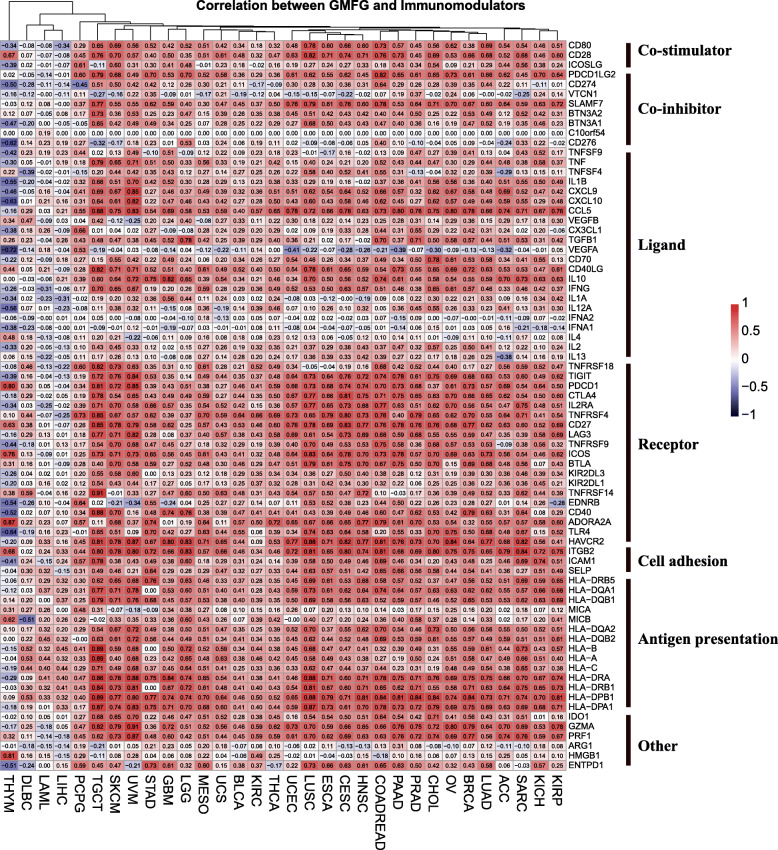
Association between GMFG and seven types of immunomodulators in 32 cancer types

The intersective co-expression genes in the top two significant biological processes and KEGG pathways, as well as top one cellular component and molecular function were acquired, and we listed the top 10 overlapping co-expression genes with their corresponding functions in Table 1. We also summarized the top 10 most significant items of GO/KEGG pathways for each individual cancer (Supplementary file 1–4: Table S1-S4), and we found that GMFG was notably associated with the biological process of immune response in most cancers, so we decided to analyze the correlation between GMFG and its immunomodulators co-expression genes. As shown in Fig. 3, GMFG was positively correlated with immunomodulators genes in most cancer, and the most positive correlations between GMFG and these genes were found in TGCT, SKCM, UVM, LUSC and colon and rectal cancer (COADREAD). As for co-stimulators, GMFG was highly associated with CD80 and CD28 in most cancers. In terms of co-inhibitors, GMFG showed a positive relationship with PDCD1LG2 (PD-L2) and SLAMF7 in most cancers. For cellular ligands, CXCL9, CXCL10, CCL5, CD40LG, IL10 and IFNG had a stronger relationship with GMFG than other ligands. For receptors, TIGIT, PDCD1 (PD-1), CTLA4, IL2RA, TNFRSF4, CD27, LAG3, ICOS, BTLA, ADORA2A and HAVCR2 were highly associated with GMFG in most cancers. As for cell adhesion molecules, GMFG was strongly correlated with ITGB2, followed by ICAM1 and SELP. Regarding MHC molecules, we found that the MHC class II molecules including HLA-DRA, HLA-DRB1, HLA-DPB1 and HLA-DPA1 exhibited a stronger association with GMFG than other MHC molecules. Finally, IDO1 and the cytotoxic molecules including GZMA and PRF1 showed a positively stronger association with GMFG in most cancer types. Interestingly, GMFG showed a significantly positive association with PD-1 and PD-L2, but not with CD274 (PD-L1) in most cancers. In contrast, GMFG was negatively correlated with most of the immunomodulators in THYM, and only a week correlation was observed between GMFG and immunomodulators in DLBC, LAML, and liver cancer (LIHC).

**Table 1:** The top 10 overlapping co-expression genes and the corresponding functions in the top two significant GO/KEGG pathways

Category	Top 10 genes	Functions	Correlation
KEGG_PATHWAY hsa04060: Cytokine-cytokine receptor interaction	CSF2RB	**Colony-stimulating factor 2** (granulocyte, macrophage), IL3, IL5 receptor common subunit beta (high affinity), critical for the activation of both the JAK/STAT (JAK2, STAT5) and MAP kinase pathways, involved in LYN binding.	0.72
	CCL5	**C-C motif chemokine ligand 5**, chemoattractant for blood monocytes, memory T-helper cells and eosinophils, may activate several chemokine receptors including CCR1, CCR3, CCR4 and CCR5.	0.80
	CXCR3	**C-X-C motif chemokine receptor 3,** binding CXCL9/Mig, CXCL10/IP10 and CXCL11/I-TAC induce cellular responses that are involved in leukocyte traffic, most notably integrin activation, cytoskeletal changes and chemotactic migration.	0.75
	LTB	**Lymphotoxin beta**, a type II membrane protein of the TNF family, and an inducer of the inflammatory response and involved in normal development of lymphoid tissue.	0.55
	CD27	A member of the TNF-receptor superfamily, is required for generation and long-term maintenance of T cell immunity. It binds to ligand CD70, and plays a key role in regulating B-cell activation and immunoglobulin synthesis.	0.68
	Others	IL10RA, IL3RA, IL12RB1, CSF1R, CCR5.	/
KEGG_PATHWAY hsa04514: Cell adhesion molecules (CAMs)	PECAM1	**Platelet and endothelial cell adhesion molecule 1**, makes up a large portion of endothelial cell intercellular junctions, and might involve in leukocyte migration, angiogenesis, and integrin activation.	0.77
	CD4	This gene encodes a membrane glycoprotein of T lymphocytes that interacts with MHC II antigens and initiates or augments the early phase of T-cell activation.	0.74
	CD2	The protein encoded by this gene is a surface antigen found on all peripheral blood T-cells. The encoded protein interacts with LFA3 (CD58) on antigen presenting cells to optimize immune recognition.	0.80
	ITGB2	**Integrin subunit beta 2**, combines with multiple different alpha chains to form different integrin heterodimers. Integrins are cell-surface proteins that participate in cell adhesion as well as cell-surface mediated signaling.	0.75
	ITGAL	**Integrin subunit alpha L**, combines with ITGB2 to form integrins.	0.73
	Others	SELPLG, CD86, HLA-DPB1, HLA-DPA1, CD40LG.	/
GOTERM_BP_DIRECT GO:0007165 ~ signal transduction	PECAM1	As presented above.	0.77
	RASAL3	**RAS Protein Activator Like 3**, a Ras GTPase-activating protein, plays an important role in the expansion and functions of natural killer T cells in the liver by negatively regulating RAS activity and the down-stream ERK signaling pathway.	0.75
	TYROBP	**TYRO protein tyrosine kinase binding protein**, non-covalently associates with activating receptors of the CD300 family. Cross-linking of CD300-TYROBP complexes results in cellular activation, involves in neutrophil activation mediated by integrin.	0.91
	DOK2	**Docking Protein 2** provides a docking platform for the assembly of multimolecular signaling complexes, may modulate the cellular proliferation induced by IL-4, IL-2 and IL-3. DOK2 may involve in modulating Bcr-Abl signaling and inhibit EGF-stimulated MAP kinase activation.	0.79
	ARHGAP9	Rho GTPase Activating Protein 9, a member of the Rho-GAP family of GTPase activating proteins, has substantial GAP activity towards several Rho-family GTPases, converting them to an inactive GDP-bound state. It implicates in regulating adhesion of hematopoietic cells to the extracellular matrix.	0.79
	Others	ARHGAP15, CD4, CD48, CD79B, CD33.	/
GOTERM_BP_DIRECT GO:0006955 ~ immune response	CD4	As presented above.	0.74
	CST7	**Cystatin-7**, a cysteine protease inhibitor with a role in immune regulation through inhibition of the hematopoietic system. Additionally, CST7 promotes metastasis in various cancers.	0.71
	WAS	**WASP actin nucleation promoting factor,** involves in transduction of signals from receptors on the cell surface to the actin cytoskeleton. WAS associates with the small GTPase, Cdc42 (regulates formation of actin filaments) and the cytoskeletal organizing complex, Arp2/3.	0.81
	CCL5	As presented above.	0.80
	VAV1	**Vav guanine nucleotide exchange factor 1**, activates pathways leading to actin cytoskeletal rearrangements and transcriptional alterations. VAV1 is important in hematopoiesis, T-cell and B-cell development and activation.	0.53
	Others	CD79B, LST1, NCF4, S1PR4, LCP2.	/
GOTERM_CC_DIRECT GO:0005886 ~ plasma membrane	HCST	**Hematopoietic cell signal transducer,** associates with KLRK1 to trigger cytotoxicity against target cells. KLRK1-HCST receptor plays a role in immune surveillance against tumors and is required for cytolysis of tumors cells, tumor cells that do not express HCST escape from immune surveillance mediated by NK cell.	0.86
	TYROBP	As presented above.	0.91
	PECAM1	As presented above.	0.77
	CD2	As presented above.	0.80
	CORO1A	**Coronin 1A**, a crucial component of the cytoskeleton of highly motile cells, functioning in the invagination and protrusions of plasma membrane involved in cell locomotion. CORO1A involves in a variety of cellular processes, including cell cycle progression, signal transduction, apoptosis, and gene regulation.	0.71
	Others	CD3D, CD3E, CD79B, GNGT2, KLRB1.	/
GOTERM_MF_DIRECT GO:0005515 ~ protein binding	HCST	As presented above.	0.86
	TNFAIP8L2	**TNF alpha-induced protein 8-like protein 2**, acts as a negative regulator of innate and adaptive immunity by maintaining immune homeostasis, negatively regulates Toll-like receptor and T-cell receptor function. TNFAIP8L2 inhibits JUN/AP1 and NF-kappa-B activation, promotes Fas-induced apoptosis.	0.88
	SPI1	**Spi-1 proto-oncogene**, is a member of the Ets transcription factor family and plays a vital role in development and maturation of the myeloid and lymphoid lineages.	0.86
	WAS	As presented above.	0.81
	PECAM1	As presented above.	0.77
	Others	LAPTM5, CD37, GPSM3, ARHGAP9, TYROBP.	/

## Discussion

Our study found that, compared with in normal samples, GMFG was increased in six cancers (GBM, KIRC, LGG, LAML, PAAD, and TGCT), but was decreased in three cancers (KICH, LUAD, and LUSC). In addition, high expression of GMFG was associated with early pathological stage in four cancers (BLCA, LUAD, SKCM, and THCA), but was correlated with advanced pathological stage in STAD. Moreover, the associations of GMFG expression with OS and DFS were also investigated, and high expression of GMFG predicted worse OS in four cancers (GBM, LGG, LUSC, and UVM), and worse DFS for LGG and PRAD, but was associated with better OS in SKCM and THYM, better DFS in CHOL. These findings demonstrated significant differences in the expression patterns and prognostic values of GMFG in different cancers, indicating that GMFG may be a valuable biomarker for the diagnosis and prognosis of some cancers.

High expression of GMFG predicted different prognoses for different cancers. A possible explanation for this result is that GMFG functions differently in different cancers. First of all, combining the previous reports, we conclude that GMFG may enhance the motility of both cancer cells and immunocytes, because GMFG is mainly involved in protein binding in most cancers according to our study, which is consistent with the fact that GMFG binds to Arp2/3 complex to depolymerize the actin cytoskeleton [13]. In the literature, GMFG promotes the process of angiogenesis [7], and the proliferation and motility of cancer cell through altering the actin cytoskeleton [15, 16]. Thus, our research data suggest that GMFG plays an important role in tumor development largely through the protein-protein interaction. On the other hand, GMFG is also reported to enhance the chemotaxis of neutrophils and T lymphocytes through remodeling the actin cytoskeleton [11, 12]. In our study, GMFG is involved in immune response including T cell receptor signaling pathway, chemotaxis, T cell co-stimulation, leukocyte migration and T cell proliferation regulation, which may also be associated with the function of actin remodeling. Additionally, the biological process of immune response was also found in the KEGG pathways, such as Natural killer cell mediated cytotoxicity and B cell receptor signaling pathway. Taken together, that GMFG enhances both the cancer progression and the immune defense, and patients’ survival outcome may depend on the balance between the speed of cancer development and the ability of immune system against cancer tissues.

Another mechanism causing the different survival outcomes may be that GMFG appears strongly correlated with different immunomodulators including co-stimulators, co-inhibitors, ligands, receptors, cell adhesion and antigen presentation molecules in different cancers. Meanwhile, the strongest correlations between GMFG and immunomodulators were found in TGCT, SKCM, UVM, LUSC and COADREAD. However, GMFG was negatively correlated with most of the immunomodulators in THYM, and was weakly correlated with the immunomodulators of DLBC, LAML, and LIHC. These findings indicate that GMFG may participate in the regulation of cancer immunology. Recent studies reported that GMFG regulates monocyte migration by modulating ITGB1 [22], and functions as a negative regulator of Toll-like receptor 4 (TLR4) signaling through facilitating TLR4 endocytic trafficking in macrophages [23]. Interestingly, our study also found a strong correlation between GMFG and TLR4 as well as ITGB2. The significant associations between GMFG and immunomodulators show that GMFG modulates the migration, adhesion and activation of immunocytes through regulating immunomodulators, thus functions in the immune response of various cancers. Besides, GMFG is a cytokine-responsive protein mediating the pluripotential and lineage commitment of human hematopoietic stem cells [24], and is downregulated in the process of erythroid maturation and the response to LPS [25]. Apart from immune response, we show that GMFG also participates in cytokine-cytokine receptor interaction and hematopoietic cell lineage pathways, which is consistent with the previous studies. Furthermore, a study [26] reported that GMFG modulates the iron metabolism and M2 polarization of macrophage via inducing mitochondrial ROS and serves as a novel therapeutic target in immune and metabolic disorders. Therefore, GMFG may also act as a regulator of immunomodulators and a cytokine-responsive protein in modulating tumor immunity and hematopoiesis in addition to serving as an ADF-H protein in remodeling the actin cytoskeleton. GMFG may involve in the cancer immunity and hematopoiesis through interacting with different immunomodulators and cytokines, leading to different immune response status and tumor progression in different types of cancer.

Moreover, we found that GMFG had a significantly positive association with PD-1 and PD-L2, but not with PD-L1 in all cancers except for THYM, DLBC, LAML, and LIHC. Interestingly, the expression of GMFG was also obviously higher in THYM, DLBC and LAML than that in other cancer types, and GMFG was mainly enriched in the plasma membrane in most cancers, but was strongly concentrated on the nucleoplasm or extracellular exosome in THYM, DLBC and LAML. A previous bioinformatics study also demonstrated a weak correlation between PD-1 and other immunomodulators in THYM, LAML, and DLBC, while a strong correlation among them was observed in other types of cancer [27]. These results imply that GMFG has a weak interaction with immunomodulators and a different role in THYM, LAML and DLBC, and these immune and hematopoiesis system-related tumors may have a different immune tumor microenvironment (TME) compared to other solid cancers, which has been proved in recent studies [28–30]. PD-1 is a critical immune checkpoint in TME [31] which functions in adaptive resistance for cancer in immune escape [32]. PD-L1 and PD-L2 are important co-inhibitors for the immune inhibitory impact of PD-1 [33], PD-L1 is expressed in both tumor-infiltrating immune cells and tumor cells [34]. While it was initially suggested that PD-L2 expression is much more restricted than PD-L1 expression, and PD-L2 is expressed mainly in immune cells [35], but recent studies demonstrate that PD-L2 is also expressed in various types of tumor cells, depending on the different TME situations [36, 37]. What’s more, PD-L2 is also found to be significantly associated with the progression of some cancers [38, 39]. Thus, GMFG may interact with PD-1 and PD-L2 to promote the immune escape and progression of cancers.

Our study preliminary identified the potentially functional mechanisms of GMFG including protein binding, immune response and cytokine-cytokine receptor interaction. These findings may help to understand the real mechanisms of GMFG in regulating the tumor progression, immune response status and TME in different cancers. Therefore, in order to fully understand the role of GMFG, future cancer research on GMFG should not only focus on the function of GMFG in modulating of the actin cytoskeleton of cancer cell, cancer-associated vascular endothelial cells or immunocytes, but also pay attention to the cytokine-responsive function and immunomodulation impact of this factor, as well as its role in the tumor hematopoiesis and immune TME, especially its impacts on the motility and activation of the tumor-infiltrating immune cells, since the immune composition of the TME including the innate and adaptive immunocytes is a critical determinant of tumor development [40]. Meanwhile, the crosstalk between GMFG and immunomodulators should also be addressed in future studies. What’s more, our study also provides the overlapping co-expression genes in the cytokine-cytokine receptor interaction pathways, future studies may investigate the interaction between GMFG and these cytokines to better understand the role of GMFG in cancers. However, there are some limitations should be mentioned in this study. First, results of this study were mainly obtained by bioinformatics, the detailed mechanisms have not been verified by experiments. Thus, future experimental research should be performed to prove these pathways. Second, except for pathological stages, we failed to analyze the relationship between GMFG and other clinicopathological parameters of pan-cancers. In addition, this study mainly discussed GMFG-related KEGG pathways of cytokine-cytokine receptor interaction, the biological process of immune response, and the molecular function of protein binding, only limited attentions were paid to other GO/KEGG pathways such as cell adhesion molecules, signal transduction and receptor activity, which might also be critical. Finally, we mainly focused on mining and discussing the potential signaling of most cancers since they shared the same GO/KEGG pathways, we did not pay too much attention to the pathways of DLBC, LAML, and LIHC that were different from that of most cancer types.

## Conclusion

Our study demonstrates that the expression of GMFG varies in different cancers, and high expression of GMFG predicts different prognoses for different cancers. GMFG is mainly involved in immune response, protein binding and cytokine-cytokine receptor interaction pathways, and is positively associated with immunomodulators in most cancers. Overall, our findings may help to understand the real mechanisms of GMFG in regulating the tumor progression, immune response status and tissue-specific TME of different cancers.

## Supplementary Information

**Additional file 1.**

## Data Availability

The data of this study are from The Cancer Genome Atlas (https://portal.gdc.cancer.gov/), the GEPIA (http://gepia.cancer-pku.cn/), the MEM (http://biit.cs.ut.ee/mem), and cBioPortal (http://www.cbioportal.org) databases. Datasets generated for this study are included in the manuscript.

## Abbreviations

GMFG: Glia maturation factor γ
ADF-H: Actin-depolymerizing factor homology
Arps: Actin-related proteins
TCGA: The Cancer Genome Atlas
GO: Gene Ontology
KEGG: Kyoto Encyclopedia of Genes and Genomes
DAVID: Database for Annotation, Visualization and Integrated Discovery
GBM: Glioblastoma
KIRC: Kidney clear cell carcinoma
LGG: Lower grade glioma
LAML: Acute myeloid leukemia
PAAD: Pancreatic cancer
TGCT: Testicular cancer
KICH: Kidney chromophobe
LUAD: Lung adenocarcinoma
LUSC: Lung squamous cell carcinoma
OS: Overall survival
DFS: Disease-free survival
HR: Hazard ratio
PRAD: Prostate cancer
SKCM: Skin cutaneous melanoma
THYM: Thymoma
CHOL: Bile duct cancer
GEPIA: Gene Expression Profiling Interactive Analysis
GTEx: Genotype-Tissue Expression
MEM: Multi-Experiment Matrix
DLBC: Diffuse large B-cell lymphoma
MESO: Mesothelioma
PCPG: Pheochromocytoma and paraganglioma
SARC: Sarcoma
UVM: Uveal melanoma
BLCA: Bladder cancer
THCA: Thyroid cancer
STAD: Stage in stomach cancer
COADREAD: Colon and rectal cancer
PD-L2: PDCD1LG2
PD-1: PDCD1
LIHC: Liver cancer
TLR4: Toll-like receptor 4
TME: Tumor microenvironment
