# Supplementary material for: Bioinformatics and survival analysis of glia maturation factor-γ in pan-cancers
Source: BMC Cancer. 2021 Apr 17;21:423. doi: 10.1186/s12885-021-08163-2 (PMC8052856; doi:10.1186/s12885-021-08163-2)
Supplement: Supplementary file 1 — Additional file 1. [file 12885_2021_8163_MOESM1_ESM.docx]

**Title: Bioinformatics and survival analysis of glia maturation factor-γ in pan-cancers**

Aihua Lan ^1, *^, Chunxia Ren ^2,*^, Xiaoling Wang ^2,*^, Guoqing Tong ^2,#^, Gong Yang ^1,3,#^

1 Central Laboratory, the Fifth People's Hospital of Shanghai, Fudan University, Shanghai, 200240, China.

2 Center for Reproductive Medicine, Shuguang Hospital Affiliated to Shanghai University of Traditional Chinese Medicine, Shanghai, 200120, China.

3 Cancer Institute, Fudan University Shanghai Cancer Center, Department of Oncology, Fudan University Shanghai Medical College, Shanghai, 200032, China

***Contributed equally.**

**#Corresponding author:**

Guoqing Tong, Center for Reproductive Medicine, Shuguang Hospital Affiliated to Shanghai University of Traditional Chinese Medicine, Shanghai, 200120, China. drivftongguoqing@hotmail.com.

Gong Yang, Central Laboratory, the Fifth People's Hospital of Shanghai, Fudan University, Shanghai, 200240, China; Cancer Institute, Fudan University Shanghai Cancer Center, Department of Oncology, Fudan University Shanghai Medical College, Shanghai, 200240, China. Tel: 86-21-64175590-85201; Fax: 86-21-64172585;

E-mail: yanggong@fudan.edu.cn

**Table S1.** The top 10 most significant items of KEGG pathways of GMFG in 32 individual cancer types.

| Cancer type | Category | Term | Gene count | P-Value | FDR |
| --- | --- | --- | --- | --- | --- |
| LAML | KEGG_PATHWAY | hsa04144:Endocytosis | 14 | 6.78E-06 | 0.008267 |
|  | KEGG_PATHWAY | hsa05130:Pathogenic Escherichia coli infection | 7 | 3.44E-05 | 0.041938 |
|  | KEGG_PATHWAY | hsa05100:Bacterial invasion of epithelial cells | 8 | 4.46E-05 | 0.054356 |
|  | KEGG_PATHWAY | hsa04810:Regulation of actin cytoskeleton | 12 | 4.85E-05 | 0.059169 |
|  | KEGG_PATHWAY | hsa04670:Leukocyte transendothelial migration | 9 | 7.88E-05 | 0.09604 |
|  | KEGG_PATHWAY | hsa04611:Platelet activation | 9 | 1.86E-04 | 0.226853 |
|  | KEGG_PATHWAY | hsa00190:Oxidative phosphorylation | 9 | 2.18E-04 | 0.265524 |
|  | KEGG_PATHWAY | hsa05110:Vibrio cholerae infection | 6 | 4.21E-04 | 0.512914 |
|  | KEGG_PATHWAY | hsa04145:Phagosome | 9 | 4.93E-04 | 0.600235 |
|  | KEGG_PATHWAY | hsa05205:Proteoglycans in cancer | 10 | 7.55E-04 | 0.916811 |
|  |  |  |  |  |  |
| ACC | KEGG_PATHWAY | hsa04514:Cell adhesion molecules (CAMs) | 41 | 1.66E-19 | 2.11E-16 |
|  | KEGG_PATHWAY | hsa05150:Staphylococcus aureus infection | 25 | 2.62E-17 | 3.34E-14 |
|  | KEGG_PATHWAY | hsa04640:Hematopoietic cell lineage | 30 | 1.32E-16 | 1.44E-13 |
|  | KEGG_PATHWAY | hsa05416:Viral myocarditis | 23 | 2.25E-14 | 2.85E-11 |
|  | KEGG_PATHWAY | hsa05152:Tuberculosis | 39 | 2.93E-14 | 3.72E-11 |
|  | KEGG_PATHWAY | hsa04672:Intestinal immune network for IgA production | 21 | 3.77E-14 | 4.81E-11 |
|  | KEGG_PATHWAY | hsa05330:Allograft rejection | 19 | 4.06E-14 | 5.16E-11 |
|  | KEGG_PATHWAY | hsa05332:Graft-versus-host disease | 18 | 6.20E-14 | 7.89E-11 |
|  | KEGG_PATHWAY | hsa04380:Osteoclast differentiation | 33 | 7.93E-14 | 1.01E-10 |
|  | KEGG_PATHWAY | hsa04060:Cytokine-cytokine receptor interaction | 44 | 6.86E-13 | 8.73E-10 |
|  |  |  |  |  |  |
| CHOL | KEGG_PATHWAY | hsa04060:Cytokine-cytokine receptor interaction | 59 | 2.92E-22 | 3.75E-19 |
|  | KEGG_PATHWAY | hsa04514:Cell adhesion molecules (CAMs) | 43 | 4.47E-20 | 5.73E-17 |
|  | KEGG_PATHWAY | hsa04062:Chemokine signaling pathway | 47 | 1.90E-18 | 2.44E-15 |
|  | KEGG_PATHWAY | hsa04672:Intestinal immune network for IgA production | 25 | 2.48E-18 | 3.19E-15 |
|  | KEGG_PATHWAY | hsa05330:Allograft rejection | 21 | 4.13E-16 | 5.66E-13 |
|  | KEGG_PATHWAY | hsa04640:Hematopoietic cell lineage | 30 | 1.09E-15 | 1.42E-12 |
|  | KEGG_PATHWAY | hsa05332:Graft-versus-host disease | 19 | 1.08E-14 | 1.38E-11 |
|  | KEGG_PATHWAY | hsa04940:Type I diabetes mellitus | 20 | 1.65E-13 | 2.12E-10 |
|  | KEGG_PATHWAY | hsa05321:Inflammatory bowel disease (IBD) | 24 | 1.74E-13 | 2.23E-10 |
|  | KEGG_PATHWAY | hsa05340:Primary immunodeficiency | 18 | 4.05E-13 | 5.20E-10 |
|  |  |  |  |  |  |
| BLCA | KEGG_PATHWAY | hsa04640:Hematopoietic cell lineage | 9 | 7.86E-06 | 0.009157 |
|  | KEGG_PATHWAY | hsa05144:Malaria | 7 | 2.22E-05 | 0.025798 |
|  | KEGG_PATHWAY | hsa05340:Primary immunodeficiency | 6 | 4.52E-05 | 0.052571 |
|  | KEGG_PATHWAY | hsa04062:Chemokine signaling pathway | 11 | 6.48E-05 | 0.075399 |
|  | KEGG_PATHWAY | hsa04650:Natural killer cell mediated cytotoxicity | 9 | 9.25E-05 | 0.107721 |
|  | KEGG_PATHWAY | hsa04514:Cell adhesion molecules (CAMs) | 9 | 2.66E-04 | 0.309339 |
|  | KEGG_PATHWAY | hsa04670:Leukocyte transendothelial migration | 8 | 4.11E-04 | 0.477191 |
|  | KEGG_PATHWAY | hsa04611:Platelet activation | 8 | 8.58E-04 | 0.994668 |
|  | KEGG_PATHWAY | hsa04660:T cell receptor signaling pathway | 7 | 0.001165 | 1.348845 |
|  | KEGG_PATHWAY | hsa04380:Osteoclast differentiation | 7 | 0.004563 | 5.186509 |
|  |  |  |  |  |  |
| BRCA | KEGG_PATHWAY | hsa04060:Cytokine-cytokine receptor interaction | 59 | 1.62E-23 | 2.07E-20 |
|  | KEGG_PATHWAY | hsa05330:Allograft rejection | 24 | 8.07E-21 | 1.03E-17 |
|  | KEGG_PATHWAY | hsa04514:Cell adhesion molecules (CAMs) | 42 | 4.26E-20 | 5.45E-17 |
|  | KEGG_PATHWAY | hsa05150:Staphylococcus aureus infection | 26 | 2.60E-18 | 3.32E-15 |
|  | KEGG_PATHWAY | hsa05332:Graft-versus-host disease | 20 | 1.65E-16 | 1.44E-13 |
|  | KEGG_PATHWAY | hsa04940:Type I diabetes mellitus | 22 | 1.94E-16 | 2.89E-13 |
|  | KEGG_PATHWAY | hsa04672:Intestinal immune network for IgA production | 23 | 2.20E-16 | 2.89E-13 |
|  | KEGG_PATHWAY | hsa04640:Hematopoietic cell lineage | 29 | 2.29E-15 | 2.99E-12 |
|  | KEGG_PATHWAY | hsa05320:Autoimmune thyroid disease | 23 | 3.32E-15 | 4.26E-12 |
|  | KEGG_PATHWAY | hsa05340:Primary immunodeficiency | 19 | 8.17E-15 | 1.05E-11 |
|  |  |  |  |  |  |
| CESC | KEGG_PATHWAY | hsa04514:Cell adhesion molecules (CAMs) | 43 | 1.09E-23 | 1.38E-20 |
|  | KEGG_PATHWAY | hsa05150:Staphylococcus aureus infection | 27 | 2.84E-21 | 3.59E-18 |
|  | KEGG_PATHWAY | hsa04060:Cytokine-cytokine receptor interaction | 51 | 2.22E-20 | 2.80E-17 |
|  | KEGG_PATHWAY | hsa04640:Hematopoietic cell lineage | 31 | 2.68E-19 | 3.39E-16 |
|  | KEGG_PATHWAY | hsa05330:Allograft rejection | 21 | 6.45E-18 | 8.16E-15 |
|  | KEGG_PATHWAY | hsa04672:Intestinal immune network for IgA production | 23 | 7.80E-18 | 9.88E-15 |
|  | KEGG_PATHWAY | hsa04940:Type I diabetes mellitus | 20 | 3.45E-15 | 4.35E-12 |
|  | KEGG_PATHWAY | hsa05332:Graft-versus-host disease | 18 | 6.33E-15 | 8.00E-12 |
|  | KEGG_PATHWAY | hsa04062:Chemokine signaling pathway | 37 | 6.03E-14 | 7.64E-11 |
|  | KEGG_PATHWAY | hsa04660:T cell receptor signaling pathway | 27 | 1.65E-13 | 2.08E-10 |
|  |  |  |  |  |  |
| COADREAD | KEGG_PATHWAY | hsa04514:Cell adhesion molecules (CAMs) | 61 | 7.03E-27 | 9.13E-24 |
|  | KEGG_PATHWAY | hsa04060:Cytokine-cytokine receptor interaction | 76 | 3.26E-23 | 4.23E-20 |
|  | KEGG_PATHWAY | hsa05150:Staphylococcus aureus infection | 35 | 6.40E-23 | 8.31E-20 |
|  | KEGG_PATHWAY | hsa04640:Hematopoietic cell lineage | 42 | 7.46E-21 | 9.69E-18 |
|  | KEGG_PATHWAY | hsa04380:Osteoclast differentiation | 48 | 8.33E-18 | 1.08E-14 |
|  | KEGG_PATHWAY | hsa04062:Chemokine signaling pathway | 55 | 1.46E-15 | 1.88E-12 |
|  | KEGG_PATHWAY | hsa04672:Intestinal immune network for IgA production | 26 | 9.97E-15 | 1.30E-11 |
|  | KEGG_PATHWAY | hsa05323:Rheumatoid arthritis | 35 | 2.99E-14 | 3.88E-11 |
|  | KEGG_PATHWAY | hsa05144:Malaria | 26 | 3.58E-14 | 4.64E-11 |
|  | KEGG_PATHWAY | hsa05330:Allograft rejection | 22 | 2.56E-13 | 3.32E-10 |
|  |  |  |  |  |  |
| UCEC | KEGG_PATHWAY | hsa04060:Cytokine-cytokine receptor interaction | 39 | 4.82E-14 | 6.12E-11 |
|  | KEGG_PATHWAY | hsa04660:T cell receptor signaling pathway | 25 | 2.17E-13 | 2.76E-10 |
|  | KEGG_PATHWAY | hsa04514:Cell adhesion molecules (CAMs) | 29 | 3.90E-13 | 4.94E-10 |
|  | KEGG_PATHWAY | hsa05150:Staphylococcus aureus infection | 19 | 5.15E-13 | 6.53E-10 |
|  | KEGG_PATHWAY | hsa04380:Osteoclast differentiation | 27 | 2.42E-12 | 3.07E-09 |
|  | KEGG_PATHWAY | hsa04650:Natural killer cell mediated cytotoxicity | 26 | 3.09E-12 | 3.92E-09 |
|  | KEGG_PATHWAY | hsa04640:Hematopoietic cell lineage | 22 | 6.54E-12 | 8.30E-09 |
|  | KEGG_PATHWAY | hsa05340:Primary immunodeficiency | 13 | 2.06E-09 | 2.61E-06 |
|  | KEGG_PATHWAY | hsa04062:Chemokine signaling pathway | 27 | 7.58E-09 | 9.61E-06 |
|  | KEGG_PATHWAY | hsa05330:Allograft rejection | 12 | 7.61E-08 | 9.66E-05 |
|  |  |  |  |  |  |
| ESCA | KEGG_PATHWAY | hsa04514:Cell adhesion molecules (CAMs) | 47 | 6.10E-26 | 7.77E-23 |
|  | KEGG_PATHWAY | hsa05150:Staphylococcus aureus infection | 29 | 8.23E-23 | 1.05E-19 |
|  | KEGG_PATHWAY | hsa04640:Hematopoietic cell lineage | 31 | 4.27E-18 | 5.44E-15 |
|  | KEGG_PATHWAY | hsa04060:Cytokine-cytokine receptor interaction | 47 | 1.59E-15 | 1.98E-12 |
|  | KEGG_PATHWAY | hsa04672:Intestinal immune network for IgA production | 21 | 1.76E-14 | 2.25E-11 |
|  | KEGG_PATHWAY | hsa05330:Allograft rejection | 18 | 3.64E-13 | 4.64E-10 |
|  | KEGG_PATHWAY | hsa05332:Graft-versus-host disease | 17 | 6.38E-13 | 8.13E-10 |
|  | KEGG_PATHWAY | hsa05323:Rheumatoid arthritis | 25 | 3.87E-12 | 4.93E-09 |
|  | KEGG_PATHWAY | hsa04940:Type I diabetes mellitus | 18 | 4.62E-12 | 5.89E-09 |
|  | KEGG_PATHWAY | hsa04650:Natural killer cell mediated cytotoxicity | 29 | 5.96E-12 | 7.59E-09 |
|  |  |  |  |  |  |
| GBM | KEGG_PATHWAY | hsa05150:Staphylococcus aureus infection | 28 | 8.77E-16 | 1.17E-12 |
|  | KEGG_PATHWAY | hsa04060:Cytokine-cytokine receptor interaction | 59 | 4.42E-14 | 5.81E-11 |
|  | KEGG_PATHWAY | hsa05152:Tuberculosis | 44 | 5.51E-11 | 7.25E-08 |
|  | KEGG_PATHWAY | hsa05323:Rheumatoid arthritis | 29 | 1.80E-10 | 2.36E-07 |
|  | KEGG_PATHWAY | hsa04380:Osteoclast differentiation | 36 | 2.23E-10 | 2.94E-07 |
|  | KEGG_PATHWAY | hsa05140:Leishmaniasis | 25 | 9.14E-10 | 1.20E-06 |
|  | KEGG_PATHWAY | hsa05330:Allograft rejection | 18 | 1.10E-09 | 1.45E-06 |
|  | KEGG_PATHWAY | hsa05332:Graft-versus-host disease | 17 | 1.27E-09 | 1.67E-06 |
|  | KEGG_PATHWAY | hsa04672:Intestinal immune network for IgA production | 20 | 1.66E-09 | 2.18E-06 |
|  | KEGG_PATHWAY | hsa05321:Inflammatory bowel disease (IBD) | 23 | 3.23E-09 | 4.25E-06 |
|  |  |  |  |  |  |
| HNSC | KEGG_PATHWAY | hsa05150:Staphylococcus aureus infection | 26 | 1.24E-19 | 1.59E-16 |
|  | KEGG_PATHWAY | hsa04514:Cell adhesion molecules (CAMs) | 39 | 2.48E-19 | 3.15E-16 |
|  | KEGG_PATHWAY | hsa04060:Cytokine-cytokine receptor interaction | 49 | 3.11E-18 | 3.97E-15 |
|  | KEGG_PATHWAY | hsa04640:Hematopoietic cell lineage | 29 | 8.09E-17 | 1.44E-13 |
|  | KEGG_PATHWAY | hsa05330:Allograft rejection | 20 | 2.86E-16 | 4.22E-13 |
|  | KEGG_PATHWAY | hsa04672:Intestinal immune network for IgA production | 21 | 5.00E-15 | 6.36E-12 |
|  | KEGG_PATHWAY | hsa04940:Type I diabetes mellitus | 19 | 1.03E-13 | 1.32E-10 |
|  | KEGG_PATHWAY | hsa05332:Graft-versus-host disease | 17 | 2.30E-13 | 2.92E-10 |
|  | KEGG_PATHWAY | hsa05320:Autoimmune thyroid disease | 20 | 6.74E-13 | 8.58E-10 |
|  | KEGG_PATHWAY | hsa04062:Chemokine signaling pathway | 36 | 8.50E-13 | 1.08E-09 |
|  |  |  |  |  |  |
| KICH | KEGG_PATHWAY | hsa05150:Staphylococcus aureus infection | 33 | 3.16E-24 | 4.10E-21 |
|  | KEGG_PATHWAY | hsa04514:Cell adhesion molecules (CAMs) | 39 | 7.55E-14 | 9.80E-11 |
|  | KEGG_PATHWAY | hsa04060:Cytokine-cytokine receptor interaction | 51 | 5.83E-13 | 7.57E-10 |
|  | KEGG_PATHWAY | hsa04380:Osteoclast differentiation | 35 | 4.23E-12 | 5.49E-09 |
|  | KEGG_PATHWAY | hsa05332:Graft-versus-host disease | 18 | 4.72E-12 | 6.13E-09 |
|  | KEGG_PATHWAY | hsa04062:Chemokine signaling pathway | 42 | 8.02E-12 | 1.04E-08 |
|  | KEGG_PATHWAY | hsa04640:Hematopoietic cell lineage | 27 | 4.97E-11 | 6.45E-08 |
|  | KEGG_PATHWAY | hsa05330:Allograft rejection | 18 | 4.98E-11 | 6.46E-08 |
|  | KEGG_PATHWAY | hsa04145:Phagosome | 35 | 2.39E-10 | 3.10E-07 |
|  | KEGG_PATHWAY | hsa04650:Natural killer cell mediated cytotoxicity | 31 | 3.43E-10 | 4.45E-07 |
|  |  |  |  |  |  |
| KIRC | KEGG_PATHWAY | hsa04650:Natural killer cell mediated cytotoxicity | 14 | 1.45E-06 | 0.00178 |
|  | KEGG_PATHWAY | hsa05131:Shigellosis | 8 | 3.64E-04 | 0.446431 |
|  | KEGG_PATHWAY | hsa05340:Primary immunodeficiency | 6 | 6.60E-04 | 0.808544 |
|  | KEGG_PATHWAY | hsa04670:Leukocyte transendothelial migration | 10 | 6.75E-04 | 0.827283 |
|  | KEGG_PATHWAY | hsa04062:Chemokine signaling pathway | 12 | 0.00182 | 2.2145 |
|  | KEGG_PATHWAY | hsa04144:Endocytosis | 13 | 0.004744 | 5.678323 |
|  | KEGG_PATHWAY | hsa04060:Cytokine-cytokine receptor interaction | 13 | 0.005065 | 6.052189 |
|  | KEGG_PATHWAY | hsa05416:Viral myocarditis | 6 | 0.006744 | 7.983002 |
|  | KEGG_PATHWAY | hsa04666:Fc gamma R-mediated phagocytosis | 7 | 0.008348 | 9.793314 |
|  | KEGG_PATHWAY | hsa04640:Hematopoietic cell lineage | 7 | 0.009853 | 11.46245 |
|  |  |  |  |  |  |
| KIRP | KEGG_PATHWAY | hsa05150:Staphylococcus aureus infection | 29 | 8.84E-22 | 1.13E-18 |
|  | KEGG_PATHWAY | hsa04514:Cell adhesion molecules (CAMs) | 39 | 4.65E-17 | 5.97E-14 |
|  | KEGG_PATHWAY | hsa04060:Cytokine-cytokine receptor interaction | 50 | 4.31E-16 | 5.66E-13 |
|  | KEGG_PATHWAY | hsa04640:Hematopoietic cell lineage | 29 | 4.69E-15 | 5.98E-12 |
|  | KEGG_PATHWAY | hsa05330:Allograft rejection | 20 | 4.96E-15 | 6.42E-12 |
|  | KEGG_PATHWAY | hsa05152:Tuberculosis | 40 | 2.45E-14 | 3.15E-11 |
|  | KEGG_PATHWAY | hsa04062:Chemokine signaling pathway | 41 | 2.66E-14 | 3.41E-11 |
|  | KEGG_PATHWAY | hsa04672:Intestinal immune network for IgA production | 21 | 9.10E-14 | 1.17E-10 |
|  | KEGG_PATHWAY | hsa05332:Graft-versus-host disease | 18 | 1.33E-13 | 1.71E-10 |
|  | KEGG_PATHWAY | hsa05323:Rheumatoid arthritis | 27 | 4.43E-13 | 5.69E-10 |
|  |  |  |  |  |  |
| DLBC | KEGG_PATHWAY | hsa05016:Huntington's disease | 60 | 4.67E-20 | 6.13E-17 |
|  | KEGG_PATHWAY | hsa00190:Oxidative phosphorylation | 45 | 1.48E-16 | 1.44E-13 |
|  | KEGG_PATHWAY | hsa05010:Alzheimer's disease | 50 | 1.05E-15 | 1.31E-12 |
|  | KEGG_PATHWAY | hsa04932:Non-alcoholic fatty liver disease (NAFLD) | 47 | 1.27E-15 | 1.60E-12 |
|  | KEGG_PATHWAY | hsa05012:Parkinson's disease | 43 | 7.41E-14 | 9.71E-11 |
|  | KEGG_PATHWAY | hsa03010:Ribosome | 37 | 1.63E-10 | 2.14E-07 |
|  | KEGG_PATHWAY | hsa03050:Proteasome | 17 | 1.91E-07 | 2.50E-04 |
|  | KEGG_PATHWAY | hsa04141:Protein processing in endoplasmic reticulum | 32 | 1.83E-05 | 0.023984 |
|  | KEGG_PATHWAY | hsa01100:Metabolic pathways | 138 | 4.65E-05 | 0.060981 |
|  | KEGG_PATHWAY | hsa04919:Thyroid hormone signaling pathway | 24 | 5.51E-05 | 0.072261 |
|  |  |  |  |  |  |
| LIHC | KEGG_PATHWAY | hsa04060:Cytokine-cytokine receptor interaction | 44 | 4.33E-16 | 5.66E-13 |
|  | KEGG_PATHWAY | hsa05150:Staphylococcus aureus infection | 22 | 1.22E-15 | 1.54E-12 |
|  | KEGG_PATHWAY | hsa04514:Cell adhesion molecules (CAMs) | 31 | 1.26E-13 | 1.59E-10 |
|  | KEGG_PATHWAY | hsa04640:Hematopoietic cell lineage | 24 | 6.95E-13 | 8.81E-10 |
|  | KEGG_PATHWAY | hsa04672:Intestinal immune network for IgA production | 18 | 2.72E-12 | 3.45E-09 |
|  | KEGG_PATHWAY | hsa05330:Allograft rejection | 16 | 7.77E-12 | 9.83E-09 |
|  | KEGG_PATHWAY | hsa04650:Natural killer cell mediated cytotoxicity | 26 | 3.13E-11 | 3.96E-08 |
|  | KEGG_PATHWAY | hsa04062:Chemokine signaling pathway | 32 | 3.56E-11 | 4.51E-08 |
|  | KEGG_PATHWAY | hsa04660:T cell receptor signaling pathway | 23 | 1.16E-10 | 1.47E-07 |
|  | KEGG_PATHWAY | hsa05320:Autoimmune thyroid disease | 17 | 2.02E-10 | 2.56E-07 |
|  |  |  |  |  |  |
| LGG | KEGG_PATHWAY | hsa05150:Staphylococcus aureus infection | 31 | 1.00E-18 | 1.32E-15 |
|  | KEGG_PATHWAY | hsa05152:Tuberculosis | 54 | 7.13E-17 | 1.44E-13 |
|  | KEGG_PATHWAY | hsa04145:Phagosome | 46 | 1.95E-14 | 2.57E-11 |
|  | KEGG_PATHWAY | hsa05169:Epstein-Barr virus infection | 39 | 6.12E-13 | 8.07E-10 |
|  | KEGG_PATHWAY | hsa04612:Antigen processing and presentation | 28 | 4.91E-11 | 6.46E-08 |
|  | KEGG_PATHWAY | hsa05140:Leishmaniasis | 27 | 5.12E-11 | 6.75E-08 |
|  | KEGG_PATHWAY | hsa05416:Viral myocarditis | 24 | 6.76E-11 | 8.91E-08 |
|  | KEGG_PATHWAY | hsa04380:Osteoclast differentiation | 37 | 1.43E-10 | 1.88E-07 |
|  | KEGG_PATHWAY | hsa05330:Allograft rejection | 19 | 1.95E-10 | 2.57E-07 |
|  | KEGG_PATHWAY | hsa05145:Toxoplasmosis | 33 | 3.14E-10 | 4.13E-07 |
|  |  |  |  |  |  |
| LUAD | KEGG_PATHWAY | hsa04514:Cell adhesion molecules (CAMs) | 44 | 1.20E-23 | 1.52E-20 |
|  | KEGG_PATHWAY | hsa05330:Allograft rejection | 23 | 2.78E-20 | 3.52E-17 |
|  | KEGG_PATHWAY | hsa04672:Intestinal immune network for IgA production | 25 | 6.80E-20 | 8.62E-17 |
|  | KEGG_PATHWAY | hsa05150:Staphylococcus aureus infection | 26 | 2.44E-19 | 3.08E-16 |
|  | KEGG_PATHWAY | hsa04060:Cytokine-cytokine receptor interaction | 51 | 3.04E-19 | 3.85E-16 |
|  | KEGG_PATHWAY | hsa04640:Hematopoietic cell lineage | 30 | 1.59E-17 | 2.01E-14 |
|  | KEGG_PATHWAY | hsa05320:Autoimmune thyroid disease | 24 | 2.48E-17 | 3.15E-14 |
|  | KEGG_PATHWAY | hsa05332:Graft-versus-host disease | 20 | 2.66E-17 | 3.37E-14 |
|  | KEGG_PATHWAY | hsa04940:Type I diabetes mellitus | 21 | 5.27E-16 | 6.99E-13 |
|  | KEGG_PATHWAY | hsa05416:Viral myocarditis | 23 | 4.41E-15 | 5.63E-12 |
|  |  |  |  |  |  |
| LUSC | KEGG_PATHWAY | hsa05150:Staphylococcus aureus infection | 34 | 1.82E-24 | 2.36E-21 |
|  | KEGG_PATHWAY | hsa04060:Cytokine-cytokine receptor interaction | 68 | 1.02E-22 | 1.33E-19 |
|  | KEGG_PATHWAY | hsa04514:Cell adhesion molecules (CAMs) | 51 | 2.71E-22 | 3.52E-19 |
|  | KEGG_PATHWAY | hsa05330:Allograft rejection | 26 | 2.15E-20 | 2.79E-17 |
|  | KEGG_PATHWAY | hsa04640:Hematopoietic cell lineage | 38 | 5.07E-20 | 6.59E-17 |
|  | KEGG_PATHWAY | hsa04940:Type I diabetes mellitus | 26 | 2.05E-18 | 2.66E-15 |
|  | KEGG_PATHWAY | hsa05332:Graft-versus-host disease | 23 | 6.83E-18 | 8.88E-15 |
|  | KEGG_PATHWAY | hsa04672:Intestinal immune network for IgA production | 26 | 8.28E-17 | 1.44E-13 |
|  | KEGG_PATHWAY | hsa05323:Rheumatoid arthritis | 34 | 6.16E-16 | 8.66E-13 |
|  | KEGG_PATHWAY | hsa05320:Autoimmune thyroid disease | 26 | 1.96E-15 | 2.60E-12 |
|  |  |  |  |  |  |
| SKCM | KEGG_PATHWAY | hsa04060:Cytokine-cytokine receptor interaction | 68 | 6.79E-27 | 8.75E-24 |
|  | KEGG_PATHWAY | hsa05150:Staphylococcus aureus infection | 32 | 4.88E-24 | 6.29E-21 |
|  | KEGG_PATHWAY | hsa05330:Allograft rejection | 25 | 1.17E-20 | 1.50E-17 |
|  | KEGG_PATHWAY | hsa04640:Hematopoietic cell lineage | 35 | 2.47E-19 | 3.19E-16 |
|  | KEGG_PATHWAY | hsa04514:Cell adhesion molecules (CAMs) | 44 | 3.41E-19 | 4.40E-16 |
|  | KEGG_PATHWAY | hsa04940:Type I diabetes mellitus | 25 | 8.52E-19 | 1.10E-15 |
|  | KEGG_PATHWAY | hsa04672:Intestinal immune network for IgA production | 26 | 1.67E-18 | 2.15E-15 |
|  | KEGG_PATHWAY | hsa05332:Graft-versus-host disease | 22 | 5.79E-18 | 7.46E-15 |
|  | KEGG_PATHWAY | hsa05321:Inflammatory bowel disease (IBD) | 28 | 1.34E-16 | 1.44E-13 |
|  | KEGG_PATHWAY | hsa04380:Osteoclast differentiation | 39 | 2.18E-16 | 2.89E-13 |
|  |  |  |  |  |  |
| MESO | KEGG_PATHWAY | hsa04514:Cell adhesion molecules (CAMs) | 35 | 2.77E-17 | 3.54E-14 |
|  | KEGG_PATHWAY | hsa05330:Allograft rejection | 19 | 8.64E-16 | 1.13E-12 |
|  | KEGG_PATHWAY | hsa05332:Graft-versus-host disease | 18 | 1.73E-15 | 2.26E-12 |
|  | KEGG_PATHWAY | hsa05150:Staphylococcus aureus infection | 21 | 1.45E-14 | 1.86E-11 |
|  | KEGG_PATHWAY | hsa04940:Type I diabetes mellitus | 19 | 1.49E-14 | 1.90E-11 |
|  | KEGG_PATHWAY | hsa05140:Leishmaniasis | 21 | 5.30E-12 | 6.76E-09 |
|  | KEGG_PATHWAY | hsa05321:Inflammatory bowel disease (IBD) | 20 | 6.52E-12 | 8.31E-09 |
|  | KEGG_PATHWAY | hsa05416:Viral myocarditis | 19 | 7.36E-12 | 9.38E-09 |
|  | KEGG_PATHWAY | hsa05320:Autoimmune thyroid disease | 18 | 1.52E-11 | 1.94E-08 |
|  | KEGG_PATHWAY | hsa05152:Tuberculosis | 31 | 3.76E-11 | 4.80E-08 |
|  |  |  |  |  |  |
| UVM | KEGG_PATHWAY | hsa05332:Graft-versus-host disease | 26 | 1.77E-22 | 2.31E-19 |
|  | KEGG_PATHWAY | hsa05330:Allograft rejection | 26 | 1.77E-20 | 2.31E-17 |
|  | KEGG_PATHWAY | hsa04060:Cytokine-cytokine receptor interaction | 64 | 4.67E-20 | 6.11E-17 |
|  | KEGG_PATHWAY | hsa04940:Type I diabetes mellitus | 27 | 8.31E-20 | 1.09E-16 |
|  | KEGG_PATHWAY | hsa04640:Hematopoietic cell lineage | 37 | 3.80E-19 | 4.96E-16 |
|  | KEGG_PATHWAY | hsa05150:Staphylococcus aureus infection | 29 | 2.21E-18 | 2.90E-15 |
|  | KEGG_PATHWAY | hsa04514:Cell adhesion molecules (CAMs) | 46 | 3.09E-18 | 4.04E-15 |
|  | KEGG_PATHWAY | hsa04612:Antigen processing and presentation | 33 | 2.03E-17 | 2.66E-14 |
|  | KEGG_PATHWAY | hsa05323:Rheumatoid arthritis | 34 | 3.99E-16 | 5.77E-13 |
|  | KEGG_PATHWAY | hsa05320:Autoimmune thyroid disease | 25 | 1.98E-14 | 2.60E-11 |
|  |  |  |  |  |  |
| OV | KEGG_PATHWAY | hsa04060:Cytokine-cytokine receptor interaction | 56 | 1.86E-24 | 2.34E-21 |
|  | KEGG_PATHWAY | hsa05150:Staphylococcus aureus infection | 28 | 1.41E-22 | 1.78E-19 |
|  | KEGG_PATHWAY | hsa04640:Hematopoietic cell lineage | 31 | 2.93E-19 | 3.69E-16 |
|  | KEGG_PATHWAY | hsa04514:Cell adhesion molecules (CAMs) | 38 | 7.71E-19 | 9.70E-16 |
|  | KEGG_PATHWAY | hsa05330:Allograft rejection | 21 | 6.87E-18 | 8.64E-15 |
|  | KEGG_PATHWAY | hsa05332:Graft-versus-host disease | 20 | 9.33E-18 | 1.17E-14 |
|  | KEGG_PATHWAY | hsa04940:Type I diabetes mellitus | 21 | 1.78E-16 | 2.78E-13 |
|  | KEGG_PATHWAY | hsa05323:Rheumatoid arthritis | 28 | 6.71E-16 | 8.44E-13 |
|  | KEGG_PATHWAY | hsa05321:Inflammatory bowel disease (IBD) | 24 | 1.87E-15 | 2.38E-12 |
|  | KEGG_PATHWAY | hsa04380:Osteoclast differentiation | 32 | 1.28E-14 | 1.61E-11 |
|  |  |  |  |  |  |
| PAAD | KEGG_PATHWAY | hsa04640:Hematopoietic cell lineage | 40 | 1.61E-23 | 2.08E-20 |
|  | KEGG_PATHWAY | hsa05150:Staphylococcus aureus infection | 32 | 4.33E-23 | 5.58E-20 |
|  | KEGG_PATHWAY | hsa04514:Cell adhesion molecules (CAMs) | 45 | 7.89E-19 | 1.02E-15 |
|  | KEGG_PATHWAY | hsa04060:Cytokine-cytokine receptor interaction | 58 | 9.54E-18 | 1.23E-14 |
|  | KEGG_PATHWAY | hsa04380:Osteoclast differentiation | 41 | 5.79E-17 | 1.44E-13 |
|  | KEGG_PATHWAY | hsa04672:Intestinal immune network for IgA production | 25 | 1.49E-16 | 1.44E-13 |
|  | KEGG_PATHWAY | hsa04062:Chemokine signaling pathway | 47 | 2.43E-15 | 3.14E-12 |
|  | KEGG_PATHWAY | hsa04660:T cell receptor signaling pathway | 31 | 9.92E-13 | 1.28E-09 |
|  | KEGG_PATHWAY | hsa05323:Rheumatoid arthritis | 29 | 1.15E-12 | 1.48E-09 |
|  | KEGG_PATHWAY | hsa05330:Allograft rejection | 18 | 4.06E-11 | 5.22E-08 |
|  |  |  |  |  |  |
| PCPG | KEGG_PATHWAY | hsa04510:Focal adhesion | 40 | 1.79E-08 | 2.34E-05 |
|  | KEGG_PATHWAY | hsa04670:Leukocyte transendothelial migration | 27 | 1.23E-07 | 1.61E-04 |
|  | KEGG_PATHWAY | hsa04611:Platelet activation | 27 | 1.55E-06 | 0.002028 |
|  | KEGG_PATHWAY | hsa04668:TNF signaling pathway | 22 | 2.13E-05 | 0.027934 |
|  | KEGG_PATHWAY | hsa04610:Complement and coagulation cascades | 17 | 2.41E-05 | 0.031532 |
|  | KEGG_PATHWAY | hsa05200:Pathways in cancer | 51 | 5.16E-05 | 0.067593 |
|  | KEGG_PATHWAY | hsa04015:Rap1 signaling pathway | 31 | 2.38E-04 | 0.311463 |
|  | KEGG_PATHWAY | hsa04666:Fc gamma R-mediated phagocytosis | 17 | 2.91E-04 | 0.380241 |
|  | KEGG_PATHWAY | hsa04380:Osteoclast differentiation | 22 | 4.36E-04 | 0.568965 |
|  | KEGG_PATHWAY | hsa04010:MAPK signaling pathway | 34 | 6.44E-04 | 0.840572 |
|  |  |  |  |  |  |
| PRAD | KEGG_PATHWAY | hsa05150:Staphylococcus aureus infection | 26 | 1.53E-17 | 1.96E-14 |
|  | KEGG_PATHWAY | hsa04060:Cytokine-cytokine receptor interaction | 53 | 1.90E-17 | 2.44E-14 |
|  | KEGG_PATHWAY | hsa04514:Cell adhesion molecules (CAMs) | 40 | 3.34E-17 | 4.28E-14 |
|  | KEGG_PATHWAY | hsa04672:Intestinal immune network for IgA production | 23 | 1.15E-15 | 1.42E-12 |
|  | KEGG_PATHWAY | hsa04640:Hematopoietic cell lineage | 30 | 1.80E-15 | 2.28E-12 |
|  | KEGG_PATHWAY | hsa04062:Chemokine signaling pathway | 41 | 1.28E-13 | 1.63E-10 |
|  | KEGG_PATHWAY | hsa05330:Allograft rejection | 17 | 4.39E-11 | 5.62E-08 |
|  | KEGG_PATHWAY | hsa05152:Tuberculosis | 36 | 5.91E-11 | 7.57E-08 |
|  | KEGG_PATHWAY | hsa05340:Primary immunodeficiency | 16 | 1.27E-10 | 1.63E-07 |
|  | KEGG_PATHWAY | hsa05416:Viral myocarditis | 20 | 1.46E-10 | 1.87E-07 |
|  |  |  |  |  |  |
| SARC | KEGG_PATHWAY | hsa05150:Staphylococcus aureus infection | 33 | 4.24E-21 | 5.58E-18 |
|  | KEGG_PATHWAY | hsa04060:Cytokine-cytokine receptor interaction | 71 | 5.53E-21 | 7.28E-18 |
|  | KEGG_PATHWAY | hsa05152:Tuberculosis | 56 | 2.58E-18 | 3.40E-15 |
|  | KEGG_PATHWAY | hsa05330:Allograft rejection | 25 | 1.96E-17 | 2.58E-14 |
|  | KEGG_PATHWAY | hsa05323:Rheumatoid arthritis | 37 | 1.17E-16 | 1.44E-13 |
|  | KEGG_PATHWAY | hsa05332:Graft-versus-host disease | 23 | 1.84E-16 | 2.89E-13 |
|  | KEGG_PATHWAY | hsa04514:Cell adhesion molecules (CAMs) | 46 | 1.86E-15 | 2.49E-12 |
|  | KEGG_PATHWAY | hsa04380:Osteoclast differentiation | 44 | 1.94E-15 | 2.49E-12 |
|  | KEGG_PATHWAY | hsa04672:Intestinal immune network for IgA production | 26 | 3.34E-15 | 4.37E-12 |
|  | KEGG_PATHWAY | hsa04940:Type I diabetes mellitus | 24 | 1.90E-14 | 2.50E-11 |
|  |  |  |  |  |  |
| STAD | KEGG_PATHWAY | hsa05150:Staphylococcus aureus infection | 32 | 1.00E-22 | 1.30E-19 |
|  | KEGG_PATHWAY | hsa04640:Hematopoietic cell lineage | 36 | 5.98E-19 | 7.74E-16 |
|  | KEGG_PATHWAY | hsa04514:Cell adhesion molecules (CAMs) | 43 | 9.37E-17 | 1.44E-13 |
|  | KEGG_PATHWAY | hsa04060:Cytokine-cytokine receptor interaction | 52 | 2.66E-13 | 3.45E-10 |
|  | KEGG_PATHWAY | hsa04380:Osteoclast differentiation | 36 | 1.24E-12 | 1.60E-09 |
|  | KEGG_PATHWAY | hsa04062:Chemokine signaling pathway | 42 | 1.30E-11 | 1.68E-08 |
|  | KEGG_PATHWAY | hsa05144:Malaria | 20 | 1.73E-10 | 2.24E-07 |
|  | KEGG_PATHWAY | hsa04670:Leukocyte transendothelial migration | 30 | 5.08E-10 | 6.57E-07 |
|  | KEGG_PATHWAY | hsa04672:Intestinal immune network for IgA production | 19 | 6.72E-10 | 8.70E-07 |
|  | KEGG_PATHWAY | hsa05321:Inflammatory bowel disease (IBD) | 22 | 7.13E-10 | 9.23E-07 |
|  |  |  |  |  |  |
| TGCT | KEGG_PATHWAY | hsa04060:Cytokine-cytokine receptor interaction | 69 | 1.80E-22 | 2.34E-19 |
|  | KEGG_PATHWAY | hsa05330:Allograft rejection | 26 | 5.57E-20 | 7.25E-17 |
|  | KEGG_PATHWAY | hsa05152:Tuberculosis | 54 | 4.00E-19 | 5.21E-16 |
|  | KEGG_PATHWAY | hsa04514:Cell adhesion molecules (CAMs) | 48 | 4.88E-19 | 6.34E-16 |
|  | KEGG_PATHWAY | hsa05332:Graft-versus-host disease | 24 | 5.99E-19 | 7.79E-16 |
|  | KEGG_PATHWAY | hsa04640:Hematopoietic cell lineage | 37 | 1.78E-18 | 2.31E-15 |
|  | KEGG_PATHWAY | hsa05140:Leishmaniasis | 33 | 6.86E-18 | 8.93E-15 |
|  | KEGG_PATHWAY | hsa04612:Antigen processing and presentation | 34 | 8.37E-18 | 1.09E-14 |
|  | KEGG_PATHWAY | hsa04940:Type I diabetes mellitus | 25 | 9.15E-17 | 1.44E-13 |
|  | KEGG_PATHWAY | hsa04380:Osteoclast differentiation | 43 | 1.46E-16 | 1.44E-13 |
|  |  |  |  |  |  |
| THYM | KEGG_PATHWAY | hsa03010:Ribosome | 60 | 5.79E-12 | 7.66E-09 |
|  | KEGG_PATHWAY | hsa05010:Alzheimer's disease | 67 | 6.90E-11 | 9.13E-08 |
|  | KEGG_PATHWAY | hsa03040:Spliceosome | 56 | 2.85E-10 | 3.77E-07 |
|  | KEGG_PATHWAY | hsa04932:Non-alcoholic fatty liver disease (NAFLD) | 55 | 1.65E-07 | 2.19E-04 |
|  | KEGG_PATHWAY | hsa05012:Parkinson's disease | 51 | 8.14E-07 | 0.001078 |
|  | KEGG_PATHWAY | hsa05016:Huntington's disease | 61 | 6.28E-06 | 0.008308 |
|  | KEGG_PATHWAY | hsa03030:DNA replication | 19 | 1.37E-05 | 0.01815 |
|  | KEGG_PATHWAY | hsa04660:T cell receptor signaling pathway | 37 | 1.60E-05 | 0.021189 |
|  | KEGG_PATHWAY | hsa00190:Oxidative phosphorylation | 44 | 5.25E-05 | 0.069515 |
|  | KEGG_PATHWAY | hsa05162:Measles | 42 | 2.51E-04 | 0.331642 |
|  |  |  |  |  |  |
| THCA | KEGG_PATHWAY | hsa04650:Natural killer cell mediated cytotoxicity | 24 | 2.85E-09 | 3.66E-06 |
|  | KEGG_PATHWAY | hsa04611:Platelet activation | 21 | 1.02E-06 | 0.001304 |
|  | KEGG_PATHWAY | hsa05340:Primary immunodeficiency | 11 | 1.37E-06 | 0.001751 |
|  | KEGG_PATHWAY | hsa04660:T cell receptor signaling pathway | 18 | 1.63E-06 | 0.002085 |
|  | KEGG_PATHWAY | hsa04060:Cytokine-cytokine receptor interaction | 29 | 3.11E-06 | 0.003993 |
|  | KEGG_PATHWAY | hsa04380:Osteoclast differentiation | 20 | 4.70E-06 | 0.00603 |
|  | KEGG_PATHWAY | hsa04062:Chemokine signaling pathway | 24 | 7.60E-06 | 0.009737 |
|  | KEGG_PATHWAY | hsa03050:Proteasome | 11 | 1.74E-05 | 0.022252 |
|  | KEGG_PATHWAY | hsa04640:Hematopoietic cell lineage | 15 | 2.62E-05 | 0.033525 |
|  | KEGG_PATHWAY | hsa04670:Leukocyte transendothelial migration | 17 | 4.53E-05 | 0.058056 |
|  |  |  |  |  |  |
| UCS | KEGG_PATHWAY | hsa05150:Staphylococcus aureus infection | 19 | 1.55E-14 | 1.98E-11 |
|  | KEGG_PATHWAY | hsa04145:Phagosome | 24 | 1.85E-10 | 2.35E-07 |
|  | KEGG_PATHWAY | hsa04514:Cell adhesion molecules (CAMs) | 23 | 3.84E-10 | 4.89E-07 |
|  | KEGG_PATHWAY | hsa04380:Osteoclast differentiation | 22 | 5.14E-10 | 6.55E-07 |
|  | KEGG_PATHWAY | hsa05152:Tuberculosis | 24 | 5.23E-09 | 6.67E-06 |
|  | KEGG_PATHWAY | hsa04640:Hematopoietic cell lineage | 17 | 8.44E-09 | 1.07E-05 |
|  | KEGG_PATHWAY | hsa05330:Allograft rejection | 11 | 1.25E-07 | 1.60E-04 |
|  | KEGG_PATHWAY | hsa05416:Viral myocarditis | 13 | 1.38E-07 | 1.75E-04 |
|  | KEGG_PATHWAY | hsa04062:Chemokine signaling pathway | 22 | 2.96E-07 | 3.77E-04 |
|  | KEGG_PATHWAY | hsa04060:Cytokine-cytokine receptor interaction | 25 | 5.02E-07 | 6.40E-04 |

**Table S2.** The top 10 most significant items of biological processes of GMFG in 32 individual cancer types.

| Cancer type | Category | Term | Gene count | P-Value | FDR |
| --- | --- | --- | --- | --- | --- |
| LAML | GOTERM_BP_DIRECT | GO:0008286~insulin receptor signaling pathway | 8 | 2.95E-05 | 0.047093 |
|  | GOTERM_BP_DIRECT | GO:0048013~ephrin receptor signaling pathway | 7 | 4.43E-04 | 0.704806 |
|  | GOTERM_BP_DIRECT | GO:0006928~movement of cell or subcellular component | 7 | 4.43E-04 | 0.704806 |
|  | GOTERM_BP_DIRECT | GO:0007035~vacuolar acidification | 4 | 4.81E-04 | 0.765271 |
|  | GOTERM_BP_DIRECT | GO:0016241~regulation of macroautophagy | 5 | 0.001543 | 2.436982 |
|  | GOTERM_BP_DIRECT | GO:0007264~small GTPase mediated signal transduction | 10 | 0.002063 | 3.245437 |
|  | GOTERM_BP_DIRECT | GO:0015992~proton transport | 5 | 0.002305 | 3.618822 |
|  | GOTERM_BP_DIRECT | GO:0021762~substantia nigra development | 5 | 0.002305 | 3.618822 |
|  | GOTERM_BP_DIRECT | GO:0051099~positive regulation of binding | 3 | 0.002603 | 4.078472 |
|  | GOTERM_BP_DIRECT | GO:0090383~phagosome acidification | 4 | 0.003466 | 5.394877 |
|  |  |  |  |  |  |
| ACC | GOTERM_BP_DIRECT | GO:0006955~immune response | 93 | 5.46E-42 | 9.76E-39 |
|  | GOTERM_BP_DIRECT | GO:0006954~inflammatory response | 74 | 1.30E-29 | 2.32E-26 |
|  | GOTERM_BP_DIRECT | GO:0002250~adaptive immune response | 45 | 2.37E-26 | 4.24E-23 |
|  | GOTERM_BP_DIRECT | GO:0050776~regulation of immune response | 48 | 1.41E-25 | 2.52E-22 |
|  | GOTERM_BP_DIRECT | GO:0045087~innate immune response | 73 | 2.56E-25 | 4.57E-22 |
|  | GOTERM_BP_DIRECT | GO:0031295~T cell costimulation | 30 | 7.23E-21 | 1.29E-17 |
|  | GOTERM_BP_DIRECT | GO:0007165~signal transduction | 117 | 2.71E-20 | 4.85E-17 |
|  | GOTERM_BP_DIRECT | GO:0050852~T cell receptor signaling pathway | 36 | 1.05E-17 | 1.87E-14 |
|  | GOTERM_BP_DIRECT | GO:0050900~leukocyte migration | 30 | 6.52E-15 | 1.17E-11 |
|  | GOTERM_BP_DIRECT | GO:0042102~positive regulation of T cell proliferation | 22 | 7.81E-15 | 1.39E-11 |
|  |  |  |  |  |  |
| CHOL | GOTERM_BP_DIRECT | GO:0006955~immune response | 112 | 3.31E-53 | 5.98E-50 |
|  | GOTERM_BP_DIRECT | GO:0006954~inflammatory response | 93 | 8.61E-41 | 1.56E-37 |
|  | GOTERM_BP_DIRECT | GO:0002250~adaptive immune response | 54 | 4.94E-33 | 8.92E-30 |
|  | GOTERM_BP_DIRECT | GO:0050776~regulation of immune response | 53 | 1.56E-27 | 2.81E-24 |
|  | GOTERM_BP_DIRECT | GO:0042102~positive regulation of T cell proliferation | 31 | 2.23E-24 | 4.03E-21 |
|  | GOTERM_BP_DIRECT | GO:0045087~innate immune response | 72 | 5.48E-21 | 9.90E-18 |
|  | GOTERM_BP_DIRECT | GO:0031295~T cell costimulation | 30 | 3.64E-19 | 6.58E-16 |
|  | GOTERM_BP_DIRECT | GO:0042110~T cell activation | 22 | 3.44E-16 | 6.00E-13 |
|  | GOTERM_BP_DIRECT | GO:0070098~chemokine-mediated signaling pathway | 26 | 4.65E-16 | 7.99E-13 |
|  | GOTERM_BP_DIRECT | GO:0007165~signal transduction | 119 | 4.88E-16 | 7.99E-13 |
|  |  |  |  |  |  |
| BLCA | GOTERM_BP_DIRECT | GO:0050776~regulation of immune response | 16 | 2.63E-10 | 4.12E-07 |
|  | GOTERM_BP_DIRECT | GO:0006955~immune response | 22 | 1.14E-09 | 1.79E-06 |
|  | GOTERM_BP_DIRECT | GO:0002250~adaptive immune response | 12 | 2.51E-07 | 3.94E-04 |
|  | GOTERM_BP_DIRECT | GO:0007155~cell adhesion | 19 | 6.92E-07 | 0.001085 |
|  | GOTERM_BP_DIRECT | GO:0045087~innate immune response | 18 | 1.29E-06 | 0.002018 |
|  | GOTERM_BP_DIRECT | GO:0042102~positive regulation of T cell proliferation | 8 | 2.02E-06 | 0.003167 |
|  | GOTERM_BP_DIRECT | GO:0007165~signal transduction | 29 | 9.53E-06 | 0.014938 |
|  | GOTERM_BP_DIRECT | GO:0006954~inflammatory response | 15 | 2.48E-05 | 0.038779 |
|  | GOTERM_BP_DIRECT | GO:0007169~transmembrane receptor protein tyrosine kinase signaling pathway | 8 | 4.63E-05 | 0.072537 |
|  | GOTERM_BP_DIRECT | GO:0042110~T cell activation | 6 | 9.75E-05 | 0.152617 |
|  |  |  |  |  |  |
| BRCA | GOTERM_BP_DIRECT | GO:0006955~immune response | 115 | 2.93E-63 | 5.22E-60 |
|  | GOTERM_BP_DIRECT | GO:0006954~inflammatory response | 92 | 1.75E-45 | 3.11E-42 |
|  | GOTERM_BP_DIRECT | GO:0002250~adaptive immune response | 55 | 1.51E-37 | 2.68E-34 |
|  | GOTERM_BP_DIRECT | GO:0050776~regulation of immune response | 59 | 4.21E-37 | 7.50E-34 |
|  | GOTERM_BP_DIRECT | GO:0045087~innate immune response | 82 | 2.27E-32 | 4.04E-29 |
|  | GOTERM_BP_DIRECT | GO:0031295~T cell costimulation | 37 | 9.56E-30 | 1.70E-26 |
|  | GOTERM_BP_DIRECT | GO:0042102~positive regulation of T cell proliferation | 29 | 1.41E-23 | 2.51E-20 |
|  | GOTERM_BP_DIRECT | GO:0007165~signal transduction | 114 | 3.38E-19 | 6.02E-16 |
|  | GOTERM_BP_DIRECT | GO:0050852~T cell receptor signaling pathway | 37 | 9.97E-19 | 1.77E-15 |
|  | GOTERM_BP_DIRECT | GO:0060333~interferon-gamma-mediated signaling pathway | 25 | 1.75E-16 | 4.00E-13 |
|  |  |  |  |  |  |
| CESC | GOTERM_BP_DIRECT | GO:0006955~immune response | 99 | 9.68E-56 | 1.68E-52 |
|  | GOTERM_BP_DIRECT | GO:0002250~adaptive immune response | 51 | 4.42E-37 | 7.67E-34 |
|  | GOTERM_BP_DIRECT | GO:0050776~regulation of immune response | 52 | 8.22E-34 | 1.43E-30 |
|  | GOTERM_BP_DIRECT | GO:0006954~inflammatory response | 72 | 1.09E-33 | 1.90E-30 |
|  | GOTERM_BP_DIRECT | GO:0031295~T cell costimulation | 33 | 3.09E-27 | 5.37E-24 |
|  | GOTERM_BP_DIRECT | GO:0045087~innate immune response | 64 | 7.85E-24 | 1.36E-20 |
|  | GOTERM_BP_DIRECT | GO:0042102~positive regulation of T cell proliferation | 26 | 9.46E-22 | 1.64E-18 |
|  | GOTERM_BP_DIRECT | GO:0050852~T cell receptor signaling pathway | 35 | 1.52E-19 | 2.64E-16 |
|  | GOTERM_BP_DIRECT | GO:0007165~signal transduction | 96 | 6.09E-17 | 1.89E-13 |
|  | GOTERM_BP_DIRECT | GO:0006935~chemotaxis | 29 | 2.59E-16 | 3.89E-13 |
|  |  |  |  |  |  |
| COADREAD | GOTERM_BP_DIRECT | GO:0006954~inflammatory response | 129 | 5.01E-53 | 9.36E-50 |
|  | GOTERM_BP_DIRECT | GO:0006955~immune response | 134 | 2.96E-51 | 5.53E-48 |
|  | GOTERM_BP_DIRECT | GO:0007155~cell adhesion | 119 | 1.29E-35 | 2.40E-32 |
|  | GOTERM_BP_DIRECT | GO:0002250~adaptive immune response | 57 | 1.57E-26 | 2.94E-23 |
|  | GOTERM_BP_DIRECT | GO:0007165~signal transduction | 185 | 1.27E-25 | 2.37E-22 |
|  | GOTERM_BP_DIRECT | GO:0050776~regulation of immune response | 60 | 2.11E-24 | 3.95E-21 |
|  | GOTERM_BP_DIRECT | GO:0030198~extracellular matrix organization | 63 | 2.42E-24 | 4.53E-21 |
|  | GOTERM_BP_DIRECT | GO:0050900~leukocyte migration | 47 | 5.92E-22 | 1.11E-18 |
|  | GOTERM_BP_DIRECT | GO:0001525~angiogenesis | 63 | 4.47E-21 | 8.36E-18 |
|  | GOTERM_BP_DIRECT | GO:0006935~chemotaxis | 46 | 4.69E-21 | 8.76E-18 |
|  |  |  |  |  |  |
| UCEC | GOTERM_BP_DIRECT | GO:0006955~immune response | 75 | 5.19E-37 | 8.93E-34 |
|  | GOTERM_BP_DIRECT | GO:0006954~inflammatory response | 63 | 3.27E-29 | 5.62E-26 |
|  | GOTERM_BP_DIRECT | GO:0002250~adaptive immune response | 40 | 1.15E-26 | 1.97E-23 |
|  | GOTERM_BP_DIRECT | GO:0050776~regulation of immune response | 43 | 1.50E-26 | 2.58E-23 |
|  | GOTERM_BP_DIRECT | GO:0031295~T cell costimulation | 29 | 1.32E-23 | 2.28E-20 |
|  | GOTERM_BP_DIRECT | GO:0045087~innate immune response | 55 | 5.35E-20 | 9.21E-17 |
|  | GOTERM_BP_DIRECT | GO:0042102~positive regulation of T cell proliferation | 23 | 4.74E-19 | 8.15E-16 |
|  | GOTERM_BP_DIRECT | GO:0006968~cellular defense response | 19 | 9.10E-14 | 1.57E-10 |
|  | GOTERM_BP_DIRECT | GO:0007165~signal transduction | 82 | 1.08E-13 | 1.86E-10 |
|  | GOTERM_BP_DIRECT | GO:0050900~leukocyte migration | 24 | 7.72E-13 | 1.33E-09 |
|  |  |  |  |  |  |
| ESCA | GOTERM_BP_DIRECT | GO:0006955~immune response | 96 | 7.26E-47 | 1.28E-43 |
|  | GOTERM_BP_DIRECT | GO:0002250~adaptive immune response | 51 | 5.60E-34 | 9.87E-31 |
|  | GOTERM_BP_DIRECT | GO:0006954~inflammatory response | 73 | 2.10E-30 | 3.69E-27 |
|  | GOTERM_BP_DIRECT | GO:0050776~regulation of immune response | 48 | 1.17E-26 | 2.07E-23 |
|  | GOTERM_BP_DIRECT | GO:0031295~T cell costimulation | 32 | 5.44E-24 | 9.57E-21 |
|  | GOTERM_BP_DIRECT | GO:0007165~signal transduction | 114 | 7.08E-21 | 1.25E-17 |
|  | GOTERM_BP_DIRECT | GO:0050900~leukocyte migration | 32 | 1.75E-17 | 3.09E-14 |
|  | GOTERM_BP_DIRECT | GO:0042102~positive regulation of T cell proliferation | 23 | 1.60E-16 | 2.00E-13 |
|  | GOTERM_BP_DIRECT | GO:0045087~innate immune response | 57 | 7.87E-16 | 1.37E-12 |
|  | GOTERM_BP_DIRECT | GO:0006935~chemotaxis | 30 | 1.53E-15 | 2.74E-12 |
|  |  |  |  |  |  |
| GBM | GOTERM_BP_DIRECT | GO:0006955~immune response | 107 | 6.98E-35 | 1.29E-31 |
|  | GOTERM_BP_DIRECT | GO:0006954~inflammatory response | 89 | 2.38E-26 | 4.40E-23 |
|  | GOTERM_BP_DIRECT | GO:0045087~innate immune response | 79 | 9.36E-17 | 2.11E-13 |
|  | GOTERM_BP_DIRECT | GO:0050776~regulation of immune response | 47 | 4.06E-16 | 8.22E-13 |
|  | GOTERM_BP_DIRECT | GO:0006935~chemotaxis | 35 | 2.49E-13 | 4.62E-10 |
|  | GOTERM_BP_DIRECT | GO:0002250~adaptive immune response | 37 | 4.21E-12 | 7.80E-09 |
|  | GOTERM_BP_DIRECT | GO:0042102~positive regulation of T cell proliferation | 23 | 1.00E-11 | 1.85E-08 |
|  | GOTERM_BP_DIRECT | GO:0030593~neutrophil chemotaxis | 23 | 8.82E-11 | 1.63E-07 |
|  | GOTERM_BP_DIRECT | GO:0031295~T cell costimulation | 24 | 5.33E-10 | 9.87E-07 |
|  | GOTERM_BP_DIRECT | GO:0007165~signal transduction | 129 | 2.77E-09 | 5.13E-06 |
|  |  |  |  |  |  |
| HNSC | GOTERM_BP_DIRECT | GO:0006955~immune response | 94 | 7.57E-49 | 1.32E-45 |
|  | GOTERM_BP_DIRECT | GO:0002250~adaptive immune response | 47 | 2.01E-31 | 3.50E-28 |
|  | GOTERM_BP_DIRECT | GO:0006954~inflammatory response | 69 | 8.18E-30 | 1.42E-26 |
|  | GOTERM_BP_DIRECT | GO:0050776~regulation of immune response | 45 | 1.64E-25 | 2.85E-22 |
|  | GOTERM_BP_DIRECT | GO:0031295~T cell costimulation | 32 | 2.73E-25 | 4.75E-22 |
|  | GOTERM_BP_DIRECT | GO:0045087~innate immune response | 59 | 3.15E-19 | 5.48E-16 |
|  | GOTERM_BP_DIRECT | GO:0042102~positive regulation of T cell proliferation | 24 | 1.13E-18 | 1.97E-15 |
|  | GOTERM_BP_DIRECT | GO:0006935~chemotaxis | 31 | 1.02E-17 | 1.78E-14 |
|  | GOTERM_BP_DIRECT | GO:0007166~cell surface receptor signaling pathway | 44 | 5.90E-17 | 1.89E-13 |
|  | GOTERM_BP_DIRECT | GO:0007165~signal transduction | 96 | 2.13E-15 | 3.66E-12 |
|  |  |  |  |  |  |
| KICH | GOTERM_BP_DIRECT | GO:0006955~immune response | 110 | 1.48E-43 | 2.70E-40 |
|  | GOTERM_BP_DIRECT | GO:0006954~inflammatory response | 100 | 6.27E-40 | 1.14E-36 |
|  | GOTERM_BP_DIRECT | GO:0007165~signal transduction | 160 | 3.27E-27 | 5.97E-24 |
|  | GOTERM_BP_DIRECT | GO:0045087~innate immune response | 87 | 8.99E-26 | 1.64E-22 |
|  | GOTERM_BP_DIRECT | GO:0050776~regulation of immune response | 49 | 1.70E-20 | 3.09E-17 |
|  | GOTERM_BP_DIRECT | GO:0002250~adaptive immune response | 43 | 5.31E-19 | 9.68E-16 |
|  | GOTERM_BP_DIRECT | GO:0006935~chemotaxis | 38 | 5.86E-18 | 1.07E-14 |
|  | GOTERM_BP_DIRECT | GO:0050900~leukocyte migration | 37 | 4.39E-17 | 8.00E-14 |
|  | GOTERM_BP_DIRECT | GO:0031295~T cell costimulation | 30 | 4.69E-17 | 8.54E-14 |
|  | GOTERM_BP_DIRECT | GO:0071222~cellular response to lipopolysaccharide | 31 | 4.54E-13 | 8.27E-10 |
|  |  |  |  |  |  |
| KIRC | GOTERM_BP_DIRECT | GO:0006955~immune response | 29 | 5.47E-10 | 9.04E-07 |
|  | GOTERM_BP_DIRECT | GO:0042110~T cell activation | 9 | 9.94E-07 | 0.001642 |
|  | GOTERM_BP_DIRECT | GO:0050852~T cell receptor signaling pathway | 14 | 1.25E-06 | 0.002058 |
|  | GOTERM_BP_DIRECT | GO:0050776~regulation of immune response | 15 | 1.84E-06 | 0.003033 |
|  | GOTERM_BP_DIRECT | GO:0043547~positive regulation of GTPase activity | 26 | 1.00E-05 | 0.016555 |
|  | GOTERM_BP_DIRECT | GO:0031295~T cell costimulation | 9 | 4.74E-05 | 0.078185 |
|  | GOTERM_BP_DIRECT | GO:0038083~peptidyl-tyrosine autophosphorylation | 7 | 4.95E-05 | 0.081771 |
|  | GOTERM_BP_DIRECT | GO:0030036~actin cytoskeleton organization | 11 | 6.69E-05 | 0.110432 |
|  | GOTERM_BP_DIRECT | GO:0006954~inflammatory response | 19 | 7.37E-05 | 0.121588 |
|  | GOTERM_BP_DIRECT | GO:0009615~response to virus | 10 | 9.62E-05 | 0.158689 |
|  |  |  |  |  |  |
| KIRP | GOTERM_BP_DIRECT | GO:0006955~immune response | 104 | 1.82E-51 | 3.23E-48 |
|  | GOTERM_BP_DIRECT | GO:0006954~inflammatory response | 88 | 4.91E-41 | 8.72E-38 |
|  | GOTERM_BP_DIRECT | GO:0045087~innate immune response | 80 | 3.82E-30 | 6.79E-27 |
|  | GOTERM_BP_DIRECT | GO:0050776~regulation of immune response | 49 | 2.73E-26 | 4.86E-23 |
|  | GOTERM_BP_DIRECT | GO:0002250~adaptive immune response | 44 | 4.21E-25 | 7.49E-22 |
|  | GOTERM_BP_DIRECT | GO:0031295~T cell costimulation | 30 | 1.04E-20 | 1.85E-17 |
|  | GOTERM_BP_DIRECT | GO:0006935~chemotaxis | 34 | 1.65E-18 | 2.94E-15 |
|  | GOTERM_BP_DIRECT | GO:0007165~signal transduction | 108 | 1.20E-15 | 2.18E-12 |
|  | GOTERM_BP_DIRECT | GO:0050852~T cell receptor signaling pathway | 33 | 6.86E-15 | 1.22E-11 |
|  | GOTERM_BP_DIRECT | GO:0030593~neutrophil chemotaxis | 23 | 7.54E-15 | 1.34E-11 |
|  |  |  |  |  |  |
| DLBC | GOTERM_BP_DIRECT | GO:0006120~mitochondrial electron transport, NADH to ubiquinone | 22 | 2.94E-11 | 5.39E-08 |
|  | GOTERM_BP_DIRECT | GO:0070125~mitochondrial translational elongation | 29 | 3.09E-11 | 5.66E-08 |
|  | GOTERM_BP_DIRECT | GO:0070126~mitochondrial translational termination | 29 | 4.23E-11 | 7.77E-08 |
|  | GOTERM_BP_DIRECT | GO:0032981~mitochondrial respiratory chain complex I assembly | 23 | 1.13E-09 | 2.08E-06 |
|  | GOTERM_BP_DIRECT | GO:0006413~translational initiation | 35 | 1.38E-09 | 2.53E-06 |
|  | GOTERM_BP_DIRECT | GO:0043161~proteasome-mediated ubiquitin-dependent protein catabolic process | 42 | 2.06E-08 | 3.78E-05 |
|  | GOTERM_BP_DIRECT | GO:0006412~translation | 48 | 3.08E-08 | 5.65E-05 |
|  | GOTERM_BP_DIRECT | GO:0006364~rRNA processing | 42 | 9.64E-08 | 1.77E-04 |
|  | GOTERM_BP_DIRECT | GO:0038061~NIK/NF-kappaB signaling | 21 | 9.84E-08 | 1.81E-04 |
|  | GOTERM_BP_DIRECT | GO:0002223~stimulatory C-type lectin receptor signaling pathway | 27 | 1.23E-07 | 2.25E-04 |
|  |  |  |  |  |  |
| LIHC | GOTERM_BP_DIRECT | GO:0006955~immune response | 85 | 8.02E-42 | 1.40E-38 |
|  | GOTERM_BP_DIRECT | GO:0006954~inflammatory response | 74 | 2.95E-35 | 5.14E-32 |
|  | GOTERM_BP_DIRECT | GO:0002250~adaptive immune response | 43 | 1.06E-27 | 1.85E-24 |
|  | GOTERM_BP_DIRECT | GO:0031295~T cell costimulation | 32 | 8.08E-26 | 1.41E-22 |
|  | GOTERM_BP_DIRECT | GO:0050776~regulation of immune response | 40 | 2.46E-21 | 4.28E-18 |
|  | GOTERM_BP_DIRECT | GO:0007165~signal transduction | 105 | 3.66E-21 | 6.37E-18 |
|  | GOTERM_BP_DIRECT | GO:0045087~innate immune response | 56 | 5.07E-18 | 8.83E-15 |
|  | GOTERM_BP_DIRECT | GO:0042102~positive regulation of T cell proliferation | 23 | 8.48E-18 | 1.48E-14 |
|  | GOTERM_BP_DIRECT | GO:0006935~chemotaxis | 30 | 3.31E-17 | 5.77E-14 |
|  | GOTERM_BP_DIRECT | GO:0050852~T cell receptor signaling pathway | 29 | 6.51E-14 | 1.13E-10 |
|  |  |  |  |  |  |
| LGG | GOTERM_BP_DIRECT | GO:0006954~inflammatory response | 93 | 6.80E-28 | 1.26E-24 |
|  | GOTERM_BP_DIRECT | GO:0006955~immune response | 98 | 1.79E-27 | 3.31E-24 |
|  | GOTERM_BP_DIRECT | GO:0060333~interferon-gamma-mediated signaling pathway | 36 | 1.64E-22 | 3.04E-19 |
|  | GOTERM_BP_DIRECT | GO:0045087~innate immune response | 88 | 9.99E-21 | 1.85E-17 |
|  | GOTERM_BP_DIRECT | GO:0007165~signal transduction | 150 | 1.24E-14 | 2.29E-11 |
|  | GOTERM_BP_DIRECT | GO:0002224~toll-like receptor signaling pathway | 17 | 1.22E-12 | 2.27E-09 |
|  | GOTERM_BP_DIRECT | GO:0002250~adaptive immune response | 38 | 2.51E-12 | 4.66E-09 |
|  | GOTERM_BP_DIRECT | GO:0019882~antigen processing and presentation | 23 | 2.58E-12 | 4.78E-09 |
|  | GOTERM_BP_DIRECT | GO:0050776~regulation of immune response | 42 | 2.98E-12 | 5.53E-09 |
|  | GOTERM_BP_DIRECT | GO:0031295~T cell costimulation | 27 | 3.86E-12 | 7.16E-09 |
|  |  |  |  |  |  |
| LUAD | GOTERM_BP_DIRECT | GO:0006955~immune response | 104 | 7.72E-59 | 1.34E-55 |
|  | GOTERM_BP_DIRECT | GO:0006954~inflammatory response | 81 | 2.13E-40 | 3.71E-37 |
|  | GOTERM_BP_DIRECT | GO:0050776~regulation of immune response | 56 | 2.99E-37 | 5.19E-34 |
|  | GOTERM_BP_DIRECT | GO:0002250~adaptive immune response | 47 | 2.01E-31 | 3.50E-28 |
|  | GOTERM_BP_DIRECT | GO:0045087~innate immune response | 73 | 1.44E-29 | 2.50E-26 |
|  | GOTERM_BP_DIRECT | GO:0031295~T cell costimulation | 32 | 2.73E-25 | 4.74E-22 |
|  | GOTERM_BP_DIRECT | GO:0007165~signal transduction | 102 | 2.29E-18 | 3.98E-15 |
|  | GOTERM_BP_DIRECT | GO:0007166~cell surface receptor signaling pathway | 45 | 1.04E-17 | 1.81E-14 |
|  | GOTERM_BP_DIRECT | GO:0042102~positive regulation of T cell proliferation | 23 | 1.98E-17 | 3.44E-14 |
|  | GOTERM_BP_DIRECT | GO:0060333~interferon-gamma-mediated signaling pathway | 23 | 1.31E-15 | 2.31E-12 |
|  |  |  |  |  |  |
| LUSC | GOTERM_BP_DIRECT | GO:0006955~immune response | 135 | 2.34E-66 | 4.26E-63 |
|  | GOTERM_BP_DIRECT | GO:0006954~inflammatory response | 106 | 2.00E-45 | 3.64E-42 |
|  | GOTERM_BP_DIRECT | GO:0002250~adaptive immune response | 60 | 1.23E-35 | 2.24E-32 |
|  | GOTERM_BP_DIRECT | GO:0050776~regulation of immune response | 60 | 1.76E-30 | 3.21E-27 |
|  | GOTERM_BP_DIRECT | GO:0031295~T cell costimulation | 36 | 1.00E-23 | 1.82E-20 |
|  | GOTERM_BP_DIRECT | GO:0045087~innate immune response | 83 | 1.58E-23 | 2.87E-20 |
|  | GOTERM_BP_DIRECT | GO:0007165~signal transduction | 145 | 6.23E-21 | 1.13E-17 |
|  | GOTERM_BP_DIRECT | GO:0006935~chemotaxis | 41 | 7.24E-21 | 1.32E-17 |
|  | GOTERM_BP_DIRECT | GO:0042102~positive regulation of T cell proliferation | 27 | 1.66E-17 | 3.02E-14 |
|  | GOTERM_BP_DIRECT | GO:0060333~interferon-gamma-mediated signaling pathway | 29 | 1.94E-17 | 3.54E-14 |
|  |  |  |  |  |  |
| SKCM | GOTERM_BP_DIRECT | GO:0006955~immune response | 126 | 2.41E-66 | 4.32E-63 |
|  | GOTERM_BP_DIRECT | GO:0006954~inflammatory response | 108 | 3.40E-54 | 6.09E-51 |
|  | GOTERM_BP_DIRECT | GO:0002250~adaptive immune response | 60 | 1.28E-39 | 2.29E-36 |
|  | GOTERM_BP_DIRECT | GO:0050776~regulation of immune response | 63 | 1.69E-37 | 3.02E-34 |
|  | GOTERM_BP_DIRECT | GO:0045087~innate immune response | 90 | 1.77E-33 | 3.16E-30 |
|  | GOTERM_BP_DIRECT | GO:0031295~T cell costimulation | 36 | 4.33E-26 | 7.76E-23 |
|  | GOTERM_BP_DIRECT | GO:0006935~chemotaxis | 41 | 1.89E-23 | 3.38E-20 |
|  | GOTERM_BP_DIRECT | GO:0042102~positive regulation of T cell proliferation | 30 | 5.44E-23 | 9.74E-20 |
|  | GOTERM_BP_DIRECT | GO:0007165~signal transduction | 131 | 2.98E-21 | 5.34E-18 |
|  | GOTERM_BP_DIRECT | GO:0060333~interferon-gamma-mediated signaling pathway | 28 | 3.65E-18 | 6.54E-15 |
|  |  |  |  |  |  |
| MESO | GOTERM_BP_DIRECT | GO:0006955~immune response | 75 | 9.16E-35 | 1.60E-31 |
|  | GOTERM_BP_DIRECT | GO:0006954~inflammatory response | 64 | 3.21E-28 | 5.61E-25 |
|  | GOTERM_BP_DIRECT | GO:0031295~T cell costimulation | 27 | 3.06E-20 | 5.35E-17 |
|  | GOTERM_BP_DIRECT | GO:0045087~innate immune response | 57 | 6.40E-20 | 1.12E-16 |
|  | GOTERM_BP_DIRECT | GO:0002250~adaptive immune response | 34 | 2.75E-19 | 4.81E-16 |
|  | GOTERM_BP_DIRECT | GO:0042102~positive regulation of T cell proliferation | 23 | 2.34E-18 | 4.09E-15 |
|  | GOTERM_BP_DIRECT | GO:0050776~regulation of immune response | 33 | 8.39E-16 | 1.55E-12 |
|  | GOTERM_BP_DIRECT | GO:0006935~chemotaxis | 26 | 5.57E-14 | 9.73E-11 |
|  | GOTERM_BP_DIRECT | GO:0050900~leukocyte migration | 24 | 3.61E-12 | 6.32E-09 |
|  | GOTERM_BP_DIRECT | GO:0050852~T cell receptor signaling pathway | 26 | 5.34E-12 | 9.34E-09 |
|  |  |  |  |  |  |
| UVM | GOTERM_BP_DIRECT | GO:0006955~immune response | 125 | 3.52E-57 | 6.45E-54 |
|  | GOTERM_BP_DIRECT | GO:0006954~inflammatory response | 111 | 6.04E-50 | 1.11E-46 |
|  | GOTERM_BP_DIRECT | GO:0002250~adaptive immune response | 51 | 1.66E-26 | 3.05E-23 |
|  | GOTERM_BP_DIRECT | GO:0050776~regulation of immune response | 52 | 2.73E-23 | 5.01E-20 |
|  | GOTERM_BP_DIRECT | GO:0031295~T cell costimulation | 33 | 2.14E-20 | 3.93E-17 |
|  | GOTERM_BP_DIRECT | GO:0007165~signal transduction | 142 | 8.54E-20 | 1.57E-16 |
|  | GOTERM_BP_DIRECT | GO:0060333~interferon-gamma-mediated signaling pathway | 31 | 1.20E-19 | 2.20E-16 |
|  | GOTERM_BP_DIRECT | GO:0006935~chemotaxis | 39 | 4.54E-19 | 8.33E-16 |
|  | GOTERM_BP_DIRECT | GO:0045087~innate immune response | 73 | 1.31E-17 | 2.40E-14 |
|  | GOTERM_BP_DIRECT | GO:0050900~leukocyte migration | 37 | 2.73E-17 | 5.02E-14 |
|  |  |  |  |  |  |
| OV | GOTERM_BP_DIRECT | GO:0006955~immune response | 109 | 1.31E-68 | 2.30E-65 |
|  | GOTERM_BP_DIRECT | GO:0006954~inflammatory response | 87 | 1.45E-49 | 2.53E-46 |
|  | GOTERM_BP_DIRECT | GO:0050776~regulation of immune response | 51 | 1.04E-33 | 1.82E-30 |
|  | GOTERM_BP_DIRECT | GO:0002250~adaptive immune response | 47 | 2.88E-33 | 5.04E-30 |
|  | GOTERM_BP_DIRECT | GO:0045087~innate immune response | 65 | 9.64E-26 | 1.69E-22 |
|  | GOTERM_BP_DIRECT | GO:0007165~signal transduction | 109 | 2.76E-25 | 4.83E-22 |
|  | GOTERM_BP_DIRECT | GO:0031295~T cell costimulation | 30 | 6.33E-24 | 1.11E-20 |
|  | GOTERM_BP_DIRECT | GO:0006935~chemotaxis | 35 | 3.94E-23 | 6.89E-20 |
|  | GOTERM_BP_DIRECT | GO:0050900~leukocyte migration | 31 | 7.30E-19 | 1.28E-15 |
|  | GOTERM_BP_DIRECT | GO:0032729~positive regulation of interferon-gamma production | 21 | 1.64E-18 | 2.87E-15 |
|  |  |  |  |  |  |
| PAAD | GOTERM_BP_DIRECT | GO:0006955~immune response | 116 | 1.42E-49 | 2.57E-46 |
|  | GOTERM_BP_DIRECT | GO:0006954~inflammatory response | 103 | 2.65E-43 | 4.81E-40 |
|  | GOTERM_BP_DIRECT | GO:0002250~adaptive immune response | 53 | 1.28E-28 | 2.33E-25 |
|  | GOTERM_BP_DIRECT | GO:0050776~regulation of immune response | 55 | 4.38E-26 | 7.95E-23 |
|  | GOTERM_BP_DIRECT | GO:0045087~innate immune response | 83 | 7.79E-24 | 1.41E-20 |
|  | GOTERM_BP_DIRECT | GO:0031295~T cell costimulation | 35 | 9.93E-23 | 1.80E-19 |
|  | GOTERM_BP_DIRECT | GO:0007166~cell surface receptor signaling pathway | 61 | 8.14E-21 | 1.48E-17 |
|  | GOTERM_BP_DIRECT | GO:0007165~signal transduction | 141 | 1.12E-19 | 2.04E-16 |
|  | GOTERM_BP_DIRECT | GO:0007155~cell adhesion | 75 | 2.58E-17 | 4.68E-14 |
|  | GOTERM_BP_DIRECT | GO:0050900~leukocyte migration | 35 | 1.34E-15 | 2.42E-12 |
|  |  |  |  |  |  |
| PCPG | GOTERM_BP_DIRECT | GO:0001525~angiogenesis | 66 | 2.22E-25 | 4.14E-22 |
|  | GOTERM_BP_DIRECT | GO:0001570~vasculogenesis | 21 | 1.76E-10 | 3.28E-07 |
|  | GOTERM_BP_DIRECT | GO:0030198~extracellular matrix organization | 40 | 4.71E-10 | 8.77E-07 |
|  | GOTERM_BP_DIRECT | GO:0006955~immune response | 63 | 2.21E-09 | 4.12E-06 |
|  | GOTERM_BP_DIRECT | GO:0030335~positive regulation of cell migration | 36 | 1.24E-08 | 2.32E-05 |
|  | GOTERM_BP_DIRECT | GO:0043547~positive regulation of GTPase activity | 74 | 2.68E-08 | 4.98E-05 |
|  | GOTERM_BP_DIRECT | GO:0051056~regulation of small GTPase mediated signal transduction | 29 | 4.40E-08 | 8.18E-05 |
|  | GOTERM_BP_DIRECT | GO:0006954~inflammatory response | 55 | 7.54E-08 | 1.40E-04 |
|  | GOTERM_BP_DIRECT | GO:0030168~platelet activation | 26 | 1.02E-07 | 1.90E-04 |
|  | GOTERM_BP_DIRECT | GO:0007165~signal transduction | 122 | 2.88E-07 | 5.37E-04 |
|  |  |  |  |  |  |
| PRAD | GOTERM_BP_DIRECT | GO:0006955~immune response | 109 | 7.30E-50 | 1.32E-46 |
|  | GOTERM_BP_DIRECT | GO:0006954~inflammatory response | 85 | 1.14E-33 | 2.05E-30 |
|  | GOTERM_BP_DIRECT | GO:0050776~regulation of immune response | 50 | 1.82E-24 | 3.29E-21 |
|  | GOTERM_BP_DIRECT | GO:0031295~T cell costimulation | 32 | 3.11E-21 | 5.60E-18 |
|  | GOTERM_BP_DIRECT | GO:0007165~signal transduction | 125 | 2.53E-18 | 4.55E-15 |
|  | GOTERM_BP_DIRECT | GO:0045087~innate immune response | 66 | 5.28E-17 | 9.52E-14 |
|  | GOTERM_BP_DIRECT | GO:0042102~positive regulation of T cell proliferation | 25 | 8.05E-17 | 2.00E-13 |
|  | GOTERM_BP_DIRECT | GO:0002250~adaptive immune response | 37 | 1.95E-16 | 4.00E-13 |
|  | GOTERM_BP_DIRECT | GO:0006935~chemotaxis | 31 | 5.34E-14 | 9.62E-11 |
|  | GOTERM_BP_DIRECT | GO:0070098~chemokine-mediated signaling pathway | 22 | 7.11E-12 | 1.28E-08 |
|  |  |  |  |  |  |
| SARC | GOTERM_BP_DIRECT | GO:0006955~immune response | 127 | 8.75E-49 | 1.62E-45 |
|  | GOTERM_BP_DIRECT | GO:0006954~inflammatory response | 119 | 1.16E-47 | 2.13E-44 |
|  | GOTERM_BP_DIRECT | GO:0002250~adaptive immune response | 57 | 6.07E-28 | 1.12E-24 |
|  | GOTERM_BP_DIRECT | GO:0050776~regulation of immune response | 62 | 1.41E-27 | 2.59E-24 |
|  | GOTERM_BP_DIRECT | GO:0045087~innate immune response | 92 | 3.73E-23 | 6.89E-20 |
|  | GOTERM_BP_DIRECT | GO:0031295~T cell costimulation | 38 | 5.02E-23 | 9.27E-20 |
|  | GOTERM_BP_DIRECT | GO:0006935~chemotaxis | 43 | 1.75E-19 | 3.23E-16 |
|  | GOTERM_BP_DIRECT | GO:0007165~signal transduction | 153 | 7.96E-16 | 1.43E-12 |
|  | GOTERM_BP_DIRECT | GO:0042102~positive regulation of T cell proliferation | 26 | 2.46E-14 | 4.53E-11 |
|  | GOTERM_BP_DIRECT | GO:0060333~interferon-gamma-mediated signaling pathway | 28 | 3.38E-14 | 6.23E-11 |
|  |  |  |  |  |  |
| STAD | GOTERM_BP_DIRECT | GO:0006955~immune response | 115 | 2.12E-46 | 3.90E-43 |
|  | GOTERM_BP_DIRECT | GO:0006954~inflammatory response | 100 | 8.11E-39 | 1.49E-35 |
|  | GOTERM_BP_DIRECT | GO:0050776~regulation of immune response | 52 | 1.98E-22 | 3.64E-19 |
|  | GOTERM_BP_DIRECT | GO:0007155~cell adhesion | 86 | 2.77E-22 | 5.08E-19 |
|  | GOTERM_BP_DIRECT | GO:0007165~signal transduction | 151 | 8.86E-22 | 1.63E-18 |
|  | GOTERM_BP_DIRECT | GO:0002250~adaptive immune response | 46 | 4.32E-21 | 7.93E-18 |
|  | GOTERM_BP_DIRECT | GO:0045087~innate immune response | 80 | 1.45E-20 | 2.67E-17 |
|  | GOTERM_BP_DIRECT | GO:0050900~leukocyte migration | 35 | 6.03E-15 | 1.10E-11 |
|  | GOTERM_BP_DIRECT | GO:0006935~chemotaxis | 33 | 2.42E-13 | 4.44E-10 |
|  | GOTERM_BP_DIRECT | GO:0050853~B cell receptor signaling pathway | 22 | 6.72E-13 | 1.24E-09 |
|  |  |  |  |  |  |
| TGCT | GOTERM_BP_DIRECT | GO:0006955~immune response | 140 | 2.80E-67 | 5.13E-64 |
|  | GOTERM_BP_DIRECT | GO:0006954~inflammatory response | 108 | 2.78E-44 | 5.09E-41 |
|  | GOTERM_BP_DIRECT | GO:0002250~adaptive immune response | 61 | 4.85E-35 | 8.89E-32 |
|  | GOTERM_BP_DIRECT | GO:0045087~innate immune response | 96 | 5.21E-30 | 9.56E-27 |
|  | GOTERM_BP_DIRECT | GO:0050776~regulation of immune response | 61 | 8.21E-30 | 1.51E-26 |
|  | GOTERM_BP_DIRECT | GO:0060333~interferon-gamma-mediated signaling pathway | 39 | 3.40E-28 | 6.24E-25 |
|  | GOTERM_BP_DIRECT | GO:0031295~T cell costimulation | 38 | 4.85E-25 | 8.89E-22 |
|  | GOTERM_BP_DIRECT | GO:0007165~signal transduction | 149 | 8.57E-20 | 1.57E-16 |
|  | GOTERM_BP_DIRECT | GO:0071222~cellular response to lipopolysaccharide | 38 | 2.13E-18 | 3.91E-15 |
|  | GOTERM_BP_DIRECT | GO:0042102~positive regulation of T cell proliferation | 26 | 1.16E-15 | 2.03E-12 |
|  |  |  |  |  |  |
| THYM | GOTERM_BP_DIRECT | GO:0006412~translation | 85 | 3.83E-12 | 7.39E-09 |
|  | GOTERM_BP_DIRECT | GO:0019083~viral transcription | 49 | 7.06E-12 | 1.36E-08 |
|  | GOTERM_BP_DIRECT | GO:0000398~mRNA splicing, via spliceosome | 77 | 7.43E-12 | 1.43E-08 |
|  | GOTERM_BP_DIRECT | GO:0006364~rRNA processing | 75 | 8.06E-12 | 1.56E-08 |
|  | GOTERM_BP_DIRECT | GO:0006614~SRP-dependent cotranslational protein targeting to membrane | 42 | 1.22E-10 | 2.35E-07 |
|  | GOTERM_BP_DIRECT | GO:0050852~T cell receptor signaling pathway | 56 | 1.76E-10 | 3.39E-07 |
|  | GOTERM_BP_DIRECT | GO:0006413~translational initiation | 52 | 7.46E-10 | 1.44E-06 |
|  | GOTERM_BP_DIRECT | GO:0000184~nuclear-transcribed mRNA catabolic process, nonsense-mediated decay | 46 | 4.14E-09 | 7.98E-06 |
|  | GOTERM_BP_DIRECT | GO:0006120~mitochondrial electron transport, NADH to ubiquinone | 25 | 5.56E-08 | 1.07E-04 |
|  | GOTERM_BP_DIRECT | GO:0098609~cell-cell adhesion | 77 | 1.68E-07 | 3.24E-04 |
|  |  |  |  |  |  |
| THCA | GOTERM_BP_DIRECT | GO:0006954~inflammatory response | 50 | 1.42E-14 | 2.49E-11 |
|  | GOTERM_BP_DIRECT | GO:0045087~innate immune response | 53 | 3.35E-14 | 5.89E-11 |
|  | GOTERM_BP_DIRECT | GO:0006955~immune response | 52 | 5.65E-14 | 9.92E-11 |
|  | GOTERM_BP_DIRECT | GO:0050776~regulation of immune response | 32 | 4.36E-13 | 7.65E-10 |
|  | GOTERM_BP_DIRECT | GO:0002250~adaptive immune response | 25 | 8.96E-10 | 1.57E-06 |
|  | GOTERM_BP_DIRECT | GO:0050852~T cell receptor signaling pathway | 24 | 4.70E-09 | 8.26E-06 |
|  | GOTERM_BP_DIRECT | GO:0033209~tumor necrosis factor-mediated signaling pathway | 21 | 1.04E-08 | 1.83E-05 |
|  | GOTERM_BP_DIRECT | GO:0002223~stimulatory C-type lectin receptor signaling pathway | 19 | 4.73E-08 | 8.30E-05 |
|  | GOTERM_BP_DIRECT | GO:0007169~transmembrane receptor protein tyrosine kinase signaling pathway | 18 | 6.80E-08 | 1.19E-04 |
|  | GOTERM_BP_DIRECT | GO:0006935~chemotaxis | 20 | 9.93E-08 | 1.74E-04 |
|  |  |  |  |  |  |
| UCS | GOTERM_BP_DIRECT | GO:0006955~immune response | 59 | 2.11E-29 | 3.59E-26 |
|  | GOTERM_BP_DIRECT | GO:0050776~regulation of immune response | 35 | 3.42E-22 | 5.83E-19 |
|  | GOTERM_BP_DIRECT | GO:0006954~inflammatory response | 46 | 2.77E-20 | 4.71E-17 |
|  | GOTERM_BP_DIRECT | GO:0045087~innate immune response | 45 | 2.26E-17 | 3.84E-14 |
|  | GOTERM_BP_DIRECT | GO:0002250~adaptive immune response | 26 | 2.87E-15 | 4.92E-12 |
|  | GOTERM_BP_DIRECT | GO:0006935~chemotaxis | 23 | 3.33E-14 | 5.67E-11 |
|  | GOTERM_BP_DIRECT | GO:0007165~signal transduction | 65 | 1.47E-11 | 2.50E-08 |
|  | GOTERM_BP_DIRECT | GO:0042102~positive regulation of T cell proliferation | 15 | 4.27E-11 | 7.27E-08 |
|  | GOTERM_BP_DIRECT | GO:0050900~leukocyte migration | 18 | 1.88E-09 | 3.20E-06 |
|  | GOTERM_BP_DIRECT | GO:0007166~cell surface receptor signaling pathway | 26 | 2.91E-09 | 4.95E-06 |

**Table S3.** The top 10 most significant items of cellular components of GMFG in 32 individual cancer types.

| Cancer type | Category | Term | Gene count | P-Value | FDR |
| --- | --- | --- | --- | --- | --- |
| LAML | GOTERM_CC_DIRECT | GO:0070062~extracellular exosome | 56 | 4.93E-06 | 0.006592 |
|  | GOTERM_CC_DIRECT | GO:0016020~membrane | 46 | 1.57E-05 | 0.021004 |
|  | GOTERM_CC_DIRECT | GO:0005768~endosome | 12 | 3.47E-05 | 0.046294 |
|  | GOTERM_CC_DIRECT | GO:0031982~vesicle | 9 | 7.64E-05 | 0.102086 |
|  | GOTERM_CC_DIRECT | GO:0005829~cytosol | 58 | 1.42E-04 | 0.189658 |
|  | GOTERM_CC_DIRECT | GO:0030670~phagocytic vesicle membrane | 6 | 4.38E-04 | 0.583055 |
|  | GOTERM_CC_DIRECT | GO:0005903~brush border | 6 | 4.73E-04 | 0.630402 |
|  | GOTERM_CC_DIRECT | GO:0015629~actin cytoskeleton | 10 | 6.26E-04 | 0.833854 |
|  | GOTERM_CC_DIRECT | GO:0005813~centrosome | 14 | 7.72E-04 | 1.026465 |
|  | GOTERM_CC_DIRECT | GO:0005737~cytoplasm | 77 | 0.001617 | 2.13998 |
|  |  |  |  |  |  |
| ACC | GOTERM_CC_DIRECT | GO:0005886~plasma membrane | 308 | 3.59E-34 | 4.92E-31 |
|  | GOTERM_CC_DIRECT | GO:0009897~external side of plasma membrane | 53 | 8.15E-27 | 1.12E-23 |
|  | GOTERM_CC_DIRECT | GO:0005887~integral component of plasma membrane | 142 | 2.14E-25 | 2.94E-22 |
|  | GOTERM_CC_DIRECT | GO:0042101~T cell receptor complex | 14 | 3.93E-15 | 5.32E-12 |
|  | GOTERM_CC_DIRECT | GO:0016021~integral component of membrane | 296 | 1.39E-13 | 1.91E-10 |
|  | GOTERM_CC_DIRECT | GO:0009986~cell surface | 62 | 1.80E-13 | 2.47E-10 |
|  | GOTERM_CC_DIRECT | GO:0042613~MHC class II protein complex | 12 | 1.68E-10 | 2.30E-07 |
|  | GOTERM_CC_DIRECT | GO:0001772~immunological synapse | 14 | 2.33E-10 | 3.19E-07 |
|  | GOTERM_CC_DIRECT | GO:0030666~endocytic vesicle membrane | 15 | 2.41E-07 | 3.30E-04 |
|  | GOTERM_CC_DIRECT | GO:0071556~integral component of luminal side of endoplasmic reticulum membrane | 10 | 1.14E-06 | 0.001556 |
|  |  |  |  |  |  |
| CHOL | GOTERM_CC_DIRECT | GO:0005886~plasma membrane | 356 | 3.25E-39 | 4.52E-36 |
|  | GOTERM_CC_DIRECT | GO:0009897~external side of plasma membrane | 66 | 7.65E-36 | 1.06E-32 |
|  | GOTERM_CC_DIRECT | GO:0005887~integral component of plasma membrane | 173 | 9.34E-34 | 1.30E-30 |
|  | GOTERM_CC_DIRECT | GO:0009986~cell surface | 66 | 1.27E-12 | 1.77E-09 |
|  | GOTERM_CC_DIRECT | GO:0016021~integral component of membrane | 322 | 1.10E-10 | 1.53E-07 |
|  | GOTERM_CC_DIRECT | GO:0001772~immunological synapse | 14 | 1.42E-09 | 1.97E-06 |
|  | GOTERM_CC_DIRECT | GO:0042101~T cell receptor complex | 10 | 2.95E-08 | 4.09E-05 |
|  | GOTERM_CC_DIRECT | GO:0005576~extracellular region | 121 | 6.41E-08 | 8.91E-05 |
|  | GOTERM_CC_DIRECT | GO:0042613~MHC class II protein complex | 10 | 2.55E-07 | 3.54E-04 |
|  | GOTERM_CC_DIRECT | GO:0005615~extracellular space | 100 | 1.97E-06 | 0.002735 |
|  |  |  |  |  |  |
| BLCA | GOTERM_CC_DIRECT | GO:0005886~plasma membrane | 85 | 3.53E-12 | 4.25E-09 |
|  | GOTERM_CC_DIRECT | GO:0009897~external side of plasma membrane | 17 | 5.16E-10 | 6.21E-07 |
|  | GOTERM_CC_DIRECT | GO:0005887~integral component of plasma membrane | 41 | 1.92E-09 | 2.30E-06 |
|  | GOTERM_CC_DIRECT | GO:0009986~cell surface | 18 | 3.68E-05 | 0.04425 |
|  | GOTERM_CC_DIRECT | GO:0016021~integral component of membrane | 78 | 5.40E-05 | 0.064908 |
|  | GOTERM_CC_DIRECT | GO:0001772~immunological synapse | 5 | 3.68E-04 | 0.44163 |
|  | GOTERM_CC_DIRECT | GO:0042101~T cell receptor complex | 4 | 7.39E-04 | 0.8848 |
|  | GOTERM_CC_DIRECT | GO:0016020~membrane | 38 | 0.001184 | 1.414555 |
|  | GOTERM_CC_DIRECT | GO:0005911~cell-cell junction | 8 | 0.001853 | 2.205751 |
|  | GOTERM_CC_DIRECT | GO:0001931~uropod | 3 | 0.005252 | 6.134882 |
|  |  |  |  |  |  |
| BRCA | GOTERM_CC_DIRECT | GO:0009897~external side of plasma membrane | 62 | 8.55E-36 | 1.16E-32 |
|  | GOTERM_CC_DIRECT | GO:0005886~plasma membrane | 304 | 2.79E-33 | 3.79E-30 |
|  | GOTERM_CC_DIRECT | GO:0005887~integral component of plasma membrane | 141 | 2.84E-25 | 3.86E-22 |
|  | GOTERM_CC_DIRECT | GO:0042613~MHC class II protein complex | 13 | 5.66E-12 | 7.68E-09 |
|  | GOTERM_CC_DIRECT | GO:0042101~T cell receptor complex | 12 | 8.02E-12 | 1.09E-08 |
|  | GOTERM_CC_DIRECT | GO:0009986~cell surface | 58 | 1.33E-11 | 1.80E-08 |
|  | GOTERM_CC_DIRECT | GO:0016021~integral component of membrane | 284 | 3.74E-11 | 5.08E-08 |
|  | GOTERM_CC_DIRECT | GO:0071556~integral component of luminal side of endoplasmic reticulum membrane | 13 | 3.52E-10 | 4.78E-07 |
|  | GOTERM_CC_DIRECT | GO:0005576~extracellular region | 113 | 1.45E-09 | 1.97E-06 |
|  | GOTERM_CC_DIRECT | GO:0001772~immunological synapse | 13 | 3.10E-09 | 4.21E-06 |
|  |  |  |  |  |  |
| CESC | GOTERM_CC_DIRECT | GO:0009897~external side of plasma membrane | 64 | 8.85E-43 | 1.17E-39 |
|  | GOTERM_CC_DIRECT | GO:0005886~plasma membrane | 271 | 5.35E-36 | 7.07E-33 |
|  | GOTERM_CC_DIRECT | GO:0005887~integral component of plasma membrane | 138 | 2.89E-32 | 3.82E-29 |
|  | GOTERM_CC_DIRECT | GO:0016021~integral component of membrane | 262 | 1.89E-16 | 2.89E-13 |
|  | GOTERM_CC_DIRECT | GO:0009986~cell surface | 59 | 1.40E-15 | 1.91E-12 |
|  | GOTERM_CC_DIRECT | GO:0042613~MHC class II protein complex | 12 | 2.09E-11 | 2.76E-08 |
|  | GOTERM_CC_DIRECT | GO:0042101~T cell receptor complex | 11 | 4.42E-11 | 5.83E-08 |
|  | GOTERM_CC_DIRECT | GO:0016020~membrane | 125 | 4.81E-10 | 6.36E-07 |
|  | GOTERM_CC_DIRECT | GO:0001772~immunological synapse | 12 | 5.92E-09 | 7.83E-06 |
|  | GOTERM_CC_DIRECT | GO:0071556~integral component of luminal side of endoplasmic reticulum membrane | 10 | 2.22E-07 | 2.93E-04 |
|  |  |  |  |  |  |
| COADREAD | GOTERM_CC_DIRECT | GO:0005886~plasma membrane | 543 | 1.04E-56 | 1.49E-53 |
|  | GOTERM_CC_DIRECT | GO:0005887~integral component of plasma membrane | 254 | 2.46E-45 | 3.51E-42 |
|  | GOTERM_CC_DIRECT | GO:0009897~external side of plasma membrane | 86 | 5.05E-42 | 7.23E-39 |
|  | GOTERM_CC_DIRECT | GO:0005615~extracellular space | 220 | 1.54E-32 | 2.21E-29 |
|  | GOTERM_CC_DIRECT | GO:0009986~cell surface | 125 | 3.01E-32 | 4.30E-29 |
|  | GOTERM_CC_DIRECT | GO:0005576~extracellular region | 237 | 3.19E-28 | 4.56E-25 |
|  | GOTERM_CC_DIRECT | GO:0031012~extracellular matrix | 83 | 1.49E-27 | 2.14E-24 |
|  | GOTERM_CC_DIRECT | GO:0005578~proteinaceous extracellular matrix | 71 | 4.34E-22 | 6.21E-19 |
|  | GOTERM_CC_DIRECT | GO:0016021~integral component of membrane | 513 | 5.66E-19 | 8.10E-16 |
|  | GOTERM_CC_DIRECT | GO:0045121~membrane raft | 45 | 4.40E-11 | 6.30E-08 |
|  |  |  |  |  |  |
| UCEC | GOTERM_CC_DIRECT | GO:0005886~plasma membrane | 226 | 9.48E-27 | 1.26E-23 |
|  | GOTERM_CC_DIRECT | GO:0009897~external side of plasma membrane | 45 | 1.60E-25 | 2.13E-22 |
|  | GOTERM_CC_DIRECT | GO:0005887~integral component of plasma membrane | 114 | 1.47E-24 | 1.96E-21 |
|  | GOTERM_CC_DIRECT | GO:0042101~T cell receptor complex | 12 | 2.39E-13 | 3.19E-10 |
|  | GOTERM_CC_DIRECT | GO:0001772~immunological synapse | 13 | 7.78E-11 | 1.04E-07 |
|  | GOTERM_CC_DIRECT | GO:0016021~integral component of membrane | 211 | 1.15E-09 | 1.53E-06 |
|  | GOTERM_CC_DIRECT | GO:0016020~membrane | 111 | 1.73E-09 | 2.31E-06 |
|  | GOTERM_CC_DIRECT | GO:0009986~cell surface | 43 | 4.54E-09 | 6.05E-06 |
|  | GOTERM_CC_DIRECT | GO:0042105~alpha-beta T cell receptor complex | 5 | 3.18E-06 | 0.004237 |
|  | GOTERM_CC_DIRECT | GO:0043020~NADPH oxidase complex | 5 | 2.68E-04 | 0.357008 |
|  |  |  |  |  |  |
| ESCA | GOTERM_CC_DIRECT | GO:0005886~plasma membrane | 317 | 3.16E-43 | 4.28E-40 |
|  | GOTERM_CC_DIRECT | GO:0005887~integral component of plasma membrane | 169 | 5.08E-43 | 6.88E-40 |
|  | GOTERM_CC_DIRECT | GO:0009897~external side of plasma membrane | 64 | 6.59E-39 | 8.91E-36 |
|  | GOTERM_CC_DIRECT | GO:0016021~integral component of membrane | 308 | 8.80E-21 | 1.19E-17 |
|  | GOTERM_CC_DIRECT | GO:0009986~cell surface | 66 | 8.96E-17 | 1.55E-13 |
|  | GOTERM_CC_DIRECT | GO:0042101~T cell receptor complex | 11 | 1.86E-10 | 2.52E-07 |
|  | GOTERM_CC_DIRECT | GO:0001772~immunological synapse | 13 | 1.98E-09 | 2.68E-06 |
|  | GOTERM_CC_DIRECT | GO:0042613~MHC class II protein complex | 11 | 2.40E-09 | 3.24E-06 |
|  | GOTERM_CC_DIRECT | GO:0005576~extracellular region | 102 | 2.38E-07 | 3.21E-04 |
|  | GOTERM_CC_DIRECT | GO:0016020~membrane | 129 | 3.16E-07 | 4.28E-04 |
|  |  |  |  |  |  |
| GBM | GOTERM_CC_DIRECT | GO:0070062~extracellular exosome | 307 | 3.12E-23 | 4.63E-20 |
|  | GOTERM_CC_DIRECT | GO:0005764~lysosome | 47 | 1.90E-12 | 2.82E-09 |
|  | GOTERM_CC_DIRECT | GO:0016020~membrane | 221 | 3.48E-12 | 5.16E-09 |
|  | GOTERM_CC_DIRECT | GO:0005829~cytosol | 296 | 3.19E-10 | 4.73E-07 |
|  | GOTERM_CC_DIRECT | GO:0005886~plasma membrane | 351 | 1.05E-09 | 1.56E-06 |
|  | GOTERM_CC_DIRECT | GO:0005887~integral component of plasma membrane | 149 | 1.15E-09 | 1.70E-06 |
|  | GOTERM_CC_DIRECT | GO:0009897~external side of plasma membrane | 40 | 2.34E-09 | 3.48E-06 |
|  | GOTERM_CC_DIRECT | GO:0042101~T cell receptor complex | 10 | 5.08E-07 | 7.53E-04 |
|  | GOTERM_CC_DIRECT | GO:0005765~lysosomal membrane | 40 | 2.30E-06 | 0.003407 |
|  | GOTERM_CC_DIRECT | GO:0030670~phagocytic vesicle membrane | 16 | 3.18E-06 | 0.004717 |
|  |  |  |  |  |  |
| HNSC | GOTERM_CC_DIRECT | GO:0009897~external side of plasma membrane | 59 | 3.56E-36 | 4.73E-33 |
|  | GOTERM_CC_DIRECT | GO:0005886~plasma membrane | 278 | 2.64E-35 | 3.50E-32 |
|  | GOTERM_CC_DIRECT | GO:0005887~integral component of plasma membrane | 146 | 7.56E-35 | 1.00E-31 |
|  | GOTERM_CC_DIRECT | GO:0016021~integral component of membrane | 266 | 5.39E-15 | 7.22E-12 |
|  | GOTERM_CC_DIRECT | GO:0009986~cell surface | 58 | 3.51E-14 | 4.66E-11 |
|  | GOTERM_CC_DIRECT | GO:0042101~T cell receptor complex | 12 | 1.69E-12 | 2.24E-09 |
|  | GOTERM_CC_DIRECT | GO:0001772~immunological synapse | 13 | 6.09E-10 | 8.08E-07 |
|  | GOTERM_CC_DIRECT | GO:0042613~MHC class II protein complex | 11 | 8.78E-10 | 1.17E-06 |
|  | GOTERM_CC_DIRECT | GO:0016020~membrane | 125 | 7.32E-09 | 9.72E-06 |
|  | GOTERM_CC_DIRECT | GO:0042105~alpha-beta T cell receptor complex | 5 | 6.50E-06 | 0.008624 |
|  |  |  |  |  |  |
| KICH | GOTERM_CC_DIRECT | GO:0005886~plasma membrane | 388 | 3.12E-32 | 4.44E-29 |
|  | GOTERM_CC_DIRECT | GO:0005887~integral component of plasma membrane | 169 | 1.32E-22 | 1.88E-19 |
|  | GOTERM_CC_DIRECT | GO:0009897~external side of plasma membrane | 52 | 1.39E-19 | 1.99E-16 |
|  | GOTERM_CC_DIRECT | GO:0009986~cell surface | 79 | 4.27E-15 | 6.17E-12 |
|  | GOTERM_CC_DIRECT | GO:0005615~extracellular space | 131 | 1.25E-10 | 1.78E-07 |
|  | GOTERM_CC_DIRECT | GO:0016021~integral component of membrane | 375 | 1.44E-10 | 2.05E-07 |
|  | GOTERM_CC_DIRECT | GO:0016020~membrane | 188 | 3.67E-10 | 5.23E-07 |
|  | GOTERM_CC_DIRECT | GO:0042613~MHC class II protein complex | 12 | 5.30E-09 | 7.55E-06 |
|  | GOTERM_CC_DIRECT | GO:0071556~integral component of luminal side of endoplasmic reticulum membrane | 13 | 1.55E-08 | 2.21E-05 |
|  | GOTERM_CC_DIRECT | GO:0005576~extracellular region | 140 | 4.07E-08 | 5.81E-05 |
|  |  |  |  |  |  |
| KIRC | GOTERM_CC_DIRECT | GO:0016020~membrane | 81 | 1.94E-11 | 2.56E-08 |
|  | GOTERM_CC_DIRECT | GO:0001772~immunological synapse | 7 | 2.08E-05 | 0.027473 |
|  | GOTERM_CC_DIRECT | GO:0005886~plasma membrane | 101 | 5.33E-05 | 0.070506 |
|  | GOTERM_CC_DIRECT | GO:0070062~extracellular exosome | 75 | 5.86E-05 | 0.077496 |
|  | GOTERM_CC_DIRECT | GO:0009897~external side of plasma membrane | 13 | 3.01E-04 | 0.396613 |
|  | GOTERM_CC_DIRECT | GO:0005829~cytosol | 81 | 4.93E-04 | 0.649466 |
|  | GOTERM_CC_DIRECT | GO:0045121~membrane raft | 12 | 8.30E-04 | 1.092011 |
|  | GOTERM_CC_DIRECT | GO:0031234~extrinsic component of cytoplasmic side of plasma membrane | 7 | 0.001043 | 1.369756 |
|  | GOTERM_CC_DIRECT | GO:0005887~integral component of plasma membrane | 41 | 0.001048 | 1.377198 |
|  | GOTERM_CC_DIRECT | GO:0042105~alpha-beta T cell receptor complex | 3 | 0.002788 | 3.624395 |
|  |  |  |  |  |  |
| KIRP | GOTERM_CC_DIRECT | GO:0005886~plasma membrane | 313 | 4.97E-34 | 6.84E-31 |
|  | GOTERM_CC_DIRECT | GO:0005887~integral component of plasma membrane | 155 | 6.33E-31 | 8.72E-28 |
|  | GOTERM_CC_DIRECT | GO:0009897~external side of plasma membrane | 56 | 3.96E-29 | 5.45E-26 |
|  | GOTERM_CC_DIRECT | GO:0009986~cell surface | 63 | 1.54E-13 | 2.12E-10 |
|  | GOTERM_CC_DIRECT | GO:0016021~integral component of membrane | 298 | 1.21E-12 | 1.67E-09 |
|  | GOTERM_CC_DIRECT | GO:0042613~MHC class II protein complex | 12 | 2.15E-10 | 2.97E-07 |
|  | GOTERM_CC_DIRECT | GO:0042101~T cell receptor complex | 10 | 1.00E-08 | 1.38E-05 |
|  | GOTERM_CC_DIRECT | GO:0030669~clathrin-coated endocytic vesicle membrane | 13 | 4.91E-08 | 6.76E-05 |
|  | GOTERM_CC_DIRECT | GO:0001772~immunological synapse | 12 | 5.59E-08 | 7.70E-05 |
|  | GOTERM_CC_DIRECT | GO:0016020~membrane | 139 | 8.58E-08 | 1.18E-04 |
|  |  |  |  |  |  |
| DLBC | GOTERM_CC_DIRECT | GO:0005654~nucleoplasm | 404 | 2.56E-41 | 3.88E-38 |
|  | GOTERM_CC_DIRECT | GO:0005829~cytosol | 389 | 3.30E-20 | 5.00E-17 |
|  | GOTERM_CC_DIRECT | GO:0005743~mitochondrial inner membrane | 88 | 2.47E-16 | 3.33E-13 |
|  | GOTERM_CC_DIRECT | GO:0016020~membrane | 270 | 1.06E-15 | 1.68E-12 |
|  | GOTERM_CC_DIRECT | GO:0005747~mitochondrial respiratory chain complex I | 23 | 1.80E-12 | 2.73E-09 |
|  | GOTERM_CC_DIRECT | GO:0005737~cytoplasm | 516 | 7.75E-12 | 1.17E-08 |
|  | GOTERM_CC_DIRECT | GO:0005634~nucleus | 524 | 1.68E-10 | 2.54E-07 |
|  | GOTERM_CC_DIRECT | GO:0005739~mitochondrion | 164 | 1.04E-09 | 1.58E-06 |
|  | GOTERM_CC_DIRECT | GO:0000502~proteasome complex | 22 | 1.55E-09 | 2.35E-06 |
|  | GOTERM_CC_DIRECT | GO:0005730~nucleolus | 116 | 2.39E-09 | 3.62E-06 |
|  |  |  |  |  |  |
| LIHC | GOTERM_CC_DIRECT | GO:0005886~plasma membrane | 265 | 8.29E-34 | 1.12E-30 |
|  | GOTERM_CC_DIRECT | GO:0005887~integral component of plasma membrane | 129 | 1.75E-27 | 2.35E-24 |
|  | GOTERM_CC_DIRECT | GO:0009897~external side of plasma membrane | 47 | 4.12E-25 | 5.55E-22 |
|  | GOTERM_CC_DIRECT | GO:0042101~T cell receptor complex | 12 | 9.86E-13 | 1.33E-09 |
|  | GOTERM_CC_DIRECT | GO:0016021~integral component of membrane | 246 | 2.48E-12 | 3.34E-09 |
|  | GOTERM_CC_DIRECT | GO:0042613~MHC class II protein complex | 12 | 1.94E-11 | 2.61E-08 |
|  | GOTERM_CC_DIRECT | GO:0001772~immunological synapse | 13 | 3.46E-10 | 4.66E-07 |
|  | GOTERM_CC_DIRECT | GO:0009986~cell surface | 48 | 9.27E-10 | 1.25E-06 |
|  | GOTERM_CC_DIRECT | GO:0030669~clathrin-coated endocytic vesicle membrane | 12 | 4.96E-08 | 6.68E-05 |
|  | GOTERM_CC_DIRECT | GO:0016020~membrane | 116 | 1.10E-07 | 1.48E-04 |
|  |  |  |  |  |  |
| LGG | GOTERM_CC_DIRECT | GO:0070062~extracellular exosome | 332 | 4.79E-28 | 7.10E-25 |
|  | GOTERM_CC_DIRECT | GO:0005886~plasma membrane | 418 | 3.20E-22 | 4.75E-19 |
|  | GOTERM_CC_DIRECT | GO:0016020~membrane | 246 | 3.66E-17 | 5.43E-14 |
|  | GOTERM_CC_DIRECT | GO:0045121~membrane raft | 49 | 1.39E-14 | 2.06E-11 |
|  | GOTERM_CC_DIRECT | GO:0005829~cytosol | 317 | 2.57E-12 | 3.81E-09 |
|  | GOTERM_CC_DIRECT | GO:0030670~phagocytic vesicle membrane | 23 | 9.79E-12 | 1.45E-08 |
|  | GOTERM_CC_DIRECT | GO:0005887~integral component of plasma membrane | 160 | 2.73E-11 | 4.05E-08 |
|  | GOTERM_CC_DIRECT | GO:0005764~lysosome | 45 | 1.10E-10 | 1.63E-07 |
|  | GOTERM_CC_DIRECT | GO:0005765~lysosomal membrane | 50 | 2.21E-10 | 3.29E-07 |
|  | GOTERM_CC_DIRECT | GO:0071556~integral component of luminal side of endoplasmic reticulum membrane | 15 | 9.99E-10 | 1.48E-06 |
|  |  |  |  |  |  |
| LUAD | GOTERM_CC_DIRECT | GO:0005886~plasma membrane | 295 | 1.06E-42 | 1.42E-39 |
|  | GOTERM_CC_DIRECT | GO:0005887~integral component of plasma membrane | 151 | 1.96E-37 | 2.61E-34 |
|  | GOTERM_CC_DIRECT | GO:0009897~external side of plasma membrane | 59 | 5.55E-36 | 7.41E-33 |
|  | GOTERM_CC_DIRECT | GO:0016021~integral component of membrane | 273 | 1.87E-16 | 3.00E-13 |
|  | GOTERM_CC_DIRECT | GO:0009986~cell surface | 60 | 3.71E-15 | 4.90E-12 |
|  | GOTERM_CC_DIRECT | GO:0042613~MHC class II protein complex | 12 | 3.60E-11 | 4.81E-08 |
|  | GOTERM_CC_DIRECT | GO:0042101~T cell receptor complex | 11 | 7.27E-11 | 9.71E-08 |
|  | GOTERM_CC_DIRECT | GO:0016020~membrane | 132 | 1.15E-10 | 1.54E-07 |
|  | GOTERM_CC_DIRECT | GO:0071556~integral component of luminal side of endoplasmic reticulum membrane | 12 | 1.42E-09 | 1.89E-06 |
|  | GOTERM_CC_DIRECT | GO:0001772~immunological synapse | 11 | 1.32E-07 | 1.77E-04 |
|  |  |  |  |  |  |
| LUSC | GOTERM_CC_DIRECT | GO:0005886~plasma membrane | 422 | 7.96E-47 | 1.11E-43 |
|  | GOTERM_CC_DIRECT | GO:0009897~external side of plasma membrane | 75 | 4.59E-40 | 6.42E-37 |
|  | GOTERM_CC_DIRECT | GO:0005887~integral component of plasma membrane | 202 | 1.08E-38 | 1.51E-35 |
|  | GOTERM_CC_DIRECT | GO:0016021~integral component of membrane | 414 | 8.33E-21 | 1.17E-17 |
|  | GOTERM_CC_DIRECT | GO:0009986~cell surface | 86 | 4.25E-19 | 5.95E-16 |
|  | GOTERM_CC_DIRECT | GO:0005615~extracellular space | 136 | 1.72E-12 | 2.40E-09 |
|  | GOTERM_CC_DIRECT | GO:0042613~MHC class II protein complex | 14 | 1.07E-11 | 1.50E-08 |
|  | GOTERM_CC_DIRECT | GO:0045121~membrane raft | 39 | 2.91E-11 | 4.07E-08 |
|  | GOTERM_CC_DIRECT | GO:0005576~extracellular region | 149 | 8.75E-11 | 1.22E-07 |
|  | GOTERM_CC_DIRECT | GO:0016020~membrane | 189 | 8.83E-11 | 1.24E-07 |
|  |  |  |  |  |  |
| SKCM | GOTERM_CC_DIRECT | GO:0005886~plasma membrane | 361 | 2.29E-41 | 3.15E-38 |
|  | GOTERM_CC_DIRECT | GO:0009897~external side of plasma membrane | 65 | 7.44E-35 | 1.02E-31 |
|  | GOTERM_CC_DIRECT | GO:0005887~integral component of plasma membrane | 173 | 9.34E-34 | 1.28E-30 |
|  | GOTERM_CC_DIRECT | GO:0016021~integral component of membrane | 355 | 3.68E-19 | 5.05E-16 |
|  | GOTERM_CC_DIRECT | GO:0042101~T cell receptor complex | 14 | 2.60E-14 | 3.57E-11 |
|  | GOTERM_CC_DIRECT | GO:0009986~cell surface | 66 | 1.27E-12 | 1.75E-09 |
|  | GOTERM_CC_DIRECT | GO:0042613~MHC class II protein complex | 12 | 8.07E-10 | 1.11E-06 |
|  | GOTERM_CC_DIRECT | GO:0001772~immunological synapse | 14 | 1.42E-09 | 1.95E-06 |
|  | GOTERM_CC_DIRECT | GO:0016020~membrane | 157 | 1.36E-08 | 1.87E-05 |
|  | GOTERM_CC_DIRECT | GO:0071556~integral component of luminal side of endoplasmic reticulum membrane | 12 | 2.94E-08 | 4.04E-05 |
|  |  |  |  |  |  |
| MESO | GOTERM_CC_DIRECT | GO:0005886~plasma membrane | 240 | 1.98E-27 | 2.67E-24 |
|  | GOTERM_CC_DIRECT | GO:0005887~integral component of plasma membrane | 118 | 6.28E-24 | 8.48E-21 |
|  | GOTERM_CC_DIRECT | GO:0009897~external side of plasma membrane | 40 | 1.17E-19 | 1.58E-16 |
|  | GOTERM_CC_DIRECT | GO:0009986~cell surface | 54 | 7.51E-14 | 1.01E-10 |
|  | GOTERM_CC_DIRECT | GO:0042613~MHC class II protein complex | 12 | 1.05E-11 | 1.41E-08 |
|  | GOTERM_CC_DIRECT | GO:0016020~membrane | 117 | 2.06E-09 | 2.78E-06 |
|  | GOTERM_CC_DIRECT | GO:0001772~immunological synapse | 12 | 3.04E-09 | 4.10E-06 |
|  | GOTERM_CC_DIRECT | GO:0042101~T cell receptor complex | 9 | 2.45E-08 | 3.31E-05 |
|  | GOTERM_CC_DIRECT | GO:0071556~integral component of luminal side of endoplasmic reticulum membrane | 10 | 1.29E-07 | 1.74E-04 |
|  | GOTERM_CC_DIRECT | GO:0016021~integral component of membrane | 215 | 1.35E-07 | 1.82E-04 |
|  |  |  |  |  |  |
| UVM | GOTERM_CC_DIRECT | GO:0009897~external side of plasma membrane | 67 | 2.03E-32 | 2.89E-29 |
|  | GOTERM_CC_DIRECT | GO:0005886~plasma membrane | 383 | 1.30E-31 | 1.85E-28 |
|  | GOTERM_CC_DIRECT | GO:0005887~integral component of plasma membrane | 186 | 9.75E-31 | 1.39E-27 |
|  | GOTERM_CC_DIRECT | GO:0009986~cell surface | 86 | 3.99E-19 | 5.69E-16 |
|  | GOTERM_CC_DIRECT | GO:0016021~integral component of membrane | 394 | 1.93E-15 | 2.69E-12 |
|  | GOTERM_CC_DIRECT | GO:0016020~membrane | 201 | 4.51E-14 | 6.42E-11 |
|  | GOTERM_CC_DIRECT | GO:0042101~T cell receptor complex | 13 | 8.59E-12 | 1.22E-08 |
|  | GOTERM_CC_DIRECT | GO:0042613~MHC class II protein complex | 14 | 1.06E-11 | 1.51E-08 |
|  | GOTERM_CC_DIRECT | GO:0005615~extracellular space | 128 | 4.57E-10 | 6.51E-07 |
|  | GOTERM_CC_DIRECT | GO:0071556~integral component of luminal side of endoplasmic reticulum membrane | 14 | 1.01E-09 | 1.44E-06 |
|  |  |  |  |  |  |
| OV | GOTERM_CC_DIRECT | GO:0005886~plasma membrane | 271 | 1.44E-39 | 1.92E-36 |
|  | GOTERM_CC_DIRECT | GO:0005887~integral component of plasma membrane | 144 | 8.18E-38 | 1.09E-34 |
|  | GOTERM_CC_DIRECT | GO:0009897~external side of plasma membrane | 56 | 7.48E-35 | 9.96E-32 |
|  | GOTERM_CC_DIRECT | GO:0016021~integral component of membrane | 256 | 2.08E-17 | 2.76E-14 |
|  | GOTERM_CC_DIRECT | GO:0009986~cell surface | 59 | 3.09E-16 | 4.44E-13 |
|  | GOTERM_CC_DIRECT | GO:0042613~MHC class II protein complex | 11 | 4.02E-10 | 5.35E-07 |
|  | GOTERM_CC_DIRECT | GO:0071556~integral component of luminal side of endoplasmic reticulum membrane | 12 | 5.59E-10 | 7.44E-07 |
|  | GOTERM_CC_DIRECT | GO:0042101~T cell receptor complex | 10 | 1.07E-09 | 1.42E-06 |
|  | GOTERM_CC_DIRECT | GO:0005576~extracellular region | 95 | 2.63E-09 | 3.50E-06 |
|  | GOTERM_CC_DIRECT | GO:0030670~phagocytic vesicle membrane | 15 | 2.85E-09 | 3.79E-06 |
|  |  |  |  |  |  |
| PAAD | GOTERM_CC_DIRECT | GO:0005886~plasma membrane | 403 | 8.00E-41 | 1.12E-37 |
|  | GOTERM_CC_DIRECT | GO:0005887~integral component of plasma membrane | 201 | 3.58E-39 | 4.99E-36 |
|  | GOTERM_CC_DIRECT | GO:0009897~external side of plasma membrane | 68 | 1.01E-33 | 1.41E-30 |
|  | GOTERM_CC_DIRECT | GO:0009986~cell surface | 90 | 9.54E-22 | 1.33E-18 |
|  | GOTERM_CC_DIRECT | GO:0016021~integral component of membrane | 386 | 1.18E-14 | 1.64E-11 |
|  | GOTERM_CC_DIRECT | GO:0005615~extracellular space | 138 | 1.17E-13 | 1.63E-10 |
|  | GOTERM_CC_DIRECT | GO:0005576~extracellular region | 153 | 1.87E-12 | 2.60E-09 |
|  | GOTERM_CC_DIRECT | GO:0042613~MHC class II protein complex | 12 | 4.07E-09 | 5.67E-06 |
|  | GOTERM_CC_DIRECT | GO:0045121~membrane raft | 35 | 4.41E-09 | 6.15E-06 |
|  | GOTERM_CC_DIRECT | GO:0042101~T cell receptor complex | 11 | 5.53E-09 | 7.72E-06 |
|  |  |  |  |  |  |
| PCPG | GOTERM_CC_DIRECT | GO:0005925~focal adhesion | 73 | 4.45E-16 | 6.66E-13 |
|  | GOTERM_CC_DIRECT | GO:0031012~extracellular matrix | 61 | 1.57E-15 | 2.31E-12 |
|  | GOTERM_CC_DIRECT | GO:0070062~extracellular exosome | 273 | 6.89E-13 | 1.03E-09 |
|  | GOTERM_CC_DIRECT | GO:0005886~plasma membrane | 369 | 1.33E-12 | 1.98E-09 |
|  | GOTERM_CC_DIRECT | GO:0005615~extracellular space | 148 | 1.35E-10 | 2.00E-07 |
|  | GOTERM_CC_DIRECT | GO:0016020~membrane | 216 | 1.50E-10 | 2.23E-07 |
|  | GOTERM_CC_DIRECT | GO:0009986~cell surface | 76 | 3.18E-10 | 4.73E-07 |
|  | GOTERM_CC_DIRECT | GO:0005829~cytosol | 292 | 5.80E-09 | 8.64E-06 |
|  | GOTERM_CC_DIRECT | GO:0005581~collagen trimer | 23 | 6.76E-08 | 1.01E-04 |
|  | GOTERM_CC_DIRECT | GO:0005604~basement membrane | 20 | 4.77E-07 | 7.10E-04 |
|  |  |  |  |  |  |
| PRAD | GOTERM_CC_DIRECT | GO:0005886~plasma membrane | 330 | 2.07E-26 | 2.88E-23 |
|  | GOTERM_CC_DIRECT | GO:0009897~external side of plasma membrane | 54 | 4.09E-24 | 5.71E-21 |
|  | GOTERM_CC_DIRECT | GO:0005887~integral component of plasma membrane | 153 | 1.08E-22 | 1.51E-19 |
|  | GOTERM_CC_DIRECT | GO:0009986~cell surface | 71 | 1.65E-14 | 2.31E-11 |
|  | GOTERM_CC_DIRECT | GO:0005615~extracellular space | 128 | 2.67E-14 | 3.73E-11 |
|  | GOTERM_CC_DIRECT | GO:0016021~integral component of membrane | 331 | 6.53E-11 | 9.10E-08 |
|  | GOTERM_CC_DIRECT | GO:0070062~extracellular exosome | 203 | 1.77E-10 | 2.47E-07 |
|  | GOTERM_CC_DIRECT | GO:0042101~T cell receptor complex | 11 | 1.65E-09 | 2.30E-06 |
|  | GOTERM_CC_DIRECT | GO:0005576~extracellular region | 128 | 4.66E-09 | 6.50E-06 |
|  | GOTERM_CC_DIRECT | GO:0042613~MHC class II protein complex | 11 | 2.05E-08 | 2.86E-05 |
|  |  |  |  |  |  |
| SARC | GOTERM_CC_DIRECT | GO:0009897~external side of plasma membrane | 66 | 8.32E-27 | 1.22E-23 |
|  | GOTERM_CC_DIRECT | GO:0005886~plasma membrane | 409 | 1.66E-21 | 2.43E-18 |
|  | GOTERM_CC_DIRECT | GO:0005887~integral component of plasma membrane | 180 | 1.21E-18 | 1.77E-15 |
|  | GOTERM_CC_DIRECT | GO:0070062~extracellular exosome | 286 | 2.41E-15 | 3.59E-12 |
|  | GOTERM_CC_DIRECT | GO:0005764~lysosome | 49 | 2.82E-13 | 4.15E-10 |
|  | GOTERM_CC_DIRECT | GO:0009986~cell surface | 83 | 8.40E-13 | 1.23E-09 |
|  | GOTERM_CC_DIRECT | GO:0042101~T cell receptor complex | 14 | 2.47E-12 | 3.64E-09 |
|  | GOTERM_CC_DIRECT | GO:0016020~membrane | 221 | 3.62E-11 | 5.32E-08 |
|  | GOTERM_CC_DIRECT | GO:0071556~integral component of luminal side of endoplasmic reticulum membrane | 16 | 5.36E-11 | 7.88E-08 |
|  | GOTERM_CC_DIRECT | GO:0042613~MHC class II protein complex | 14 | 1.12E-10 | 1.65E-07 |
|  |  |  |  |  |  |
| STAD | GOTERM_CC_DIRECT | GO:0005886~plasma membrane | 413 | 1.78E-37 | 2.55E-34 |
|  | GOTERM_CC_DIRECT | GO:0005887~integral component of plasma membrane | 201 | 2.99E-35 | 4.29E-32 |
|  | GOTERM_CC_DIRECT | GO:0009897~external side of plasma membrane | 65 | 1.90E-29 | 2.73E-26 |
|  | GOTERM_CC_DIRECT | GO:0009986~cell surface | 83 | 3.37E-16 | 4.77E-13 |
|  | GOTERM_CC_DIRECT | GO:0005615~extracellular space | 148 | 5.91E-15 | 8.43E-12 |
|  | GOTERM_CC_DIRECT | GO:0031012~extracellular matrix | 55 | 2.04E-14 | 2.91E-11 |
|  | GOTERM_CC_DIRECT | GO:0005576~extracellular region | 163 | 2.50E-13 | 3.59E-10 |
|  | GOTERM_CC_DIRECT | GO:0070062~extracellular exosome | 239 | 3.81E-11 | 5.45E-08 |
|  | GOTERM_CC_DIRECT | GO:0005578~proteinaceous extracellular matrix | 46 | 5.76E-11 | 8.25E-08 |
|  | GOTERM_CC_DIRECT | GO:0016021~integral component of membrane | 383 | 6.93E-10 | 9.92E-07 |
|  |  |  |  |  |  |
| TGCT | GOTERM_CC_DIRECT | GO:0009897~external side of plasma membrane | 65 | 4.31E-29 | 6.24E-26 |
|  | GOTERM_CC_DIRECT | GO:0005886~plasma membrane | 368 | 3.65E-21 | 5.29E-18 |
|  | GOTERM_CC_DIRECT | GO:0005887~integral component of plasma membrane | 171 | 4.25E-21 | 6.16E-18 |
|  | GOTERM_CC_DIRECT | GO:0016020~membrane | 208 | 2.45E-13 | 3.55E-10 |
|  | GOTERM_CC_DIRECT | GO:0042101~T cell receptor complex | 13 | 1.75E-11 | 2.54E-08 |
|  | GOTERM_CC_DIRECT | GO:0042613~MHC class II protein complex | 14 | 2.27E-11 | 3.30E-08 |
|  | GOTERM_CC_DIRECT | GO:0005764~lysosome | 42 | 5.37E-11 | 7.79E-08 |
|  | GOTERM_CC_DIRECT | GO:0009986~cell surface | 72 | 7.57E-11 | 1.10E-07 |
|  | GOTERM_CC_DIRECT | GO:0071556~integral component of luminal side of endoplasmic reticulum membrane | 15 | 1.46E-10 | 2.11E-07 |
|  | GOTERM_CC_DIRECT | GO:0001772~immunological synapse | 15 | 1.99E-09 | 2.89E-06 |
|  |  |  |  |  |  |
| THYM | GOTERM_CC_DIRECT | GO:0005654~nucleoplasm | 668 | 7.94E-39 | 1.25E-35 |
|  | GOTERM_CC_DIRECT | GO:0005829~cytosol | 737 | 1.82E-31 | 2.86E-28 |
|  | GOTERM_CC_DIRECT | GO:0016020~membrane | 517 | 6.48E-27 | 1.02E-23 |
|  | GOTERM_CC_DIRECT | GO:0005737~cytoplasm | 998 | 1.09E-18 | 1.72E-15 |
|  | GOTERM_CC_DIRECT | GO:0070062~extracellular exosome | 589 | 3.12E-18 | 4.91E-15 |
|  | GOTERM_CC_DIRECT | GO:0005634~nucleus | 1023 | 1.23E-17 | 1.94E-14 |
|  | GOTERM_CC_DIRECT | GO:0005730~nucleolus | 213 | 2.00E-13 | 3.15E-10 |
|  | GOTERM_CC_DIRECT | GO:0005925~focal adhesion | 117 | 3.91E-13 | 6.15E-10 |
|  | GOTERM_CC_DIRECT | GO:0005840~ribosome | 63 | 3.40E-12 | 5.35E-09 |
|  | GOTERM_CC_DIRECT | GO:0005747~mitochondrial respiratory chain complex I | 26 | 6.15E-09 | 9.68E-06 |
|  |  |  |  |  |  |
| THCA | GOTERM_CC_DIRECT | GO:0016020~membrane | 148 | 3.55E-13 | 5.03E-10 |
|  | GOTERM_CC_DIRECT | GO:0009897~external side of plasma membrane | 34 | 3.04E-12 | 4.31E-09 |
|  | GOTERM_CC_DIRECT | GO:0005886~plasma membrane | 216 | 1.33E-08 | 1.88E-05 |
|  | GOTERM_CC_DIRECT | GO:0005829~cytosol | 178 | 9.91E-08 | 1.40E-04 |
|  | GOTERM_CC_DIRECT | GO:0042101~T cell receptor complex | 9 | 1.08E-07 | 1.53E-04 |
|  | GOTERM_CC_DIRECT | GO:0001772~immunological synapse | 11 | 2.70E-07 | 3.83E-04 |
|  | GOTERM_CC_DIRECT | GO:0070062~extracellular exosome | 148 | 5.76E-06 | 0.008167 |
|  | GOTERM_CC_DIRECT | GO:0005839~proteasome core complex | 7 | 8.58E-05 | 0.121456 |
|  | GOTERM_CC_DIRECT | GO:0030027~lamellipodium | 18 | 9.60E-05 | 0.135865 |
|  | GOTERM_CC_DIRECT | GO:0000502~proteasome complex | 10 | 3.43E-04 | 0.484609 |
|  |  |  |  |  |  |
| UCS | GOTERM_CC_DIRECT | GO:0005886~plasma membrane | 167 | 2.32E-17 | 3.11E-14 |
|  | GOTERM_CC_DIRECT | GO:0005887~integral component of plasma membrane | 84 | 9.17E-17 | 1.44E-13 |
|  | GOTERM_CC_DIRECT | GO:0009897~external side of plasma membrane | 31 | 6.61E-16 | 8.99E-13 |
|  | GOTERM_CC_DIRECT | GO:0016021~integral component of membrane | 176 | 5.63E-11 | 7.56E-08 |
|  | GOTERM_CC_DIRECT | GO:0009986~cell surface | 40 | 1.06E-10 | 1.43E-07 |
|  | GOTERM_CC_DIRECT | GO:0070062~extracellular exosome | 108 | 5.22E-09 | 7.01E-06 |
|  | GOTERM_CC_DIRECT | GO:0030670~phagocytic vesicle membrane | 12 | 5.97E-08 | 8.02E-05 |
|  | GOTERM_CC_DIRECT | GO:0016020~membrane | 87 | 8.43E-08 | 1.13E-04 |
|  | GOTERM_CC_DIRECT | GO:0001772~immunological synapse | 9 | 5.96E-07 | 8.01E-04 |
|  | GOTERM_CC_DIRECT | GO:0071556~integral component of luminal side of endoplasmic reticulum membrane | 8 | 2.56E-06 | 0.003435 |

**Table S4.** The top 10 most significant items of molecular functions of GMFG in 32 individual cancer type.

| Cancer type | Category | Term | Gene count | P-Value | FDR |
| --- | --- | --- | --- | --- | --- |
| LAML | GOTERM_CC_DIRECT | GO:0005515~protein binding | 140 | 1.45E-10 | 1.97E-07 |
|  | GOTERM_CC_DIRECT | GO:0044548~S100 protein binding | 4 | 3.47E-04 | 0.469239 |
|  | GOTERM_CC_DIRECT | GO:0051015~actin filament binding | 8 | 6.52E-04 | 0.879868 |
|  | GOTERM_CC_DIRECT | GO:0019904~protein domain specific binding | 9 | 0.0022 | 2.939959 |
|  | GOTERM_CC_DIRECT | GO:0046961~proton-transporting ATPase activity, rotational mechanism | 4 | 0.00284 | 3.780084 |
|  | GOTERM_CC_DIRECT | GO:0019899~enzyme binding | 10 | 0.011711 | 14.75398 |
|  | GOTERM_CC_DIRECT | GO:0003924~GTPase activity | 8 | 0.015185 | 18.72634 |
|  | GOTERM_CC_DIRECT | GO:0003700~transcription factor activity, sequence-specific DNA binding | 19 | 0.019491 | 23.41244 |
|  | GOTERM_CC_DIRECT | GO:0000980~RNA polymerase II distal enhancer sequence-specific DNA binding | 4 | 0.03494 | 38.24159 |
|  | GOTERM_CC_DIRECT | GO:0017124~SH3 domain binding | 5 | 0.042671 | 44.61888 |
|  |  |  |  |  |  |
| ACC | GOTERM_CC_DIRECT | GO:0004872~receptor activity | 46 | 3.56E-20 | 5.42E-17 |
|  | GOTERM_CC_DIRECT | GO:0030246~carbohydrate binding | 30 | 1.14E-09 | 1.74E-06 |
|  | GOTERM_CC_DIRECT | GO:0004888~transmembrane signaling receptor activity | 30 | 8.95E-09 | 1.36E-05 |
|  | GOTERM_CC_DIRECT | GO:0015026~coreceptor activity | 12 | 1.07E-08 | 1.62E-05 |
|  | GOTERM_CC_DIRECT | GO:0032395~MHC class II receptor activity | 9 | 3.18E-08 | 4.84E-05 |
|  | GOTERM_CC_DIRECT | GO:0042605~peptide antigen binding | 11 | 6.77E-08 | 1.03E-04 |
|  | GOTERM_CC_DIRECT | GO:0005102~receptor binding | 38 | 9.44E-08 | 1.44E-04 |
|  | GOTERM_CC_DIRECT | GO:0008009~chemokine activity | 12 | 2.84E-06 | 0.004323 |
|  | GOTERM_CC_DIRECT | GO:0005096~GTPase activator activity | 29 | 7.87E-06 | 0.011968 |
|  | GOTERM_CC_DIRECT | GO:0004896~cytokine receptor activity | 10 | 8.94E-06 | 0.013606 |
|  |  |  |  |  |  |
| CHOL | GOTERM_CC_DIRECT | GO:0004872~receptor activity | 49 | 2.62E-20 | 4.00E-17 |
|  | GOTERM_CC_DIRECT | GO:0030246~carbohydrate binding | 41 | 8.34E-16 | 1.35E-12 |
|  | GOTERM_CC_DIRECT | GO:0004888~transmembrane signaling receptor activity | 38 | 2.26E-12 | 3.46E-09 |
|  | GOTERM_CC_DIRECT | GO:0015026~coreceptor activity | 14 | 1.98E-10 | 3.03E-07 |
|  | GOTERM_CC_DIRECT | GO:0008009~chemokine activity | 16 | 2.53E-09 | 3.86E-06 |
|  | GOTERM_CC_DIRECT | GO:0005102~receptor binding | 39 | 8.63E-07 | 0.001319 |
|  | GOTERM_CC_DIRECT | GO:0032395~MHC class II receptor activity | 8 | 1.88E-06 | 0.002876 |
|  | GOTERM_CC_DIRECT | GO:0004896~cytokine receptor activity | 11 | 3.21E-06 | 0.004906 |
|  | GOTERM_CC_DIRECT | GO:0023026~MHC class II protein complex binding | 8 | 3.21E-06 | 0.004912 |
|  | GOTERM_CC_DIRECT | GO:0042288~MHC class I protein binding | 8 | 1.26E-05 | 0.019177 |
|  |  |  |  |  |  |
| BLCA | GOTERM_CC_DIRECT | GO:0004888~transmembrane signaling receptor activity | 13 | 1.22E-06 | 0.001609 |
|  | GOTERM_CC_DIRECT | GO:0004872~receptor activity | 13 | 1.42E-06 | 0.001863 |
|  | GOTERM_CC_DIRECT | GO:0030246~carbohydrate binding | 9 | 6.68E-04 | 0.875192 |
|  | GOTERM_CC_DIRECT | GO:0005085~guanyl-nucleotide exchange factor activity | 7 | 0.001032 | 1.348997 |
|  | GOTERM_CC_DIRECT | GO:0005102~receptor binding | 11 | 0.002404 | 3.116532 |
|  | GOTERM_CC_DIRECT | GO:0001618~virus receptor activity | 5 | 0.004787 | 6.117327 |
|  | GOTERM_CC_DIRECT | GO:0004715~non-membrane spanning protein tyrosine kinase activity | 4 | 0.010066 | 12.46239 |
|  | GOTERM_CC_DIRECT | GO:0005044~scavenger receptor activity | 4 | 0.011309 | 13.89728 |
|  | GOTERM_CC_DIRECT | GO:0005125~cytokine activity | 6 | 0.028768 | 31.88753 |
|  | GOTERM_CC_DIRECT | GO:0005525~GTP binding | 9 | 0.034156 | 36.69415 |
|  |  |  |  |  |  |
| BRCA | GOTERM_CC_DIRECT | GO:0004872~receptor activity | 46 | 1.16E-20 | 1.75E-17 |
|  | GOTERM_CC_DIRECT | GO:0005102~receptor binding | 46 | 2.21E-12 | 3.33E-09 |
|  | GOTERM_CC_DIRECT | GO:0030246~carbohydrate binding | 32 | 2.64E-11 | 3.98E-08 |
|  | GOTERM_CC_DIRECT | GO:0004888~transmembrane signaling receptor activity | 31 | 1.13E-09 | 1.71E-06 |
|  | GOTERM_CC_DIRECT | GO:0042605~peptide antigen binding | 12 | 3.35E-09 | 5.06E-06 |
|  | GOTERM_CC_DIRECT | GO:0004715~non-membrane spanning protein tyrosine kinase activity | 14 | 1.30E-08 | 1.96E-05 |
|  | GOTERM_CC_DIRECT | GO:0032395~MHC class II receptor activity | 9 | 2.55E-08 | 3.85E-05 |
|  | GOTERM_CC_DIRECT | GO:0008009~chemokine activity | 13 | 2.69E-07 | 4.06E-04 |
|  | GOTERM_CC_DIRECT | GO:0042288~MHC class I protein binding | 8 | 4.38E-06 | 0.006621 |
|  | GOTERM_CC_DIRECT | GO:0005125~cytokine activity | 22 | 5.07E-06 | 0.007652 |
|  |  |  |  |  |  |
| CESC | GOTERM_CC_DIRECT | GO:0004872~receptor activity | 42 | 2.69E-20 | 4.00E-17 |
|  | GOTERM_CC_DIRECT | GO:0004888~transmembrane signaling receptor activity | 34 | 7.56E-14 | 1.12E-10 |
|  | GOTERM_CC_DIRECT | GO:0030246~carbohydrate binding | 32 | 2.17E-13 | 3.22E-10 |
|  | GOTERM_CC_DIRECT | GO:0015026~coreceptor activity | 13 | 5.91E-11 | 8.77E-08 |
|  | GOTERM_CC_DIRECT | GO:0032395~MHC class II receptor activity | 9 | 6.00E-09 | 8.90E-06 |
|  | GOTERM_CC_DIRECT | GO:0004896~cytokine receptor activity | 12 | 1.06E-08 | 1.57E-05 |
|  | GOTERM_CC_DIRECT | GO:0042288~MHC class I protein binding | 9 | 6.28E-08 | 9.32E-05 |
|  | GOTERM_CC_DIRECT | GO:0042605~peptide antigen binding | 10 | 1.45E-07 | 2.15E-04 |
|  | GOTERM_CC_DIRECT | GO:0005102~receptor binding | 33 | 1.59E-07 | 2.37E-04 |
|  | GOTERM_CC_DIRECT | GO:0050839~cell adhesion molecule binding | 13 | 5.74E-07 | 8.52E-04 |
|  |  |  |  |  |  |
| COADREAD | GOTERM_CC_DIRECT | GO:0004872~receptor activity | 65 | 1.06E-23 | 1.69E-20 |
|  | GOTERM_CC_DIRECT | GO:0030246~carbohydrate binding | 51 | 7.04E-16 | 1.07E-12 |
|  | GOTERM_CC_DIRECT | GO:0008201~heparin binding | 44 | 1.09E-14 | 1.73E-11 |
|  | GOTERM_CC_DIRECT | GO:0005178~integrin binding | 34 | 1.08E-13 | 1.72E-10 |
|  | GOTERM_CC_DIRECT | GO:0005201~extracellular matrix structural constituent | 26 | 1.34E-12 | 2.14E-09 |
|  | GOTERM_CC_DIRECT | GO:0005102~receptor binding | 63 | 2.24E-11 | 3.57E-08 |
|  | GOTERM_CC_DIRECT | GO:0004888~transmembrane signaling receptor activity | 45 | 1.08E-10 | 1.72E-07 |
|  | GOTERM_CC_DIRECT | GO:0005518~collagen binding | 22 | 3.40E-10 | 5.41E-07 |
|  | GOTERM_CC_DIRECT | GO:0004896~cytokine receptor activity | 17 | 6.97E-10 | 1.11E-06 |
|  | GOTERM_CC_DIRECT | GO:0008009~chemokine activity | 19 | 2.53E-09 | 4.03E-06 |
|  |  |  |  |  |  |
| UCEC | GOTERM_CC_DIRECT | GO:0004872~receptor activity | 37 | 4.89E-18 | 7.19E-15 |
|  | GOTERM_CC_DIRECT | GO:0004888~transmembrane signaling receptor activity | 27 | 3.48E-10 | 5.12E-07 |
|  | GOTERM_CC_DIRECT | GO:0015026~coreceptor activity | 11 | 5.03E-09 | 7.40E-06 |
|  | GOTERM_CC_DIRECT | GO:0030246~carbohydrate binding | 23 | 3.46E-08 | 5.09E-05 |
|  | GOTERM_CC_DIRECT | GO:0004896~cytokine receptor activity | 9 | 5.52E-06 | 0.00811 |
|  | GOTERM_CC_DIRECT | GO:0005102~receptor binding | 27 | 7.96E-06 | 0.011696 |
|  | GOTERM_CC_DIRECT | GO:0042288~MHC class I protein binding | 7 | 9.44E-06 | 0.013874 |
|  | GOTERM_CC_DIRECT | GO:0050839~cell adhesion molecule binding | 10 | 5.44E-05 | 0.079944 |
|  | GOTERM_CC_DIRECT | GO:0005164~tumor necrosis factor receptor binding | 7 | 1.30E-04 | 0.191198 |
|  | GOTERM_CC_DIRECT | GO:0004715~non-membrane spanning protein tyrosine kinase activity | 8 | 2.73E-04 | 0.400545 |
|  |  |  |  |  |  |
| ESCA | GOTERM_CC_DIRECT | GO:0004872~receptor activity | 47 | 2.14E-22 | 3.21E-19 |
|  | GOTERM_CC_DIRECT | GO:0004888~transmembrane signaling receptor activity | 36 | 1.15E-13 | 1.73E-10 |
|  | GOTERM_CC_DIRECT | GO:0030246~carbohydrate binding | 32 | 7.40E-12 | 1.11E-08 |
|  | GOTERM_CC_DIRECT | GO:0015026~coreceptor activity | 14 | 1.46E-11 | 2.18E-08 |
|  | GOTERM_CC_DIRECT | GO:0032395~MHC class II receptor activity | 8 | 4.58E-07 | 6.87E-04 |
|  | GOTERM_CC_DIRECT | GO:0004896~cytokine receptor activity | 11 | 4.79E-07 | 7.19E-04 |
|  | GOTERM_CC_DIRECT | GO:0001618~virus receptor activity | 14 | 1.48E-06 | 0.002214 |
|  | GOTERM_CC_DIRECT | GO:0005164~tumor necrosis factor receptor binding | 9 | 7.27E-06 | 0.010905 |
|  | GOTERM_CC_DIRECT | GO:0023026~MHC class II protein complex binding | 7 | 1.47E-05 | 0.021983 |
|  | GOTERM_CC_DIRECT | GO:0019864~IgG binding | 6 | 2.63E-05 | 0.039404 |
|  |  |  |  |  |  |
| GBM | GOTERM_CC_DIRECT | GO:0004872~receptor activity | 46 | 1.78E-12 | 2.85E-09 |
|  | GOTERM_CC_DIRECT | GO:0005515~protein binding | 671 | 1.76E-11 | 2.83E-08 |
|  | GOTERM_CC_DIRECT | GO:0004197~cysteine-type endopeptidase activity | 18 | 1.74E-07 | 2.79E-04 |
|  | GOTERM_CC_DIRECT | GO:0008009~chemokine activity | 13 | 4.68E-05 | 0.075064 |
|  | GOTERM_CC_DIRECT | GO:0004896~cytokine receptor activity | 11 | 6.30E-05 | 0.100878 |
|  | GOTERM_CC_DIRECT | GO:0004888~transmembrane signaling receptor activity | 30 | 1.12E-04 | 0.179595 |
|  | GOTERM_CC_DIRECT | GO:0032395~MHC class II receptor activity | 7 | 2.10E-04 | 0.336486 |
|  | GOTERM_CC_DIRECT | GO:0023026~MHC class II protein complex binding | 7 | 3.18E-04 | 0.50878 |
|  | GOTERM_CC_DIRECT | GO:0005102~receptor binding | 41 | 3.44E-04 | 0.549874 |
|  | GOTERM_CC_DIRECT | GO:0019864~IgG binding | 6 | 3.62E-04 | 0.578847 |
|  |  |  |  |  |  |
| HNSC | GOTERM_CC_DIRECT | GO:0004872~receptor activity | 40 | 6.24E-18 | 9.31E-15 |
|  | GOTERM_CC_DIRECT | GO:0030246~carbohydrate binding | 32 | 6.77E-13 | 1.01E-09 |
|  | GOTERM_CC_DIRECT | GO:0004888~transmembrane signaling receptor activity | 32 | 7.29E-12 | 1.09E-08 |
|  | GOTERM_CC_DIRECT | GO:0015026~coreceptor activity | 13 | 9.72E-11 | 1.45E-07 |
|  | GOTERM_CC_DIRECT | GO:0004896~cytokine receptor activity | 12 | 1.65E-08 | 2.46E-05 |
|  | GOTERM_CC_DIRECT | GO:0032395~MHC class II receptor activity | 8 | 2.45E-07 | 3.66E-04 |
|  | GOTERM_CC_DIRECT | GO:0042605~peptide antigen binding | 9 | 2.75E-06 | 0.004106 |
|  | GOTERM_CC_DIRECT | GO:0045028~G-protein coupled purinergic nucleotide receptor activity | 7 | 3.44E-06 | 0.005135 |
|  | GOTERM_CC_DIRECT | GO:0001618~virus receptor activity | 13 | 3.48E-06 | 0.005199 |
|  | GOTERM_CC_DIRECT | GO:0005102~receptor binding | 31 | 3.62E-06 | 0.005398 |
|  |  |  |  |  |  |
| KICH | GOTERM_CC_DIRECT | GO:0004872~receptor activity | 44 | 1.86E-13 | 2.93E-10 |
|  | GOTERM_CC_DIRECT | GO:0030246~carbohydrate binding | 35 | 2.55E-09 | 4.00E-06 |
|  | GOTERM_CC_DIRECT | GO:0042605~peptide antigen binding | 13 | 1.02E-08 | 1.61E-05 |
|  | GOTERM_CC_DIRECT | GO:0004888~transmembrane signaling receptor activity | 34 | 8.47E-08 | 1.33E-04 |
|  | GOTERM_CC_DIRECT | GO:0042288~MHC class I protein binding | 10 | 2.61E-07 | 4.10E-04 |
|  | GOTERM_CC_DIRECT | GO:0005102~receptor binding | 45 | 3.86E-07 | 6.07E-04 |
|  | GOTERM_CC_DIRECT | GO:0032395~MHC class II receptor activity | 9 | 3.87E-07 | 6.08E-04 |
|  | GOTERM_CC_DIRECT | GO:0005096~GTPase activator activity | 38 | 7.13E-07 | 0.00112 |
|  | GOTERM_CC_DIRECT | GO:0017124~SH3 domain binding | 21 | 7.87E-06 | 0.012368 |
|  | GOTERM_CC_DIRECT | GO:0008009~chemokine activity | 13 | 1.06E-05 | 0.016592 |
|  |  |  |  |  |  |
| KIRC | GOTERM_CC_DIRECT | GO:0005096~GTPase activator activity | 17 | 2.99E-05 | 0.042182 |
|  | GOTERM_CC_DIRECT | GO:0003779~actin binding | 16 | 1.05E-04 | 0.148269 |
|  | GOTERM_CC_DIRECT | GO:0004715~non-membrane spanning protein tyrosine kinase activity | 7 | 1.33E-04 | 0.18779 |
|  | GOTERM_CC_DIRECT | GO:0005102~receptor binding | 17 | 4.52E-04 | 0.636205 |
|  | GOTERM_CC_DIRECT | GO:0005515~protein binding | 178 | 0.001797 | 2.505792 |
|  | GOTERM_CC_DIRECT | GO:0004713~protein tyrosine kinase activity | 9 | 0.00221 | 3.073967 |
|  | GOTERM_CC_DIRECT | GO:0043548~phosphatidylinositol 3-kinase binding | 4 | 0.004042 | 5.55459 |
|  | GOTERM_CC_DIRECT | GO:0005057~receptor signaling protein activity | 5 | 0.004911 | 6.710661 |
|  | GOTERM_CC_DIRECT | GO:0005031~tumor necrosis factor-activated receptor activity | 4 | 0.007923 | 10.61588 |
|  | GOTERM_CC_DIRECT | GO:0051015~actin filament binding | 8 | 0.008108 | 10.85103 |
|  |  |  |  |  |  |
| KIRP | GOTERM_CC_DIRECT | GO:0004872~receptor activity | 42 | 6.24E-17 | 1.67E-13 |
|  | GOTERM_CC_DIRECT | GO:0004888~transmembrane signaling receptor activity | 34 | 2.81E-11 | 4.32E-08 |
|  | GOTERM_CC_DIRECT | GO:0030246~carbohydrate binding | 31 | 2.72E-10 | 4.18E-07 |
|  | GOTERM_CC_DIRECT | GO:0015026~coreceptor activity | 12 | 1.10E-08 | 1.69E-05 |
|  | GOTERM_CC_DIRECT | GO:0032395~MHC class II receptor activity | 9 | 3.26E-08 | 5.00E-05 |
|  | GOTERM_CC_DIRECT | GO:0042605~peptide antigen binding | 11 | 6.96E-08 | 1.07E-04 |
|  | GOTERM_CC_DIRECT | GO:0001618~virus receptor activity | 14 | 3.62E-06 | 0.005558 |
|  | GOTERM_CC_DIRECT | GO:0005102~receptor binding | 34 | 6.14E-06 | 0.00942 |
|  | GOTERM_CC_DIRECT | GO:0005096~GTPase activator activity | 29 | 8.31E-06 | 0.012757 |
|  | GOTERM_CC_DIRECT | GO:0004715~non-membrane spanning protein tyrosine kinase activity | 11 | 1.12E-05 | 0.01724 |
|  |  |  |  |  |  |
| DLBC | GOTERM_CC_DIRECT | GO:0005515~protein binding | 923 | 1.68E-39 | 2.70E-36 |
|  | GOTERM_CC_DIRECT | GO:0044822~poly(A) RNA binding | 166 | 2.65E-15 | 4.30E-12 |
|  | GOTERM_CC_DIRECT | GO:0003735~structural constituent of ribosome | 53 | 7.13E-13 | 1.15E-09 |
|  | GOTERM_CC_DIRECT | GO:0008137~NADH dehydrogenase (ubiquinone) activity | 20 | 1.31E-09 | 2.11E-06 |
|  | GOTERM_CC_DIRECT | GO:0003713~transcription coactivator activity | 44 | 8.32E-07 | 0.001342 |
|  | GOTERM_CC_DIRECT | GO:0003954~NADH dehydrogenase activity | 7 | 5.95E-06 | 0.009593 |
|  | GOTERM_CC_DIRECT | GO:0003743~translation initiation factor activity | 17 | 1.46E-05 | 0.023472 |
|  | GOTERM_CC_DIRECT | GO:0004298~threonine-type endopeptidase activity | 10 | 1.46E-05 | 0.023572 |
|  | GOTERM_CC_DIRECT | GO:0003723~RNA binding | 72 | 2.30E-05 | 0.037093 |
|  | GOTERM_CC_DIRECT | GO:0019843~rRNA binding | 13 | 4.01E-05 | 0.064602 |
|  |  |  |  |  |  |
| LIHC | GOTERM_CC_DIRECT | GO:0004872~receptor activity | 36 | 3.24E-15 | 4.80E-12 |
|  | GOTERM_CC_DIRECT | GO:0030246~carbohydrate binding | 27 | 1.03E-09 | 1.53E-06 |
|  | GOTERM_CC_DIRECT | GO:0032395~MHC class II receptor activity | 9 | 6.00E-09 | 8.93E-06 |
|  | GOTERM_CC_DIRECT | GO:0004888~transmembrane signaling receptor activity | 27 | 6.81E-09 | 1.01E-05 |
|  | GOTERM_CC_DIRECT | GO:0005102~receptor binding | 35 | 1.46E-08 | 2.17E-05 |
|  | GOTERM_CC_DIRECT | GO:0015026~coreceptor activity | 10 | 2.83E-07 | 4.21E-04 |
|  | GOTERM_CC_DIRECT | GO:0023026~MHC class II protein complex binding | 7 | 6.76E-06 | 0.010069 |
|  | GOTERM_CC_DIRECT | GO:0004896~cytokine receptor activity | 9 | 1.55E-05 | 0.023029 |
|  | GOTERM_CC_DIRECT | GO:0042288~MHC class I protein binding | 7 | 2.11E-05 | 0.031399 |
|  | GOTERM_CC_DIRECT | GO:0003779~actin binding | 24 | 3.84E-05 | 0.0571 |
|  |  |  |  |  |  |
| LGG | GOTERM_CC_DIRECT | GO:0005515~protein binding | 720 | 4.94E-15 | 8.03E-12 |
|  | GOTERM_CC_DIRECT | GO:0042605~peptide antigen binding | 14 | 8.18E-09 | 1.31E-05 |
|  | GOTERM_CC_DIRECT | GO:0004872~receptor activity | 39 | 5.23E-08 | 8.39E-05 |
|  | GOTERM_CC_DIRECT | GO:0004871~signal transducer activity | 36 | 2.91E-07 | 4.68E-04 |
|  | GOTERM_CC_DIRECT | GO:0032395~MHC class II receptor activity | 9 | 1.77E-06 | 0.002843 |
|  | GOTERM_CC_DIRECT | GO:0004197~cysteine-type endopeptidase activity | 17 | 1.89E-06 | 0.003032 |
|  | GOTERM_CC_DIRECT | GO:0030246~carbohydrate binding | 33 | 2.82E-06 | 0.004535 |
|  | GOTERM_CC_DIRECT | GO:0042803~protein homodimerization activity | 82 | 6.16E-06 | 0.009888 |
|  | GOTERM_CC_DIRECT | GO:0005102~receptor binding | 47 | 1.34E-05 | 0.021585 |
|  | GOTERM_CC_DIRECT | GO:0004896~cytokine receptor activity | 12 | 1.59E-05 | 0.025447 |
|  |  |  |  |  |  |
| LUAD | GOTERM_CC_DIRECT | GO:0004872~receptor activity | 40 | 6.24E-18 | 9.31E-15 |
|  | GOTERM_CC_DIRECT | GO:0030246~carbohydrate binding | 32 | 6.77E-13 | 1.01E-09 |
|  | GOTERM_CC_DIRECT | GO:0004888~transmembrane signaling receptor activity | 33 | 1.38E-12 | 2.06E-09 |
|  | GOTERM_CC_DIRECT | GO:0015026~coreceptor activity | 12 | 1.80E-09 | 2.69E-06 |
|  | GOTERM_CC_DIRECT | GO:0032395~MHC class II receptor activity | 9 | 8.40E-09 | 1.25E-05 |
|  | GOTERM_CC_DIRECT | GO:0005102~receptor binding | 36 | 1.26E-08 | 1.88E-05 |
|  | GOTERM_CC_DIRECT | GO:0042605~peptide antigen binding | 11 | 1.35E-08 | 2.02E-05 |
|  | GOTERM_CC_DIRECT | GO:0004896~cytokine receptor activity | 11 | 2.05E-07 | 3.06E-04 |
|  | GOTERM_CC_DIRECT | GO:0042288~MHC class I protein binding | 8 | 1.71E-06 | 0.002548 |
|  | GOTERM_CC_DIRECT | GO:0045028~G-protein coupled purinergic nucleotide receptor activity | 7 | 3.44E-06 | 0.005137 |
|  |  |  |  |  |  |
| LUSC | GOTERM_CC_DIRECT | GO:0004872~receptor activity | 53 | 1.99E-20 | 3.12E-17 |
|  | GOTERM_CC_DIRECT | GO:0030246~carbohydrate binding | 45 | 2.33E-16 | 3.44E-13 |
|  | GOTERM_CC_DIRECT | GO:0004888~transmembrane signaling receptor activity | 42 | 8.43E-13 | 1.32E-09 |
|  | GOTERM_CC_DIRECT | GO:0005102~receptor binding | 54 | 9.50E-12 | 1.49E-08 |
|  | GOTERM_CC_DIRECT | GO:0042605~peptide antigen binding | 15 | 3.00E-11 | 4.70E-08 |
|  | GOTERM_CC_DIRECT | GO:0008009~chemokine activity | 17 | 2.66E-09 | 4.16E-06 |
|  | GOTERM_CC_DIRECT | GO:0032395~MHC class II receptor activity | 10 | 1.34E-08 | 2.10E-05 |
|  | GOTERM_CC_DIRECT | GO:0015026~coreceptor activity | 13 | 1.90E-08 | 2.97E-05 |
|  | GOTERM_CC_DIRECT | GO:0001618~virus receptor activity | 19 | 1.93E-08 | 3.02E-05 |
|  | GOTERM_CC_DIRECT | GO:0004896~cytokine receptor activity | 14 | 2.08E-08 | 3.26E-05 |
|  |  |  |  |  |  |
| SKCM | GOTERM_CC_DIRECT | GO:0004872~receptor activity | 49 | 2.92E-20 | 4.47E-17 |
|  | GOTERM_CC_DIRECT | GO:0004888~transmembrane signaling receptor activity | 37 | 1.11E-11 | 1.70E-08 |
|  | GOTERM_CC_DIRECT | GO:0005102~receptor binding | 48 | 4.36E-11 | 6.69E-08 |
|  | GOTERM_CC_DIRECT | GO:0030246~carbohydrate binding | 33 | 3.45E-10 | 5.28E-07 |
|  | GOTERM_CC_DIRECT | GO:0042605~peptide antigen binding | 12 | 1.82E-08 | 2.78E-05 |
|  | GOTERM_CC_DIRECT | GO:0008009~chemokine activity | 15 | 2.40E-08 | 3.68E-05 |
|  | GOTERM_CC_DIRECT | GO:0004896~cytokine receptor activity | 13 | 3.54E-08 | 5.42E-05 |
|  | GOTERM_CC_DIRECT | GO:0032395~MHC class II receptor activity | 9 | 9.01E-08 | 1.38E-04 |
|  | GOTERM_CC_DIRECT | GO:0005164~tumor necrosis factor receptor binding | 11 | 3.47E-07 | 5.31E-04 |
|  | GOTERM_CC_DIRECT | GO:0042288~MHC class I protein binding | 9 | 8.99E-07 | 0.001377 |
|  |  |  |  |  |  |
| MESO | GOTERM_CC_DIRECT | GO:0004872~receptor activity | 31 | 4.91E-12 | 7.34E-09 |
|  | GOTERM_CC_DIRECT | GO:0030246~carbohydrate binding | 28 | 6.33E-11 | 9.46E-08 |
|  | GOTERM_CC_DIRECT | GO:0032395~MHC class II receptor activity | 10 | 9.56E-11 | 1.43E-07 |
|  | GOTERM_CC_DIRECT | GO:0004888~transmembrane signaling receptor activity | 23 | 7.52E-07 | 0.001125 |
|  | GOTERM_CC_DIRECT | GO:0004715~non-membrane spanning protein tyrosine kinase activity | 11 | 1.02E-06 | 0.001532 |
|  | GOTERM_CC_DIRECT | GO:0005096~GTPase activator activity | 26 | 1.76E-06 | 0.002632 |
|  | GOTERM_CC_DIRECT | GO:0042605~peptide antigen binding | 8 | 1.66E-05 | 0.024863 |
|  | GOTERM_CC_DIRECT | GO:0005102~receptor binding | 27 | 3.68E-05 | 0.054968 |
|  | GOTERM_CC_DIRECT | GO:0005070~SH3/SH2 adaptor activity | 10 | 4.72E-05 | 0.07054 |
|  | GOTERM_CC_DIRECT | GO:0023026~MHC class II protein complex binding | 6 | 8.80E-05 | 0.13158 |
|  |  |  |  |  |  |
| UVM | GOTERM_CC_DIRECT | GO:0004872~receptor activity | 46 | 5.99E-15 | 9.47E-12 |
|  | GOTERM_CC_DIRECT | GO:0030246~carbohydrate binding | 36 | 4.99E-10 | 7.87E-07 |
|  | GOTERM_CC_DIRECT | GO:0042605~peptide antigen binding | 14 | 6.33E-10 | 9.99E-07 |
|  | GOTERM_CC_DIRECT | GO:0032395~MHC class II receptor activity | 10 | 1.58E-08 | 2.49E-05 |
|  | GOTERM_CC_DIRECT | GO:0005102~receptor binding | 46 | 1.08E-07 | 1.71E-04 |
|  | GOTERM_CC_DIRECT | GO:0008009~chemokine activity | 15 | 2.20E-07 | 3.47E-04 |
|  | GOTERM_CC_DIRECT | GO:0004888~transmembrane signaling receptor activity | 31 | 2.04E-06 | 0.003214 |
|  | GOTERM_CC_DIRECT | GO:0015026~coreceptor activity | 11 | 2.53E-06 | 0.003997 |
|  | GOTERM_CC_DIRECT | GO:0005031~tumor necrosis factor-activated receptor activity | 10 | 2.63E-06 | 0.004153 |
|  | GOTERM_CC_DIRECT | GO:0001618~virus receptor activity | 16 | 4.50E-06 | 0.007101 |
|  |  |  |  |  |  |
| OV | GOTERM_CC_DIRECT | GO:0004872~receptor activity | 43 | 7.41E-22 | 1.10E-18 |
|  | GOTERM_CC_DIRECT | GO:0004888~transmembrane signaling receptor activity | 33 | 1.41E-13 | 2.10E-10 |
|  | GOTERM_CC_DIRECT | GO:0030246~carbohydrate binding | 27 | 4.28E-10 | 6.34E-07 |
|  | GOTERM_CC_DIRECT | GO:0015026~coreceptor activity | 12 | 7.43E-10 | 1.10E-06 |
|  | GOTERM_CC_DIRECT | GO:0005102~receptor binding | 36 | 1.43E-09 | 2.11E-06 |
|  | GOTERM_CC_DIRECT | GO:0008009~chemokine activity | 14 | 2.02E-09 | 3.00E-06 |
|  | GOTERM_CC_DIRECT | GO:0045028~G-protein coupled purinergic nucleotide receptor activity | 9 | 2.08E-09 | 3.09E-06 |
|  | GOTERM_CC_DIRECT | GO:0004896~cytokine receptor activity | 12 | 6.90E-09 | 1.02E-05 |
|  | GOTERM_CC_DIRECT | GO:0042605~peptide antigen binding | 10 | 1.02E-07 | 1.51E-04 |
|  | GOTERM_CC_DIRECT | GO:0032395~MHC class II receptor activity | 8 | 1.38E-07 | 2.05E-04 |
|  |  |  |  |  |  |
| PAAD | GOTERM_CC_DIRECT | GO:0004872~receptor activity | 59 | 1.40E-25 | 2.18E-22 |
|  | GOTERM_CC_DIRECT | GO:0030246~carbohydrate binding | 47 | 4.20E-18 | 6.54E-15 |
|  | GOTERM_CC_DIRECT | GO:0004888~transmembrane signaling receptor activity | 47 | 1.58E-16 | 1.78E-13 |
|  | GOTERM_CC_DIRECT | GO:0015026~coreceptor activity | 14 | 1.21E-09 | 1.88E-06 |
|  | GOTERM_CC_DIRECT | GO:0008009~chemokine activity | 16 | 1.84E-08 | 2.86E-05 |
|  | GOTERM_CC_DIRECT | GO:0032395~MHC class II receptor activity | 9 | 2.74E-07 | 4.27E-04 |
|  | GOTERM_CC_DIRECT | GO:0008201~heparin binding | 26 | 1.07E-06 | 0.001667 |
|  | GOTERM_CC_DIRECT | GO:0005102~receptor binding | 42 | 1.90E-06 | 0.002959 |
|  | GOTERM_CC_DIRECT | GO:0045028~G-protein coupled purinergic nucleotide receptor activity | 8 | 2.80E-06 | 0.004359 |
|  | GOTERM_CC_DIRECT | GO:0023026~MHC class II protein complex binding | 8 | 8.50E-06 | 0.013233 |
|  |  |  |  |  |  |
| PCPG | GOTERM_CC_DIRECT | GO:0005515~protein binding | 697 | 2.47E-13 | 3.94E-10 |
|  | GOTERM_CC_DIRECT | GO:0005518~collagen binding | 18 | 1.95E-07 | 3.11E-04 |
|  | GOTERM_CC_DIRECT | GO:0005201~extracellular matrix structural constituent | 16 | 2.39E-05 | 0.038114 |
|  | GOTERM_CC_DIRECT | GO:0005516~calmodulin binding | 29 | 4.73E-05 | 0.075507 |
|  | GOTERM_CC_DIRECT | GO:0005178~integrin binding | 20 | 5.11E-05 | 0.081604 |
|  | GOTERM_CC_DIRECT | GO:0003779~actin binding | 36 | 1.77E-04 | 0.282445 |
|  | GOTERM_CC_DIRECT | GO:0005525~GTP binding | 45 | 2.47E-04 | 0.393701 |
|  | GOTERM_CC_DIRECT | GO:0004871~signal transducer activity | 28 | 4.25E-04 | 0.67673 |
|  | GOTERM_CC_DIRECT | GO:0005102~receptor binding | 41 | 5.90E-04 | 0.938473 |
|  | GOTERM_CC_DIRECT | GO:0042803~protein homodimerization activity | 72 | 6.19E-04 | 0.983503 |
|  |  |  |  |  |  |
| PRAD | GOTERM_CC_DIRECT | GO:0004872~receptor activity | 43 | 2.01E-15 | 3.09E-12 |
|  | GOTERM_CC_DIRECT | GO:0030246~carbohydrate binding | 35 | 2.46E-11 | 3.79E-08 |
|  | GOTERM_CC_DIRECT | GO:0005102~receptor binding | 43 | 2.09E-08 | 3.22E-05 |
|  | GOTERM_CC_DIRECT | GO:0032395~MHC class II receptor activity | 9 | 9.96E-08 | 1.54E-04 |
|  | GOTERM_CC_DIRECT | GO:0048020~CCR chemokine receptor binding | 10 | 4.31E-07 | 6.65E-04 |
|  | GOTERM_CC_DIRECT | GO:0004888~transmembrane signaling receptor activity | 29 | 7.11E-07 | 0.001097 |
|  | GOTERM_CC_DIRECT | GO:0005515~protein binding | 472 | 1.78E-06 | 0.002752 |
|  | GOTERM_CC_DIRECT | GO:0042605~peptide antigen binding | 10 | 2.96E-06 | 0.004559 |
|  | GOTERM_CC_DIRECT | GO:0015026~coreceptor activity | 10 | 5.63E-06 | 0.008685 |
|  | GOTERM_CC_DIRECT | GO:0005031~tumor necrosis factor-activated receptor activity | 9 | 7.84E-06 | 0.012081 |
|  |  |  |  |  |  |
| SARC | GOTERM_CC_DIRECT | GO:0004872~receptor activity | 51 | 5.39E-15 | 8.66E-12 |
|  | GOTERM_CC_DIRECT | GO:0005515~protein binding | 686 | 5.07E-10 | 8.07E-07 |
|  | GOTERM_CC_DIRECT | GO:0042605~peptide antigen binding | 15 | 5.28E-10 | 8.41E-07 |
|  | GOTERM_CC_DIRECT | GO:0030246~carbohydrate binding | 39 | 2.08E-09 | 3.31E-06 |
|  | GOTERM_CC_DIRECT | GO:0004888~transmembrane signaling receptor activity | 39 | 2.57E-08 | 4.09E-05 |
|  | GOTERM_CC_DIRECT | GO:0008009~chemokine activity | 17 | 5.68E-08 | 9.03E-05 |
|  | GOTERM_CC_DIRECT | GO:0032395~MHC class II receptor activity | 10 | 8.79E-08 | 1.40E-04 |
|  | GOTERM_CC_DIRECT | GO:0005102~receptor binding | 52 | 1.39E-07 | 2.22E-04 |
|  | GOTERM_CC_DIRECT | GO:0005031~tumor necrosis factor-activated receptor activity | 11 | 1.39E-06 | 0.002207 |
|  | GOTERM_CC_DIRECT | GO:0001618~virus receptor activity | 18 | 2.45E-06 | 0.003894 |
|  |  |  |  |  |  |
| STAD | GOTERM_CC_DIRECT | GO:0004872~receptor activity | 52 | 7.96E-19 | 1.25E-15 |
|  | GOTERM_CC_DIRECT | GO:0030246~carbohydrate binding | 39 | 1.54E-11 | 2.42E-08 |
|  | GOTERM_CC_DIRECT | GO:0004888~transmembrane signaling receptor activity | 40 | 5.95E-11 | 9.35E-08 |
|  | GOTERM_CC_DIRECT | GO:0005102~receptor binding | 49 | 1.20E-08 | 1.89E-05 |
|  | GOTERM_CC_DIRECT | GO:0045028~G-protein coupled purinergic nucleotide receptor activity | 9 | 2.15E-07 | 3.38E-04 |
|  | GOTERM_CC_DIRECT | GO:0004896~cytokine receptor activity | 13 | 3.32E-07 | 5.21E-04 |
|  | GOTERM_CC_DIRECT | GO:0005201~extracellular matrix structural constituent | 17 | 6.56E-07 | 0.001031 |
|  | GOTERM_CC_DIRECT | GO:0050840~extracellular matrix binding | 11 | 6.99E-07 | 0.001099 |
|  | GOTERM_CC_DIRECT | GO:0008201~heparin binding | 27 | 9.51E-07 | 0.001495 |
|  | GOTERM_CC_DIRECT | GO:0005096~GTPase activator activity | 38 | 1.05E-06 | 0.001656 |
|  |  |  |  |  |  |
| TGCT | GOTERM_CC_DIRECT | GO:0004872~receptor activity | 55 | 2.65E-20 | 4.19E-17 |
|  | GOTERM_CC_DIRECT | GO:0005515~protein binding | 621 | 1.32E-12 | 2.10E-09 |
|  | GOTERM_CC_DIRECT | GO:0042605~peptide antigen binding | 15 | 9.25E-11 | 1.47E-07 |
|  | GOTERM_CC_DIRECT | GO:0004888~transmembrane signaling receptor activity | 40 | 1.89E-10 | 2.99E-07 |
|  | GOTERM_CC_DIRECT | GO:0032395~MHC class II receptor activity | 10 | 2.80E-08 | 4.44E-05 |
|  | GOTERM_CC_DIRECT | GO:0005164~tumor necrosis factor receptor binding | 13 | 3.07E-08 | 4.86E-05 |
|  | GOTERM_CC_DIRECT | GO:0008009~chemokine activity | 16 | 6.92E-08 | 1.10E-04 |
|  | GOTERM_CC_DIRECT | GO:0030246~carbohydrate binding | 33 | 1.20E-07 | 1.90E-04 |
|  | GOTERM_CC_DIRECT | GO:0004715~non-membrane spanning protein tyrosine kinase activity | 15 | 2.05E-07 | 3.25E-04 |
|  | GOTERM_CC_DIRECT | GO:0005102~receptor binding | 47 | 2.73E-07 | 4.33E-04 |
|  |  |  |  |  |  |
| THYM | GOTERM_CC_DIRECT | GO:0005515~protein binding | 1718 | 1.42E-48 | 2.42E-45 |
|  | GOTERM_CC_DIRECT | GO:0044822~poly(A) RNA binding | 282 | 2.90E-17 | 4.94E-14 |
|  | GOTERM_CC_DIRECT | GO:0003735~structural constituent of ribosome | 80 | 2.03E-13 | 3.46E-10 |
|  | GOTERM_CC_DIRECT | GO:0098641~cadherin binding involved in cell-cell adhesion | 84 | 1.26E-08 | 2.15E-05 |
|  | GOTERM_CC_DIRECT | GO:0019901~protein kinase binding | 100 | 5.63E-08 | 9.57E-05 |
|  | GOTERM_CC_DIRECT | GO:0003723~RNA binding | 130 | 5.92E-07 | 0.001006 |
|  | GOTERM_CC_DIRECT | GO:0008137~NADH dehydrogenase (ubiquinone) activity | 23 | 7.61E-07 | 0.001295 |
|  | GOTERM_CC_DIRECT | GO:0042393~histone binding | 40 | 5.20E-06 | 0.008839 |
|  | GOTERM_CC_DIRECT | GO:0005085~guanyl-nucleotide exchange factor activity | 39 | 5.62E-06 | 0.009561 |
|  | GOTERM_CC_DIRECT | GO:0019838~growth factor binding | 14 | 7.57E-05 | 0.128784 |
|  |  |  |  |  |  |
| THCA | GOTERM_CC_DIRECT | GO:0005515~protein binding | 407 | 1.03E-11 | 1.57E-08 |
|  | GOTERM_CC_DIRECT | GO:0004872~receptor activity | 27 | 1.29E-07 | 1.96E-04 |
|  | GOTERM_CC_DIRECT | GO:0004298~threonine-type endopeptidase activity | 7 | 8.33E-05 | 0.127096 |
|  | GOTERM_CC_DIRECT | GO:0003779~actin binding | 25 | 1.06E-04 | 0.161795 |
|  | GOTERM_CC_DIRECT | GO:0005096~GTPase activator activity | 25 | 1.11E-04 | 0.170117 |
|  | GOTERM_CC_DIRECT | GO:0004715~non-membrane spanning protein tyrosine kinase activity | 9 | 2.48E-04 | 0.378463 |
|  | GOTERM_CC_DIRECT | GO:0005102~receptor binding | 28 | 2.98E-04 | 0.453904 |
|  | GOTERM_CC_DIRECT | GO:0030246~carbohydrate binding | 19 | 3.56E-04 | 0.542041 |
|  | GOTERM_CC_DIRECT | GO:0042288~MHC class I protein binding | 6 | 5.09E-04 | 0.774853 |
|  | GOTERM_CC_DIRECT | GO:0005085~guanyl-nucleotide exchange factor activity | 13 | 0.001386 | 2.096141 |
|  |  |  |  |  |  |
| UCS | GOTERM_CC_DIRECT | GO:0004872~receptor activity | 29 | 3.85E-14 | 5.58E-11 |
|  | GOTERM_CC_DIRECT | GO:0030246~carbohydrate binding | 21 | 1.17E-08 | 1.71E-05 |
|  | GOTERM_CC_DIRECT | GO:0004888~transmembrane signaling receptor activity | 21 | 5.19E-08 | 7.54E-05 |
|  | GOTERM_CC_DIRECT | GO:0042605~peptide antigen binding | 8 | 1.80E-06 | 0.002609 |
|  | GOTERM_CC_DIRECT | GO:0001618~virus receptor activity | 11 | 2.72E-06 | 0.00396 |
|  | GOTERM_CC_DIRECT | GO:0004896~cytokine receptor activity | 7 | 1.17E-04 | 0.170125 |
|  | GOTERM_CC_DIRECT | GO:0005102~receptor binding | 20 | 2.97E-04 | 0.431283 |
|  | GOTERM_CC_DIRECT | GO:0031726~CCR1 chemokine receptor binding | 4 | 3.40E-04 | 0.492522 |
|  | GOTERM_CC_DIRECT | GO:0016004~phospholipase activator activity | 4 | 3.40E-04 | 0.492522 |
|  | GOTERM_CC_DIRECT | GO:0004197~cysteine-type endopeptidase activity | 8 | 3.56E-04 | 0.516373 |
